# Exploring the Effect of Methyl Jasmonate on the Expression of microRNAs Involved in Biosynthesis of Active Compounds of Rosemary Cell Suspension Cultures through RNA-Sequencing

**DOI:** 10.3390/ijms23073704

**Published:** 2022-03-28

**Authors:** Deheng Yao, Yukun Chen, Xiaoping Xu, Yuling Lin, Zhongxiong Lai

**Affiliations:** 1 Institute of Horticultural Biotechnology, Fujian Agriculture and Forestry University, Fuzhou 350002, China; yaodh337@126.com (D.Y.); cyk68@163.com (Y.C.); byxxp310107@163.com (X.X.); buliang84@163.com (Y.L.); 2 College of Food and Biological Engineering, Fujian Polytechnic Normal University, Fuqing 350300, China

**Keywords:** MeJA, *Rosmarinus officinalis* L., suspension cells, active compounds, miRNAs, RNA-seq, stirred bioreactor

## Abstract

Our aim in the experiment was to study the effects of methyl jasmonates (MeJA) on the active compounds of rosemary suspension cells, the metabolites’ change of contents under different concentrations of MeJA, including 0 (CK), 10 (M10), 50 (M50) and 100 μM MeJA (M100). The results demonstrated that MeJA treatments promoted the accumulation of rosmarinic acid (RA), carnosic acid (CA), flavonoids, jasmonate (JA), gibberellin (GA), and auxin (IAA); but reduced the accumulations of abscisic acid (ABA), salicylic acid (SA), and aspartate (Asp). In addition, 50 and 100 μM MeJA promoted the accumulation of alanine (Ala) and glutamate (Glu), and 50 μM MeJA promoted the accumulation of linoleic acid and alpha-linolenic acid in rosemary suspension cells. Comparative RNA-sequencing analysis of different concentrations of MeJA showed that a total of 30, 61, and 39 miRNAs were differentially expressed in the comparisons of CKvsM10, CKvsM50, CKvsM100, respectively. The analysis of the target genes of the differentially expressed miRNAs showed that plant hormone signal transduction, linoleic acid, and alpha-linolenic acid metabolism-related genes were significantly enriched. In addition, we found that miR160a-5p target *ARF*, miR171d_1 and miR171f_3 target *DELLA*, miR171b-3p target *ETR*, and miR156a target *BRI1*, which played a key role in rosemary suspension cells under MeJA treatments. qRT-PCR of 12 differentially expressed miRNAs and their target genes showed a high correlation between the RNA-seq and the qRT-PCR result. Amplification culture of rosemary suspension cells in a 5 L stirred bioreactor showed that cell biomass accumulation in the bioreactor was less than that in the shake flask under the same conditions, and the whole cultivation period was extended to 14 d. Taken together, MeJA promoted the synthesis of the active compounds in rosemary suspension cells in a wide concentration range via concentration-dependent differential expression patterns. This study provided an overall view of the miRNAs responding to MeJA in rosemary.

## 1. Introduction

Rosemary (*Rosmarinus officinalis* L.) is a famous ornamental and medicinal homologous plant. As an excellent natural antioxidant and preservative, rosemary had been widely used in various industries, such as the food preservation, medicine, and cosmetics industries [1]. Studies had shown that the main functional components of rosemary include flavonoids, terpenoids, phenols, for example flavonoids, RA, and CA [2]. The active ingredients of rosemary were widely used in anti-tumor, anti-cancer, anti-despondency anti-virus, anti-inflammatory activity, regulating the immune system and other activities [3,4,5,6,7,8,9]. Researchers had previously attempted to regulate the synthesis of functional metabolites using various methods. MeJA regulation was considered particularly important in changing the synthesis of plant functional metabolites in cells [10]. In addition, plant tissue and cell culture techniques were the most efficient methods for obtaining functional metabolites. Our group had established a rosemary cell suspension culture system to study the influence of MeJA treatments on functional metabolites.

Jasmonic acid (JA) and its derivatives were the key signaling molecules and played important roles in many biological processes, such as growth inhibition, senescence, wound response, plant defense, and the secondary mechanism [11]. The JA signaling was an important component in plant hormone signal transduction [12,13]. As derivatives of JA, MeJA had also been used to enhance the secondary metabolites production through eliciting defense responses in many species [14,15], such as volatile terpenoids in *Amomum villosum*, triterpene in *Euphorbia pekinensis*, and tropane alkaloids in *Hyoscyamus niger* [16,17,18]. Studies had shown that MeJA treatments could promote the synthesis of numerous metabolites in plant cell cultures, such as taxol and taxanes in *Taxus* sp. suspension cells, terpenoid indole alkaloids in *Catharanthus roseus* cells, and nicotine/phenylpropanoid conjugate in *Nicotiana tabacum* cells [19,20,21,22]. In *Salvia miltiorrhiza*, *SmJAZ8* participated in the biosynthesis of phenolic acids under MeJA treatment [23]. In *Catharanthus roseus*, protein *CrMYC2*’s regulation of *ORCA* gene expression in turn regulates a series of terpenoid indole alkaloids biosynthesis genes under MeJA treament [24]. The transcription factors AP2/ERF and bHLH cooperatively mediate jasmonate-elicited nicotine biosynthesis, which—via the JA induced signaling cascade—leads to increased nicotine biosynthesis in tobacco [25,26]. In *Artemisia annua* suspension cells, exogenous MeJA promoted the accumulation of artemisinin via significantly increased *CYP71AV1* expression [27].

MiRNAs were short non-coding RNAs with a length of 19–25 nucleotides, which played a crucial role in biological processes and stress responses [28,29]. In plants, miRNAs were post-transcriptional regulators of gene expression related to growth, development and stress, and expression of miRNAs were differently in different stages of plant development [30,31]. miRNA396b was downregulated and increased the expression of *ARF16*, thereby binding to the promoters of key terpenoid indole alkaloids pathway genes and repressed their expression in *Catharanthus roseus* cells under MeJA treatment [32]. MiRNA156 and miRNA168 were downregulated in Chinese yew (*Taxus chinensis* L.) and miRNA408 was upregulated in *Lycoris aurea* under MeJA treatment [33,34]. At present, miRNA has been attracting more and more attention, and many studies have used high-throughput sequencing technology to explain the involvement of miRNAs responding to MeJA in plants. Through these studies, more conserved and novel miRNAs have been identified in different plants [35], and expression level analysis of miRNAs were particularly important in exploring their biological functions.

At present, no study has shown the miRNA-omics of rosemary responding to MeJA. In our study, we investigated the miRNA-omics of rosemary suspension cells in responding to different concentrations of MeJA using high-throughput sequencing technology. By comparing and analyzing the sequencing data of the control and MeJA treatments groups, miRNA expression profiles were investigated, and the miRNA target genes were predicted and classified in rosemary suspension cells under different concentrations of MeJA. Our studies provided new insights to the molecular mechanisms that allow MeJA to influence plant functional metabolites and an overall view of miRNAs responding to MeJA stress of a non-model plant.

## 2. Results

### 2.1. Physiological and Biochemical Indices of Rosemary Suspension Cells under Different Concentrations of MeJA

**Figure 1:**
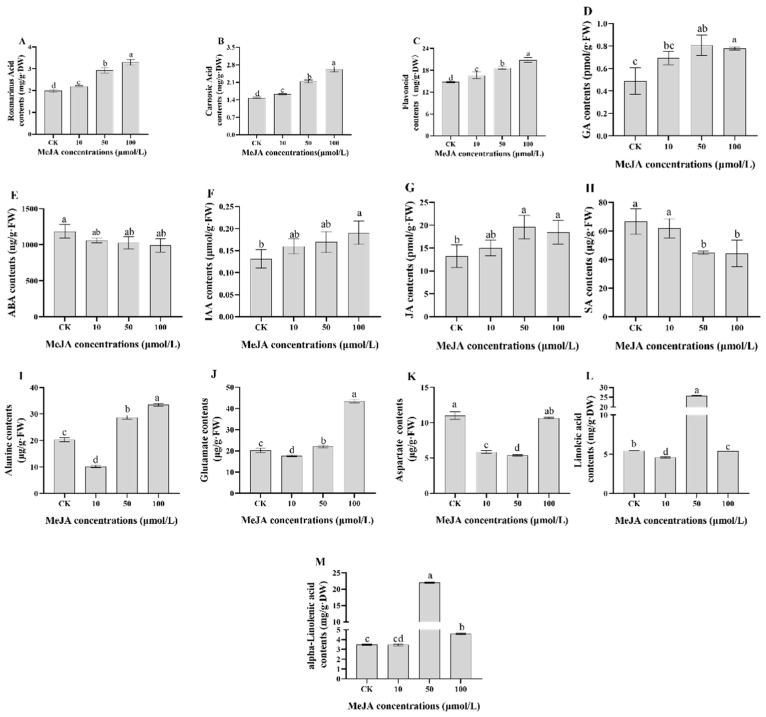
Content of physiological and biochemical indicators in rosemary suspension cells under different concentrations of MeJA for 48 h. (**A**) rosmarinic acid (mg RA/g DW); (**B**) carnosic acid (mg CA/g DW); (**C**), flavonoids (mg flavonoids/g DW); (**D**) gibberellin (pmol GA/g FW); (**E**), abscisic acid (ng ABA/g FW); (**F**) auxin (μmol IAA/g FW); (**G**) jasmonate (pmol JA /g FW); (**H**) salicylic acid (μg SA/g FW); (**I**) alanine (μg Ala/g FW); (**J**) glutamate (μg Glu/g FW); (**K**) aspartate (μg Asp/g FW); (**L**) linoleic acid (mg/g DW); (**M**) alpha-linolenic acid (mg/g DW). Different letters above the bars respectively indicate a significant difference (*p* < 0.05) from CK (0) among the CK and MeJA treatment groups. Error bars represent SDs (*n* = 3).

In our study, the effects of MeJA on rosemary suspension cells were further explained by the change of physiological and biochemical indexes, we selected 0 (CK), 10 (M10), 50 (M50), and 100 μM MeJA (M100) treatments. After suspending different concentrations of MeJA treatments for 48 h in rosemary suspension cells, the contents of RA, CA, and flavonoids were highest in the 100 μM MeJA treatment, followed by the 50 and 10 μM MeJA treatments, and the contents of the MeJA treatment groups were more than the CK group (Figure 1A–C and Tables S2–S4). The contents of JA, GA, and IAA of the MeJA treatment groups were more than the CK group, but the contents of ABA and SA of the CK group were more than the MeJA treatment groups (Figure 1D–H and Tables S5–S9). The contents of Ala and Glu of the 50 and 100 μM MeJA treatment groups were higher than the CK group, but the contents of the 10 μM MeJA treatment group were lower than the CK group; the content of Asp of the MeJA treatment groups was lower than the CK group (Figure 1I–K and Tables S10–S12). The contents of linoleic acid and alpha-linolenic acid of the 50 μM MeJA treatment group were higher than the CK group, but the contents of the 10 μM MeJA treatment group were lower than the CK group (Figure 1L,M and Tables S13 and S14). MeJA had the same promoting effect on RA, CA, flavonoids, JA, GA, and IAA, but had the opposite effect on ABA and SA. In addition, MeJA had the same effect on Ala and Glu, but had the opposite effect on Asp. Measures of 10 and 50 μM MeJA had the same effect on linoleic acid and alpha-linolenic acid. These results indicate that MeJA could promote the biosynthesis of the active compounds. Four sRNA libraries—0 (CK), 10 (M10), 50 (M50), and 100 μM MeJA (M100)—were therefore constructed and sequenced to explore miRNAs related to metabolite biosynthesis in rosemary suspension cells.

### 2.2. Small RNA Analysis of Rosemary Suspension Cells

**Figure 2:**
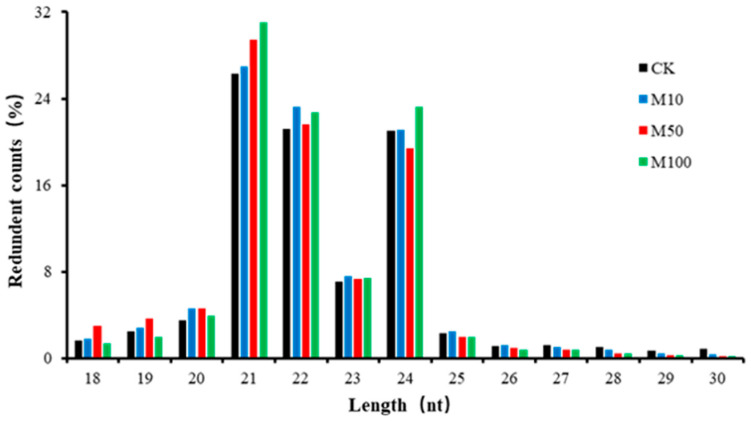
Length distribution of small RNA sequences in the small RNA libraries. Four sRNA libraries 0 (CK), 10 (M10), 50 (M50), and 100 μM MeJA (M100).

After trimming adaptor sequences and filtering out corrupted adapter sequences, the remaining reads ranging from 18 to 30 nt were selected. The clean reads of four sRNA libraries were 87.52%, 89.73%, 86.74%, and 90.81%, and the Mapped reads were 54.11%, 54.66%, 58.86%, and 56.40%, respectively, which matched the SRNA database (Table S15). The sRNA lengths of the four treatments were similar, ranging from 21–24 nt with the largest being 21 nt (Figure 2), indicating that the distribution of plant sRNA lengths did not alter in rosemary suspension cells under different concentrations of MeJA.

### 2.3. Identification of Known and Novel miRNAs of Rosemary Suspension Cells under Different Concentrations of MeJA

**Figure 3:**
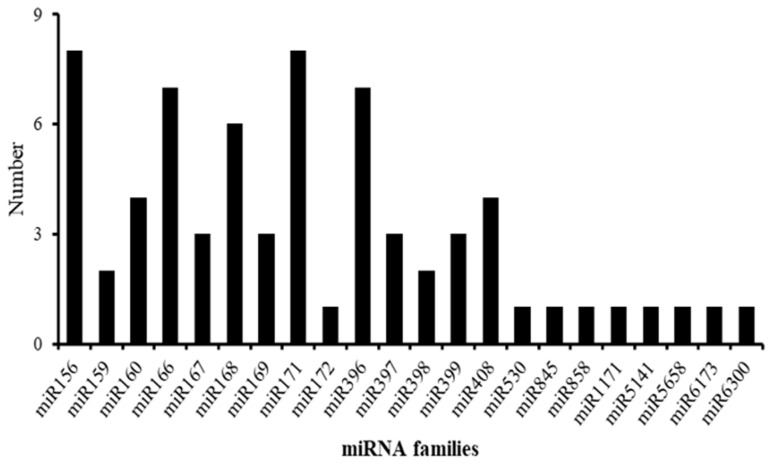
Numbers of miRNA members in each family in rosemary.

Sequencing data compared with the miRBase 22.0 database, the results were shown for 69 known miRNAs and 181 novel miRNAs in rosemary suspension cells under different concentrations of MeJA (Table S16). There were 56 mature miRNAs and 45 precursors in the CK treatment, 53 mature miRNAs and 53 precursors in the 10 μM MeJA treatment, 57 mature miRNAs and 46 precursors in the 50 μM MeJA treatment, and 53 mature miRNAs and 51 precursors in the 100 μM MeJA treatment. There were 178 mature miRNAs for the novel miRNAs in the CK treatment, 178 mature miRNAs for the novel miRNAs in the 10 μM MeJA treatment, 178 mature miRNAs for the novel miRNAs in the 50 μM MeJA treatment, and 172 mature miRNAs for the novel miRNAs in the 100 μM MeJA treatment (Tables S16 and S17). Further analysis showed that 69 known miRNAs belong to 22 miRNA families (Figure 3). The 13 miRNA families contained one or more miRNA members; for example, miR156 and miR171 had eight members. The nine miRNA families contained only one miRNA member, including miR172, miR530, miR845, miR858, miR1171, miR5141, miR5658, miR6173, and miR6300 (Table S18).

### 2.4. Differential Expression Analysis of Differentially Expressed miRNAs in Rosemary Suspension Cells under Different Concentrations of MeJA

**Figure 4:**
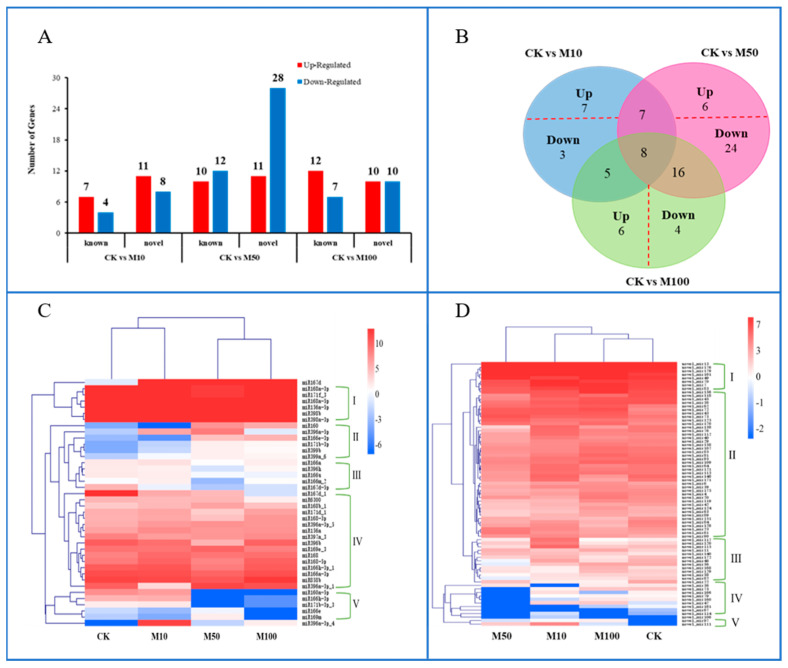
Differently expressed miRNAs in CKvsM10, CKvsM50, and CKvsM100. (**A**) the numbers of miRNAs up or downregulated in the CKvsM10, CKvsM50, and CKvsM100 (>1.5-fold and *p* < 0.05); (**B**) A Venn diagram representing the unique and common regulated miRNAs in the CKvsM10, CKvsM50, and CKvsM100; (**C**) Differentially expressed known miRNAs in response to MeJA. From the red to the blue corresponds to the numerical value of log2(TPM) from the high to the low; (**D**) Differentially expressed novel miRNAs in response to MeJA. From the red to the blue corresponds to the numerical value of log2(TPM) from the high to the low.

Whole miRNAs were analyzed to detect differentially expressed miRNAs (DEMs), revealing that 40 known miRNAs and 75 novel miRNAs expression were regulated under different concentrations of MeJA. There were 30 DEMs authenticated in CKvsM10, including seven upregulated and four downregulated known miRNAs, and 11 upregulated and eight downregulated novel miRNAs. There were 61 DEMs authenticated in CKvsM50, including 10 upregulated and 12 downregulated known miRNAs, and 11 upregulated and 28 downregulated novel miRNAs. There were 39 DEMs authenticated in CKvsM100, including 12 upregulated and seven downregulated known miRNAs, and 10 upregulated and 10 downregulated novel miRNAs (Figure 4A). Represented by Venn, there were eight miRNAs regulated by 10, 50, and 100 μM MeJA, including three known miRNAs and five novel miRNAs. There were 15 miRNAs regulated by 10 and 50 μM MeJA, including five known miRNAs; 13 miRNAs regulated by 10 and 100 μM MeJA, including five known miRNAs; and 24 miRNAs regulated by 50 and 100 μM MeJA, including 13 known miRNAs (Figure 4B). A total of 10 miRNAs were only differentially expressed in CKvsM10, of which seven were upregulated and three were downregulated; 30 miRNAs were only differentially expressed in CKvsM50, of which six were upregulated and 24 were downregulated; 10 miRNAs were only differentially expressed in CKvsM100, of which six were upregulated and four were downregulated (Figure 4B).

The hierarchical clustering of 40 differentially expressed known miRNAs under different concentrations of MeJA resulted in five major clusters (Figure 4C). The expression level of miR167d under MeJA treatment was higher than CK treatments. The six known miRNAs—including miR156a-5p, miR168a-5p, miR168a-3p, miR171f_3, miR398a-3p, and miR398b—forming cluster I displayed high expression levels in all known miRNAs, followed by cluster IV. In addition, the expression levels of 5 miRNAs belonging to cluster V, basically displayed low expression levels under 100 μM MeJA treatment. According to hierarchical cluster analysis, 75 differentially expressed novel miRNAs in different concentrations of MeJA were classified into five major clusters (Figure 4D). The eight novel miRNAs, forming cluster I displayed high expression levels in all novel miRNAs, followed by cluster II. Only two novel miRNAs (novel_mir97 and novel_mir111) belonged to cluster V, and the expression level under 100 μM MeJA treatment was lower than other treatments. These differentially expressed miRNAs analysis results revealed that rosemary suspension cells transcriptome undergoes significantly dynamic changes under different concentrations of MeJA, the datasets might serve as a valuable molecular resource for future studies.

### 2.5. Functional Classification of Differentially Expressed miRNAs in Rosemary Suspension Cells under Different Concentrations of MeJA

We performed KEGG enrichment analyses for DEMs target genes (Figure S1). The highest enrichment in CKvsM10 was plant hormone signal transduction, followed by glucosinolate biosynthesis and cyanoamino acid metabolism. The top three enrichments in CKvsM50 were linoleic acid metabolism, anthocyanin biosynthesis, and alpha-linolenic acid metabolism. The top five enrichments in CKvsM100 were plant hormone signal transduction; glucosinolate biosynthesis; alanine, aspartate, and glutamate metabolism; linoleic acid metabolism; and alpha-linolenic acid metabolism. The highest enrichment in M10vsM50, M10vsM100, and M50vsM100 was linoleic acid metabolism, followed by alpha-linolenic acid metabolism.

**Figure 5:**
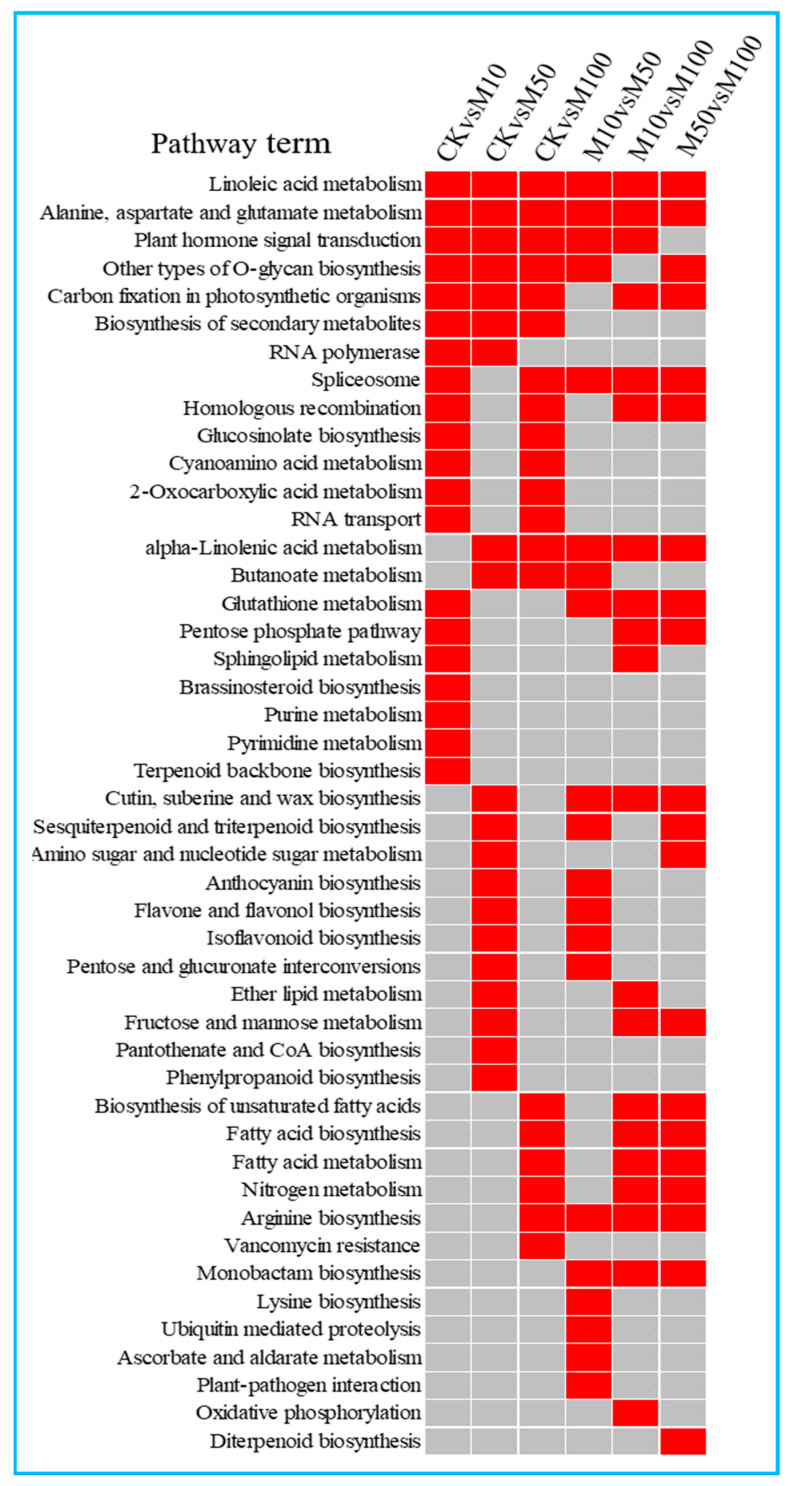
The top 20 KEGG pathways enriched by target genes of differentially expressed miRNAs in the six comparisons. Red indicates significant enrichment and gray indicates no significant enrichment.

The first 20 enrichment pathways of the six combinations (CKvsM10, CKvsM50, CKvsM100, M10vsM50, M10vsM100, M50vsM100) were compared and analyzed. Some pathways, such as linoleic acid metabolism and alanine, aspartate, and glutamate metabolism were enriched in the top 20 for all six combinations (Figure 5). Some pathways, such as plant hormone signal transduction, other types of O-glycan biosynthesis, carbon fixation in photosynthetic organisms and biosynthesis of secondary metabolites were enriched in the top 20 for all three combinations (CKvsM10, CKvsM50, CKvsM100) (Figure 5). This indicated that these pathways differed significantly under different concentrations of MeJA. Some pathways were only enriched in the top 20 for one combination (Figure 5). For example, brassinosteroid biosynthesis and terpenoid backbone biosynthesis were only enriched in the top 20 in CKvsM10, including miR156a targeted *CYP90C1* in brassinosteroid biosynthesis, novel_mir129 and novel_mir151 targeted *STE24* in terpenoid backbone biosynthesis, anthocyanin biosynthesis, flavone and flavonol biosynthesis, isoflavonoid biosynthesis, sesquiterpenoid and triterpenoid biosynthesis, and phenylpropanoid biosynthesis were only enriched in the top 20 in CKvsM50, the key gene *peroxidase* was targeted by miR167d_1 in phenylpropanoid biosynthesis, *3AT* and *IF7MAT* were targeted by novel_mir176, novel_mir11 and novel_mir179 in anthocyanin and flavone and flavonol biosynthesis, *panB* was targeted by novel_mir179, *NES1* in sesquiterpenoid and triterpenoid biosynthesis, indicating that 10 and 50 μM MeJA were more effective than 100 μM MeJA for metabolites such as terpenoids and flavonoids. Then, biosynthesis of unsaturated fatty acids, fatty acid biosynthesis, and fatty acid metabolism were only enriched in the top 20 in CKvsM100, suggesting that 100 μM MeJA was more effective for fatty acid metabolites (Figure 5).

MapMan analysis of the target genes of the differentially expressed miRNAs was distributed in the lipids, cell wall and lipids pathways in the comparisons of CKvsM10, CKvsM50, CKvsM100, respectively (Figure S2A–C). The miRNAs involved in these pathways included miR168b_1, miR167d_1, miR6300, miR396a-5p, and miR396b. The results showed that the numbers of target genes upregulated involved in cell wall, amino acids, and TCA under 10 μM MeJA treatment were higher than under other treatments, but the numbers of target genes upregulated involved in lipids under 100 μM MeJA treatment were higher than under other treatments (Figure S2D). Compared the results of CKvsM10, CKvsM50, and CKvsM100, the numbers of target genes involved in CKvsM50 and CKvsM100 were significantly more than CKvsM10 (Figure S3A–C). The miRNAs involved in the signaling included miR171d_1, miR396a-3p_1, and miR6300. The above results indicated that 50 and 100 μM MeJA affected the lipids, cell wall, and signaling more significantly than 10 μM MeJA in rosemary suspension cells.

KEGG and MapMan analyses of the target genes of the differentially expressed miRNAs showed that MeJA affected the synthesis of rosemary suspension cells’ metabolites via multiple pathways, including plant hormone signal transduction, lipids, and many other metabolism pathways.

### 2.6. Network of Differentially Expressed miRNAs and Their Targets in Rosemary Suspension Cells under Different Concentrations of MeJA

**Figure 6:**
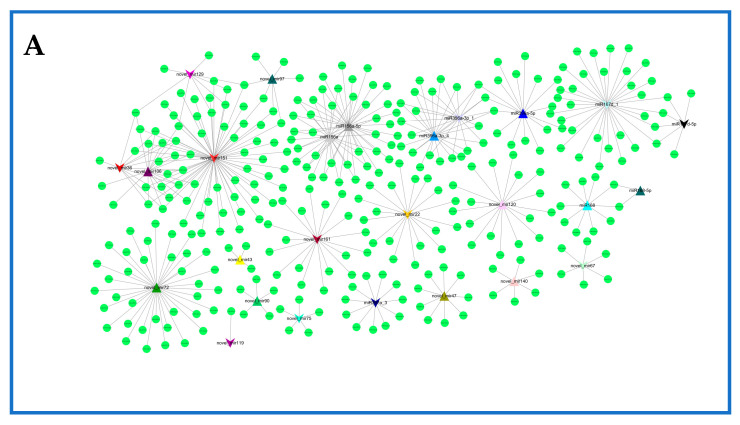

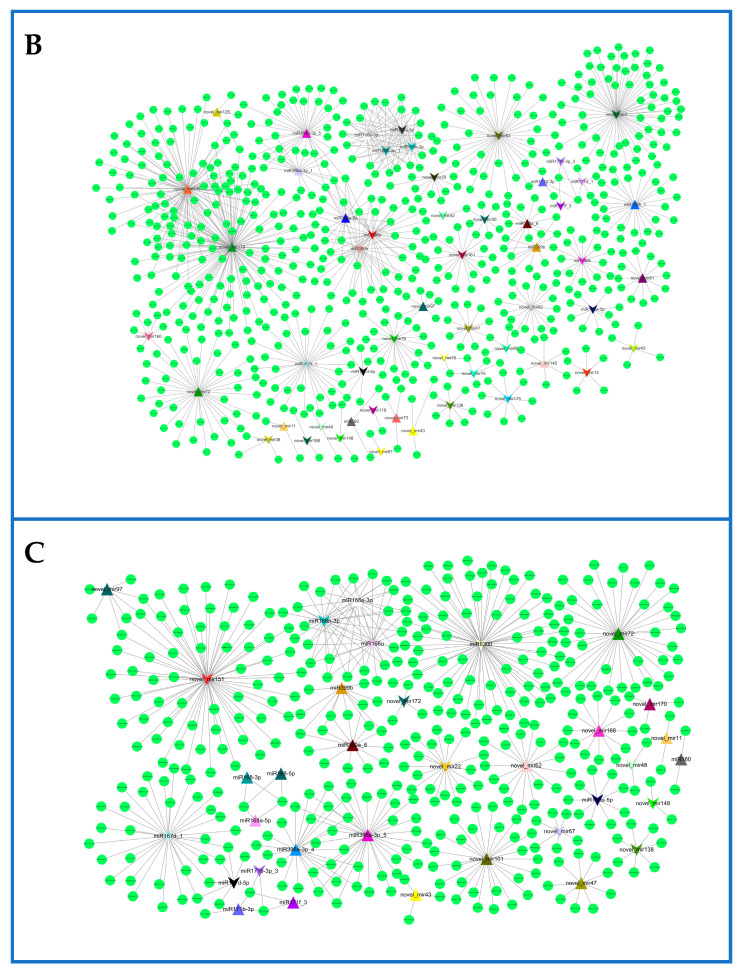
Network of MeJA-responsive miRNAs and their targets. Network analysis was performed using the Cytoscape network platform. (**A**) The interaction network in CKvsM10; (**B**) The interaction network in CKvsM50; (**C**) The interaction network in CKvsM50; Colored triangle nodes represented miRNAs, and circular nodes represented mRNAs. Solid lines indicated interaction associations between miRNAs and mRNAs.

To further understand functional metabolites and other processes associated with miRNAs responding to MeJA, we constructed the differentially expressed miRNA–mRNA interaction network using Cytoscape (Figure 6, Table S19). In CKvsM10, miR156a-5p had 38 target genes, miR167d_1 had 34 target genes, novel_mir151 had 71 target genes, and novel_mir72 had 34 target genes (Figure 6A, Table S19). The expression of miR396a-3p_4 was upregulated most substantially in all differently expressed miRNAs, and mainly involved glutathione metabolism, whereas the expression of miR171d_1 was downregulated most substantially. In CKvsM50, miR396b had 36 target genes, miR167d_1 had 34 target genes, novel_mir179 had 131 target genes, and novel_mir76 had 101 target genes (Figure 6B, Table S19). The expression of miR160 was upregulated most substantially, whereas the expression of miR160a-5p was downregulated most substantially; both of them mainly involved plant hormone signal transduction. In CKvsM100, miR6300 had 69 target genes, both miR396a-3p_5 and miR167d_1 had 34 target genes, novel_mir151 had 71 target genes, and novel_mir72 had 38 target genes (Figure 6C, Table S19). The expression of miR396a-3p_4 was upregulated most substantially in all differently expressed miRNAs, and mainly involved glutathione metabolism, whereas the expression of miR160a-5p was downregulated most substantially, and mainly involved plant hormone signal transduction. The results showed that miR156a, miR160, miR160a-5p, novel_mir151, and novel_mir72 were mainly associated with plant hormone signal transduction. Plant hormone signal transduction played an important role in rosemary suspension cells responding to MeJA.

Some target genes in CKvsM10, CKvsM50, and CKvsM100 were also regulated by different miRNAs. For example, the gene Unigene16296_All was regulated jointly by miR167d_1 and miR167d-5p; CL9049.Contig3_All was regulated jointly by miR156a and miR156a-5p; CL7970.Contig1_All was regulated jointly by miR396a-3p_1, miR396a-3p_4, and miR396a-3p_5. 

### 2.7. miRNAs and Target Genes Related to the Plant Hormone Signal Transduction in Rosemary Suspension Cells under Different Concentrations of MeJA

The above results showed that physiological and biochemical indexes and functional classification of differentially expressed miRNAs in rosemary suspension cells (Figure 1 and Figure 5), and many differentially expressed miRNAs were mainly associated with plant hormone signal transduction (Tables S20–S22). This study therefore sought to find a regulatory mechanism for miRNAs involved in the plant hormone signal transduction, which in turn affects the synthesis of the active compounds in rosemary suspension cells. We explored rosemary suspension cells miRNA-omics data in our study and found that some differentially expressed miRNAs were related to the *ARF*, *DELLA*, *ETR*, and *BRI1* in the plant hormone signal transduction. The target genes *ARF* (CL11742.Contig5_All) of miR160; *ARF* (CL7275.Contig1_All, CL8053.Contig1_All, CL11742.Contig1_All) of miR160a-5p; *DELLA* (CL4553.Contig1_All, CL3157.Contig1_All, CL3157.Contig4_All) of miR156a-5p, miR171b-3p, miR171b-3p_3, miR171d_1, and miR171f_3; *ETR* (CL1203.Contig7_All) of miR171b-3p; and *BRI1*(CL9049.Contig3_All) of miR156a and miR156a-5p were all able to participate in the plant hormone signal transduction networks (Table 1). miR156a and miR156a-5p were upregulated under 10 μM MeJA treatment; miR160 and miR171b-3p were upregulated, but miR160a-5p, miR171b-3p_3, miR171d_1, and miR171f_3 were downregulated under 50 μM MeJA treatment; miR160, miR171b-3p, and miR171f_3 were upregulated, and miR160a-5p and miR171b-3p_3 were downregulated under 100 μM MeJA treatment (Tables S20–S22).

### 2.8. Identification of Differentially Expressed miRNAs and Their Targets in Rosemary Suspension Cells under Different Concentrations of MeJA by Quantitative qRT-PCR

**Figure 7:**
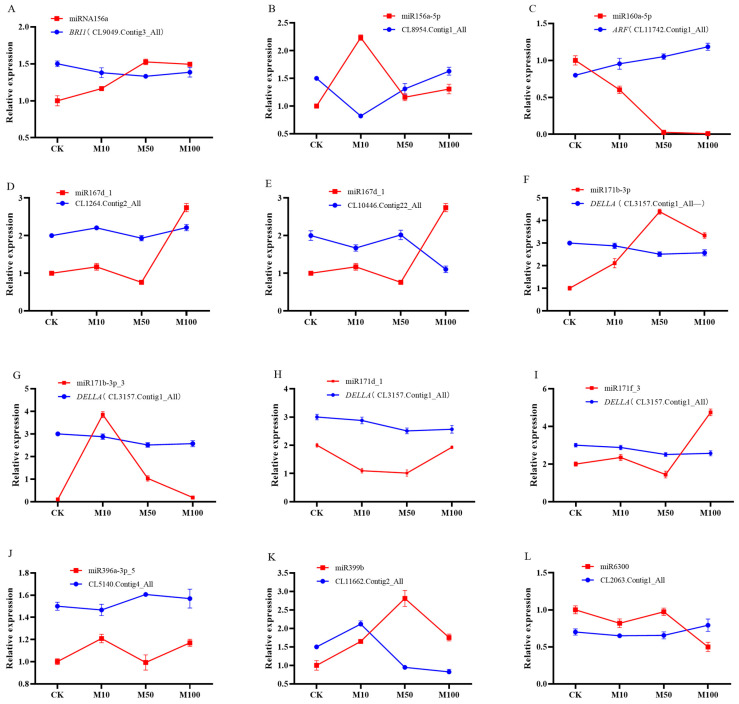
qRT-PCR verification of miRNAs and target genes in rosemary suspension cells under different concentrations of MeJA.

Twelve miRNAs and their target genes were verified by qRT-PCR (Figure 7). Among these, only the expression patterns of miR156a, miR160a-5p, miR167d-1, miR171b-3p, and miR396a-3p-5, and their target genes were negatively correlated, indicating that these five target genes were negatively regulated by their corresponding miRNAs (Figure 7A,C,E,F,J), the others miRNAs and their target genes were not negatively correlated (Figure 7B,D,G–I,K,L). For example, in the case of the common target gene CL3157.Contig1_All of miR171b-3p, miR171b-3p_3, miR171d_1, and miR171f_3, the expressions of miR171b-3p_3, miR171d_1, and miR171f_3 were not negatively correlated with their target genes, miR171b-3p will complement the function of them (Figure 7F,G–I), and these miRNAs have their own roles under different concentrations of MeJA. Although some miRNAs were not regulated the expression of mRNAs, other miRNAs or members of the same family complement their functions and ultimately regulate target genes. In addition, the differential expression changes of miRNAs are basically consistent with the sequencing results, indicating that the data results of this sequencing are accurate.

Some miRNAs were involved in plant hormone signal transduction under different concentrations of MeJA. For example, miR156a targeted CL9049.Contig3_All (*BRI1*) to regulate BR signaling transduction (Figure 7A); miR160a-5p targeted CL11742.Contig1_All (*ARF*) to regulate auxin signaling transduction (Figure 7C); miR171b-3p, miR171b-3p_3, miR171d_1, and miR171f_3 targeted CL3157.Contig1_All (*DELLA*) to regulate GA signaling transduction (Figure 7F,G–I). The expression of *BRI1* and *DELLA* was downregulated under different concentrations of MeJA, which might influence the accumulation of metabolites through negative regulation in rosemary suspension cells; the expression of *ARF* was upregulated under different concentrations of MeJA, which might influence the accumulation of metabolites through positive regulation in rosemary suspension cells.

### 2.9. Analysis of Rosemary Cells Suspension Culture in 5 L Stirred Bioreactor

**Figure 8:**
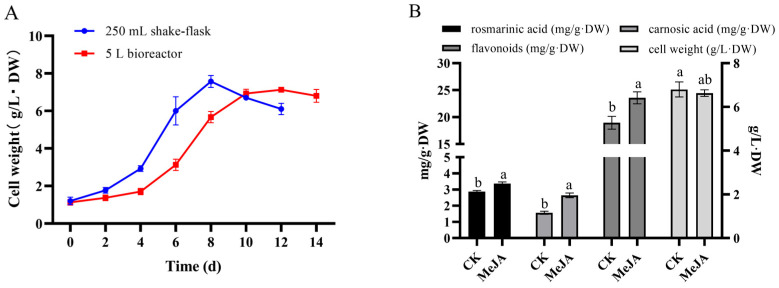
Amplification culture of rosemary suspension cells in 5 L stirring bioreactor. (**A**) Changes in the cell growth between the stirred bioreactor and shake flask; (**B**) The effects of MeJA on the rosemary suspension cells in 5 L stirred bioreactor. Different letters above the bars respectively indicate a significant difference (*p* < 0.05) among the CK and MeJA treatment.

In our study, based on the establishment of a stable rosemary suspension cells amplification culture system in stirred bioreactor, the cell growth of cell suspension culture in stirred bioreactor were researched and compared with the shake flask culture. The results of the effects of MeJA on the rosemary suspension cells in 5 L stirred bioreactor showed that cell biomass accumulation in the bioreactor and shake flask were 6.63 and 6.80 g/L·DW under the same conditions, and the whole cultivation period was extended to 14 d (Figure 8A). After 100 μM MeJA treatment for 144 h in the bioreactor, the contents of RA, CA, and flavonoids were higher than the control group (CK), and cell biomass accumulation was less than the control group (Figure 8B). The 100 μM MeJA treatment had the same effect on rosemary suspended cells for the accumulations of active compounds both in 5 L stirred bioreactor and shake flask. 

## 3. Discussion

### 3.1. miRNAs of Rosemary Suspension Cells Responding to MeJA

In our study, the size distributions of sRNAs—ranging from 18 to 30 nt—were analyzed in four sRNA libraries (CK, M10, M50, and M100), sRNAs of rosemary suspension cells ranged from 21 to 24 nt in length, with most shown to be 21 nt long, followed by 22 nt, 23 nt, and 24 nt (Figure 2). Previous studies had shown that *Vitis vinifera* and *Triticum aestivum* had the most 21 nt sRNAs; *Saccharum officinarum* had the most 22 nt sRNAs; *Cucumis* had the most 23 nt sRNAs; and *Dimocarpus longan*, *Arabidopsis thaliana*, *Solanum tuberosum*, and *Lycopersicon esculentum* had the most 24 nt sRNAs [36,37,38,39,40,41,42,43]. The expression patterns of miRNAs were differentially responding to MeJA in plants. For example, miRNA156 and miRNA168 were downregulated in *Taxus chinensis* under MeJA treatment [33]; in our study, they were also upregulated in the rosemary suspension cells under MeJA treatment. miR156-SPLs coordinated development and respond to abiotic stress by affecting the synthesis of anthocyanin in *Arabidopsis Pro35S*:*MIR156* mutant [44]. MiRNA396b was downregulated in *Catharanthus roseus* cells and miRNA408 was upregulated in *Lycoris aurea* under MeJA treatment [32,34], but miRNA396b and miRNA408 were not differentially expressed under MeJA treatment in the rosemary suspension cells. *hpo-miR396b*-*GRF6* could be involved in slat and phytohormone ABA stresses in *pitaya* under both NaCl and ABA treatment [45]. There were 11, 22, and 19 miRNAs and 19, 39, and 20 novel miRNAs were differentially expressed in the three combinations of CKvsM10 CKvsM50, and CKvsM100, respectively (Figure 4A). The result of the hierarchical clustering of 40 differentially expressed known miRNAs showed that miRNAs in the same family also had different expression patterns (Figure 4C). In summary, these miRNAs played a key role and had functional specificity and diversity in the rosemary suspension cells under different concentrations of MeJA.

### 3.2. miRNAs Target Metabolic Pathway Genes to Regulat the Synthesis of Active Compounds in Rosemary Suspension Cells under Different Concentrations of MeJA

miRNAs targeted the structural genes of metabolic pathways to play the most direct regulatory role [46]. MiR1446-x targeted *PRPOL* of ubiquinone and other terpenoid-quinone biosynthesis, miR845-y targeted *DHCR24* of steroid biosynthesis in *Euphorbia kansui* [47]. In *Camellia sinensis*, Csn-miR167a targeted flavonoid structural genes *CHI* and *ANR* to regulate the biosynthesis of flavonoid [48]. *13𝛼-hydroxylase* and *2𝛼-O-benzoyltransferase* of the paclitaxel biosynthesis pathway were targeted by miR164 and miR171, these miRNAs could regulate the biosynthesis of paclitaxel by targeting the key genes *13𝛼-hydroxylase* and *2𝛼-O-benzoyltransferase* in *Taxus chinensis* [49]. Phenylpropanoid, terpenoid, and flavonoid biosynthesis were closely related to the biosynthesis of RA, CA, and flavonoids. In our study, miR156a novel_mir129, novel_mir13, novel_mir151, and novel_mir179 were differentially expressed under MeJA treatments, their target genes were involved in terpenoid backbone, brassinosteroid, sesquiterpenoid, and triterpenoid biosynthesis (Figure 1, Figure 5 and Figure 6 and Tables S19–S21), which could promote the biosynthesis of terpenoids in the mevalonate/methylerythritol phosphate (MVA/MEP) pathway [50]. Moreover, miR167d_1, novel_mir11, and novel_mir179 were differentially expressed under different concentrations of MeJA, their target genes were involved in phenylpropanoid biosynthesis, anthocyanin, and flavone and flavonol biosynthesis, these miRNAs might regulate the biosynthesis of flavonoids in rosemary suspension cells (Figure 1, Figure 5 and Figure 6). Biotic and abiotic stress would inhibit or promote the biosynthesis of JA in plants, JA signal directly participates in the plant defense response and interacts with other signal molecules in the process to regulate plant growth and development and the synthesis of secondary metabolites [51,52]. Stress could increase the content of unsaturated fatty acids in plant cells and increase the ratio of unsaturated fatty acids so as to enhance the fluidity of membrane [53,54,55]. novel_mir90 and novel_mir22 targeted *LOX1-5*; *LOX2S* was targeted by novel_mir138 in linoleic acid metabolism and alpha-linolenic acid metabolism, they would regulate the biosynthesis of JA (Figure 1, Figure 5 and Figure 6 and Tables S20–S22). The result showed that 50 μM MeJA treatment could significantly induce the biosynthesis of linoleic acid and alpha-linolenic acid compared to 10 and 100 μM MeJA, the top three enrichments in CKvsM50 were linoleic acid metabolism, anthocyanin biosynthesis, and alpha-linolenic acid metabolism (Figure S1), and 50 μM MeJA treatment could significantly suppress the expression of novel_mir138 compared to10 and 100 μM MeJA, indicating that 50 μM MeJA could significantly affect linoleic acid and alpha-Linolenic acid metabolism in rosemary suspension cells. novel_mir72 targeted *AGXT2*, novel_mir62 targeted *argG*, miR399b targeted *gdhA*, miR6300 targeted *ALDH5A1*, novel_mir22 targeted *gadB*, and novel_mir101 targeted *CAD* in alanine, aspartate, and glutamate metabolism; *SCD* and *FAB2* were targeted by miR6300, and *PPT* was targeted bynovel_mir47 in biosynthesis of unsaturated fatty acids and fatty acid biosynthesis; *ALDH5A1* was targeted by miR399b in butanoate metabolism, these miRNAs would closely relate to the biosynthesis of amino acid terpenoids, phenol, and flavonoids in rosemary suspension cells (Figure 1, Figure 5 and Figure 6 and Tables S20–S22). In all, these differentially expressed miRNAs might target the structural genes of metabolic pathways to regulate the biosynthesis of terpenoids and flavonoids in rosemary suspension cells under different concentrations of MeJA.

### 3.3. Differentially Expressed miRNAs Involved in Plant Hormone Signal Transduction Played a Key Role in Rosemary Suspension Cells Responding to MeJA

MeJA would promote the accumulation of RA, CA, and flavonoids in rosemary suspension cells (Figure 1). The primary or secondary metabolites were precursors for the biosynthesis of endogenous hormones in plant [56,57]. External factors could change hormone concentrations to enable plants adapting to the external environment [58]. Plant hormones could regulate the biosynthesis of metabolites via complex networks, and that there were interactions between them [59]. In our study, the target genes of the differentially expressed miRNAs involved in plant hormone signal transduction—including auxin, gibberellins, ethylene, and brassinolide signal transduction (Table 1). In the three combinations of CKvsM10, CKvsM50, and CKvsM100, plant hormone signal transduction was all enriched to the top 10 (Figure 1 and Figure S1). Auxin could promote the binding of SCFTIR1 to Aux-IAA protein, thus releasing transcription factor *ARFs* to activate down-stream genes [60]. In *Arabidopsis arf6* and *arf8* single mutants and sesquimutants, *ARF6* and *ARF8* gene dosage affected the accumulation of JA and the expression of *MYB*; *JAZ1* as an inhibitor of JA signal could not be induced by auxin and depended on *ARF6* and *ARF8* [61]. In *Arabidopsis*, four miRNAs (miR156, miR165/166, miR828, and miR858) were involved in the biosynthesis of anthocyanin [62]. Overexpression of miR156 affected the biosynthesis of anthocyanin through transcription factors and anthocyanin-specific structural genes in poplar [63]. Ib-miR156 could positively mediate the biosynthesis of anthocyanin and flaxonols by modulating related structural genes, including *CHS*, *CHI*, *DFR*, and *ANS* in the *wild type* plants (WT) [64]. In our study, miR160a-5p and miR160 targeted *ARF* to regulate the growth, development, and the biosynthesis of metabolic in rosemary suspension cells under 50 and 100 μM MeJA treatments (Table 1).

*DELLA* as an inhibitor could enhance *MYC2* to induce expression of down-stream defense genes by binding to *JAZ1* [65]. *DELLA* would inhibit the transcriptional activation of down-stream target genes by binding to *PIF3* and *PIF4* [66]. *PIFS* played an important regulatory role in the synthesis of flavonoids and chlorophyll [67,68]. *DELLA-MYB12/MYB111* module could regulate the genes of biosynthesis of flavonol in *Arabidopsis* [69]. *SmGRAS3* might bind to the promoters of *AACT2*, *HMGS*, *HMGR2*, *DXS2*, *DXR*, *CMK*, *HDS*, *CPS1*, *KSL1*, *CYP76AH1*, *4CL2*, and *TAT1* to regulate the biosynthesis of tanshinones and SA in *Salvia miltiorrhiza* [70]. *ETR* as an inhibitor could activate the biosynthesis of ethylene in *Arabidopsis ethylene receptor* mutant [71]. The synergistic effect of JA and ET signaling was mediated by the interaction between *JAZ* and *EIN3/EIL1*, and the antagonistic effect might be mainly realized by the interaction between *MYC2* and *EIN3/EIL1* [72]. Ethylene could promote the biosynthesis of pyridine alkaloids and nicotine in *Nicotiana tabacum* [22] and affect the biosynthesis of plant functional metabolites in *Catharanthus roseus* and *Hevea brasiliensis* [73,74]. Overexpression of miR171b would inhibit seed germination and early plant growth, and affect plant photosynthesis in tomato leaves [75]. In our study, *DELLA* was targeted by miR156a-5p, miR171b-3p, miR171b-3p_3, miR171d_1, and miR171f_3; *PIF4* was targeted by novel_mir72; and *ETR* was targeted by miR171b-3p (Table 1)—these miRNAs might regulate the biosynthesis of terpenoids and flavonoids metabolites in rosemary suspension cells via the target gene *DELLA*, *PIF4*, and *ETR* in rosemary suspension cells under 50 and 100 μM MeJA treatments.

BR signal transduction was involved in regulating plant development and physiological metabolism [76]. BR treatment could promote the accumulation of carotenoids in tomato fruit [77]. BL enhanced the anthocyanin accumulation induced by MeJA, indicating a positive correlation between the interaction of BR and JA signal transduction in *Arabidopsis* [78]. MeJA could eliminate the increase effect on leaf angle by BL, while it inhibited the expression of genes related to BR biosynthesis and signal transduction, indicating the negative correlation between the interaction of JA and BR in rice [79]. MAPK signaling was an important factor that transmits signals from the cell surface to the interior of the nucleus [80]. *FLS2* triggers a downstream response when the external environment changes, which as an immune receptor the plasma membrane [81,82]. *FLS2* and *BRI1* both are LRR-RLK, and could form a ligand-dependent heterologous complex with *BAK1*; increasing the expression of *BRI1* would inhibit *FLS2* expression, and conversely promote *FLS2* expression, which might be involved in the balance between plant development and defense [83]. MAPK pathways were involved in hormone signal transduction in plants, including ethylene [84]. In our study, miR156a, miR156a-5p, and novel_mir4 targeted *BRI1* (Table 1); and miR396a-3p_5 and novel_mir72 targeted *FLS2* (Tables S20–S22), these miRNAs and target genes might activate signal transduction to regulate the biosynthesis of terpenoids and flavonoids metabolites in rosemary suspension cells under MeJA treatment. 

### 3.4. Amplification Culture of Rosemary Suspension Cells in 5 L Stirred Bioreactor

For large-scale plant cell culture, a variety of bioreactor types providing growth and expression of bioactive substances were available today, several bioreactor designs had been suggested [85,86]. Bentebibel used stirred, airlift, and wave bioreactors for the production of paclitaxel and baccatin III in cell suspension cultures of *Taxus baccata* [87]. Stirred bioreactor was one of the highest reported so far by an academic laboratory for plant cell bioreactor culture. Currently, bioreactors of up to 75,000 L were being employed for the commercial production of paclitaxel from cell cultures by Phyton Biotech, ESCA genetic, Samyang Genex, Nattermann (Germany) [88]. In our study, the results showed that the accumulation of cell biomass in the bioreactor was less than the shake flask under the same conditions, and the whole cultivation period was extended to 14 d (Figure 8). which were the same as those form amplification culture of *Glycyrrhiza uralensis* cells in stirred bioreactor [89]. The 100 μM MeJA treatment could promote the biosynthesis of paclitaxel and baccatin III in 5 L stirred bioreactor, the biosynthesis of paclitaxel in 4 L airlift bioreactor, and the biosynthesis of paclitaxel and baccatin III in 2 L wave bioreactor [87]. In our study, 100 μM MeJA could promote the biosynthesis of RA, CA, and flavonoids in the culture of rosemary cells in 5 L stirred bioreactor (Figure 8). The results would be an important experimental basis for the production of active compounds in large-scale culture of rosemary.

**Figure 9:**
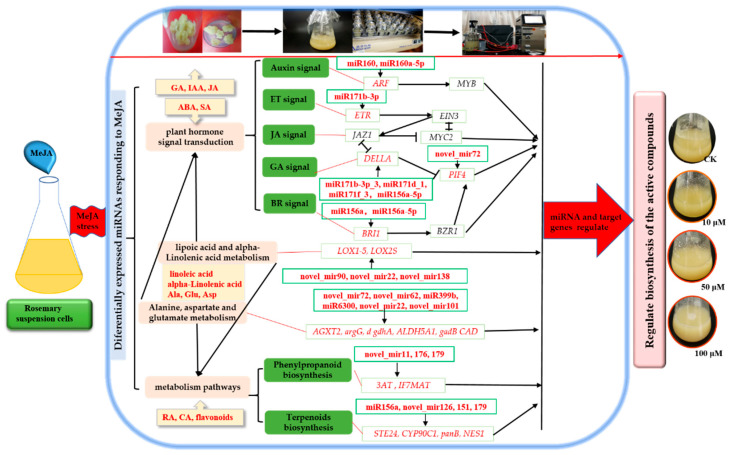
Model depicting the regulatory miRNA-mediated mechanisms of metabolite biosynthesis under MeJA. Differentially expressed miRNAs and their target genes of plant hormone signal transduction; lipoic acid metabolism; alpha-linolenic acid metabolism; alanine, aspartate and glutamate metabolism; and phenylpropanoid and terpenoids biosynthesis pathways which played an essential beneficial in rosemary suspension cells responding to MeJA. The picture shows rosemary cell culture system. The parameters, miRNAs, and target genes named in red indicate the difference of level and expression under different concentrations of MeJA.

In conclusion, MeJA promoted the accumulation of RA, CA, and flavonoids of rosemary cell suspension culture in 5 L stirred bioreactor and shake flask. A comparative analysis of differentially expressed miRNAs and their target genes in rosemary suspension cells was conducted under different concentrations of MeJA. Our study revealed the miRNAs and their target genes involved in plant hormone signaling pathways; alanine, aspartate, and glutamate metabolism; and terpenoids and phenylpropanoid biosynthesis pathways, indicating their regulatory role in the synthesis of the active compounds via the complex network in rosemary suspension cells under different concentrations of MeJA. We suggested a feasible working model based on the results (Figure 9). These RNA-seq data and amplification culture might provide new insights into future functional studies as a means of studying the molecular mechanisms on the biosynthesis of active compounds in rosemary suspension cells and an important experimental basis for the production of active compounds in large-scale culture of rosemary.

## 4. Materials and Methods

### 4.1. Plant Material and MeJA Treatments

*Rosmarinus officinalis* L. was identified by Zhong xiong Lai in our laboratory. Rosemary callus was obtained by the following methods: treating rosemary leaves with 0.1% mercury bichloride solution, then cutting the leaves into small pieces about 0.5 × 0.5 cm, using the inoculation method that the abaxial surface of leaves contacts with the medium, on solid Murashige and Skoog (MS) medium (30 g/L sucrose, pH 5.8) with 0.5 mg/L 1-naphthaleneacetic acid (NAA) and 4.0 mg/L N-(Phenylmethyl)-9H-purin-6-amine (6-BA) at 25 ± 0.5 °C in the dark for 21 days. Callus culture maintenance: 0.5 g of fresh callus were inoculated on solid MS medium with 30 g/L sucrose, pH 5.8, and supplemented with 1.0 mg/L 2,4-dichlorophenoxyacetic acid (2,4-D) and cultured at 25 °C in the dark every 21 days. Cell suspension induction and cell line maintenance: 4 g of fresh callus were inoculated into a 250 mL flask containing 100 mL liquid MS medium supplemented with 1.0 mg/L 2,4-dichlorophenoxyacetic acid (2,4-D), which lasted for 8 days at 25 ± 0.5 °C with shaker speed 120 rpm in the dark. It could derive rosemary suspension cell lines with high cell viability and stable growth after the suspension culture for several generations. The culture was performed by transferring 4 g·FW/20 mL of 6-day-old culture (cells plus medium) to 80 mL of the fresh growth medium, which lasted for 8 days. MeJA were sterilized by filtration through a 0.22 μm sterile syringe filter and added to the medium on day 6 of the culture process. The final concentrations of MeJA solution were 0, 10, 50, and 100 μM in the medium. Each test was repeated three times. After 48 h treatment, all materials were stored at −80 °C for later use. Samples treated with 0 versus 10 μM MeJA, 0 versus 50 μM MeJA, and 0 versus 100 μM MeJA, were named CKvsM10, CKvsM50, and CKvsM100, respectively. The culture was performed by transferring 4 g·FW/1 L of 6-day-old culture (cells plus medium) to 5 L of the fresh growth medium, which lasted for 14 days in 5 L stirred bioreactor at 25 ± 0.5 °C with a rotational speed of 100 rpm and a ventilation of 100 ccm in the dark. MeJA were sterilized and added to the medium on day 8 of the culture process. The final concentration of MeJA solution was 100 μM in the medium. After 144 h treatment, all materials were stored at −80 °C for later use.

### 4.2. Small RNA and RNA-Seq Library Construction

Total RNAs were isolated from rosemary suspension cells. The qualities and concentrations of RNA were detected using 1.0% agarose gel electrophoresis and a NanoDrop 2000 spectrophotometer (Thermo Scientific, Wilmington, DE, USA). The integrity and concentration of RNA samples were further checked using an Agilent 2100 Bioanalyzer. Small RNAs of different sizes were isolated from total RNAs by a polyacrylamide gel electrophoresis (PAGE) gel and ligated to a 3′ Illumina adapter. These adapter-ligated small RNAs were reverse-transcribed to cDNA with a reverse transcription (RT) primer using a Super-script II Reverse Transcriptase Kit (Invitrogen, Carlsbad, CA, USA) to generate small RNA (sRNA) libraries. Finally, small RNA libraries were sequenced using an Illumina HiSeq 4000 platform (Shenzhen, China).

### 4.3. General Analysis of Small RNA and Prediction of miRNA Targets

Clean reads were mapped onto the reference miRBase 20.0 using Anchor Alignment-Based Small RNA Annotation (AASRA) software [90]. We use miRNA (for plants) to predict novel miRNA by exploring the characteristic hairpin structure of miRNA precursor [91]. In order to find more accurate targets, multiple types of software are used. Generally, we use psRobot [92], TAPIR [93], or TargetFinder [94] to predict targets.

### 4.4. Identification of Differentially Expressed miRNAs

Using the findings of the Genome Res paper entitled the significance of digital gene expression profiles [95]. The *p*-value of the differential gene expression test is corrected using the Bonferroni method [96], the false discovery rate (FDR) was adjusted using qvalue. To judge the significance of gene expression difference, FDR ≤ 0.001 and the absolute value of Log2Ratio ≥ 1’ are set as the default threshold. The smaller the FDR value, the greater the difference multiple, which indicates there are significant differences in expression. In BGI’s experience, differentially expressed genes (DEGs) were defaulted as genes with FDR ≤ 0.001 and multiples of more than 2-fold.

Based on KEGG (the major public pathway-related database) annotation results and official classification, whole target genes of differentially expressed miRNAs (DEMs) were mapped to the KEGG database, and the number of genes in each pathway was calculated. The *p*-value is corrected by using the Bonferroni method [82], and a corrected *p*-value ≤ 0.05 is taken as a threshold. KEGG terms fulfilling this condition are defined as significantly enriched KEGG terms.

### 4.5. qRT-PCR Validation of miRNAs and Their Targets

Total RNAs from rosemary suspension cells were further used for quantitative polymerase chain reaction (qRT-PCR) validation of miRNAs and their target genes. Expression was validated using a LightCycler480 Real-time PCR system (Roche, Basel, Switzerland). Relative gene expression and miRNA expression levels were evaluated using the method described by Song using TransScript^®^ miRNA first strand cDNA synthesis supermix (Tran, Fuzhou, China) and RevertAid First Strand cDNA Synthesis Kit (Takara, Shanghai, China). Relative expression levels were determined using the comparative 2^−ΔΔCt^ method. Primer sequences were designed using DNAMAN V6.0 and are listed in Table S1. All treatments were analyzed using biological triplicates.

### 4.6. Determination of Flavonoid, Rosmarinic Acid, and Carnosic Acid

Flavonoid contents were determined using Li’s method with some modifications [49]. Rosemary suspension cells were freeze-dried for 3 days in a refrigerant dryer (LGJ-25C, Sihuan, Beijing, China), ground into fine powders in 0.5 g measures, and extracted with 10 mL 60% ethanol and then ultrasonicated for 1 h at 60 °C. The extracts were centrifuged at 8000 rpm for 5 min at 20 °C in duplictae. The supernatant was collected into new tubes and constant volume to 20 mL. The flavonoid contents were detected at a wavelength of 510 nm in the DU640 spectrophotometer using a chromogenic reaction method.

Rosemary suspension cells were freeze-dried for 3 days in a refrigerant dryer, ground into fine powders 0.5 g, and extracted with 10 mL if 60% ethanol, then ultrasonicated for 45 min at 40 °C, the extracts were centrifuged at 8000 rpm for 10 min at 20 °C in duplicates. The supernatant was collected into new tubes and constant volume to 20 mL. Finally, the supernatant of the extract was separated and filtered through a 0.22 μm aqueous filter membrane.

The following chromatographic conditions were used for rosmarinic acid determination. Analysis was performed on a Diamonsil C18 (Beijing, China) (4.6 × 200 mm, 5 μm) column with isocratic elution using a mobile phase of 0.1% aqueous formic acid and acetonitrile (45%:55%). The column temperature and flow rate were set at 30 and 1.0 mL/min, respectively. All standards and samples were detected by UPLC (Waters, Beverley, MA, USA) at a wavelength of 330 nm. Rosmarinic acid standards were HPLC ≥ 98% (Solarbio, Shanghai, China).

The following chromatographic conditions were used for carnosic acid determination. Analysis was performed on a Diamonsil C18 (Beijing, China) (4.6 × 200 mm, 5 μm) column with isocratic elution using a mobile phase of 0.1% aqueous formic acid and acetonitrile (15%:85%). The column temperature and flow rate were set at 30 and 1.0 mL/min, respectively. All standards and samples were detected by UPLC (Waters, Beverley, MA, USA) at a wavelength of 230 nm. Carnosic acid standards were HPLC ≥ 98% (Solarbio, Shanghai, China).

### 4.7. Determination of Physiological and Biochemical Indexes

Briefly, 0.1 g of rosemary suspension cells (fresh weight) from each of the treatment group was rapidly frozen with liquid nitrogen. Samples were maintained at 2–8 °C after melting, to which we added 1 mL PBS (PH7.4). The resultant solution was homogenized by hand and then subjected to centrifugation for 20 min at 2000–3000 rpm. Then, the supernatant was removed. Auxin (IAA), abscisic acid (ABA), gibberellin (GA), JA, and salicylic acid (SA) were assayed using ELISA Kit (Weilan, Shanghai, China) and a Microplate Reader (Rayto RT-6100) according to the manufacturer’s instructions.

Determination of amino acid: 0.2 g of rosemary suspension cells (fresh weight) from each of the treatment groups were added to 1 mL H_2_O_2_, ground into slurry and transfered it into EP tubes. The solution was then extracted overnight via a filter membrane.

Derivation of amino acids: 200 μL of the above clear solution to be derived and 200 μL of amino acid standard solution were combined in a 1.5 mL EP tube respectively; to which we added 20 μL of norleucine internal standard solution to each centrifuge tube. Then, 200 μL of triethylamine acetonitrile solution (ensure pH > 7) and 100 μL of phenyl isothiocyanate acetonitrile solution were added respectively. After mixing, the solution was stored at 25 °C for 1 h. Then, 400 μL of n-hexane was added to the centrifuge tube, shaken, and left to rest for 10 min. The resultant lower solution was then passed through a 0.45 μM needle filter. 

HPLC liquid phase conditions: UPLC (RIGOL L3000, Beijing, China), Sepax C18 (250 × 4.6 mm, 5 μm, Beijing, China) Mobile phase A: 7.6 g C_2_H_3_NaO_2_ was added to 925 mL H_2_O_2_, dissolved, and then the PH was adjusted to 6.5 with glacial acetic acid, then 70 mL of acetonitrile was added, mixed well, and the solution was filtered through a 0.45 μm filter membrane. Mobile phase B: 80% acetonitrile aqueous solution; gradient elution (Figure S4). The injection volume was set to 10 μL. The flow rate was 1.0 mL/min, the column temperature was 40 °C, and the sampling time was 45 min. Amino Acids Mixture Standard Solution Standards, (Sigma-Aldrich, Shanghai, China).

Determination of linoleic acid and alpha-linolenic acid: samples were dried at 80 °C, 0.1 g sample (dry weight), ground and placed in a 15 mL centrifuge tube. The mortar was washed with 10 mL n-hexane three times and the samples were transferred to 15 mL centrifuge tubes. Then samples were ultrasonicated at 50 °C for 30 min. Next they were centrifuged at 8000 r/min for 10 min. The supernatant was then placed into 15 mL centrifuge tubes. Then 5 mL n-hexane was added to the residue. The above extraction steps where repeated in duplicate, and the resultant supernatant was combined. An appropriate amount of Na_2_SO_4_ was added into the supernatant, shaken and centrifuged. The supernatant was transferred to 2 mL EP tubes and blown with nitrogen until the solvent volatilized completely. At this point, 0.8 mL reagent III was added into the EP tube, shaken, and dissolved, and withheld from light reaction for 1 h. Then, 0.8 mL n-hexane, was added shaken, and mixed well for 1 min, left to stand for layering, and the upper n-hexane was transferred to 2 mL EP tubes, extracted 3 times, and combined the n-hexane phase. After the nitrogen is blown dry, 1 mL of n-hexane was added, shaken, and dissolved. An appropriate amount of the solution was then taken and filtered with a needle filter into a sample bottle with an inner liner to be tested. 

GC-MS conditions: The pressure valve of nitrogen cylinder was opened and the pressure adjusted to 0.4 MPa. The computer was activated and the gas phase workstation was initiated, setting the column box temperature at 200 °C, the front detector temperature to 250 °C, and the rear sample inlet temperature at 220 °C, the method was then saved and issued. After the ignition column temperature rose to 100 °C, it was monitored. Samples were added after the baseline was stable. Linoleic acid and alpha-linolenic acid Standards, GC ≥ 98% (Yuanye, Shanghai, China).

### 4.8. Statistical Analysis

Quantitative results for rosemary metabolite content, enzyme activity, and gene expression analyses are presented as the means ± standard deviations (SDs) of at least three biological replicates. The effects of MeJA conditions on metabolite contents and gene expression were analyzed by one-way analysis of variance (ANOVA) followed by Duncan’s test using SPSS version 19.0. Different upper/lower case letters indicate statistically significant differences at 0.01 levels. Figures were prepared using GraphPad Prism 8.0 and Excel 2016 software.

## Figures and Tables

**Table 1:** miRNAs and target genes related to plant hormone signal transduction in rosemary suspension cells.

Combination	Target Genes	miRNAs	Target Finder Score	Corresponding Hormone
CKvsM50 CKvsM100	CL11742.Contig5_All	miR160	1	Auxin (*ARF*)
	CL7275.Contig1_All	miR160a-5p	1	
	CL7275.Contig2_All	miR160a-5p	1	
	CL8053.Contig1_All	miR160a-5p	0.5	
	CL8053.Contig2_All	miR160a-5p	0.5	
	CL8053.Contig3_All	miR160a-5p	0.5	
	CL11742.Contig1_All	miR160a-5p	1	
	CL11742.Contig3_All	miR160a-5p	1	
	CL11742.Contig4_All	miR160a-5p	1	
CKvsM10	CL4553.Contig1_All	miR156a-5p	1	Gibberellin (*DELLA*)
CKvsM50 CKvsM100	CL3157.Contig1_All CL3157.Contig4_All	miR171b-3p	2	
	CL3157.Contig1_All CL3157.Contig4_All	miR171b-3p_3	3	
	CL3157.Contig1_All CL3157.Contig4_All	miR171d_1	1	
	CL3157.Contig1_All CL3157.Contig4_All	miR171f_3	1	
CKvsM50 CKvsM100	CL1203.Contig7_All	miR171b-3p	3.5	Ethylene (*ETR*)
CKvsM10	CL9049.Contig3_All	miR156a	2	Brassinosteroid (*BRI1*)
	CL9049.Contig3_All	miR156a-5p	1	

## Data Availability

Not applicable.

## Supplementary Materials

The following supporting information can be downloaded at: https://www.mdpi.com/article/10.3390/ijms23073704/s1.

## Abbreviations

MeJA	methyl jasmonate
IAA	indoleacetic acid
ABA	abscisic acid
GA	gibberellin
SA	salicylic acid
JA	jasmonate
BR	brassinosteroids
ET	ethylene
2,4-D	2,4-dichlorophenoxyacetic acid
qRT-PCR	quantitative real-time PCR
DEMS	differentially expressed miRNAs
RA	rosmarinic acid
CA	carnosic acid
rpm	revolutions per minute
ccm	cubic centimeters per minute
