# Supplementary material for: Exploring the Effect of Methyl Jasmonate on the Expression of microRNAs Involved in Biosynthesis of Active Compounds of Rosemary Cell Suspension Cultures through RNA-Sequencing
Source: Int J Mol Sci. 2022 Mar 28;23(7):3704. doi: 10.3390/ijms23073704 (PMC8998883; doi:10.3390/ijms23073704)
Supplement: Supplementary file 1 [file ijms-23-03704-s001.zip › ijms-1558715-supplementary.pdf]

Supplementary Material

Exploring the Effect of Methyl Jasmonate on the Expression of microRNAs Involved in Biosynthesis of Active Compounds of Rosemary Cell Suspension Cultures through RNA-Sequencing

Deheng Yao[1, 2], Yukun Chen [1], Xiaoping Xu [1], Yuling Lin [1], and Zhongxiong Lai [1,*]

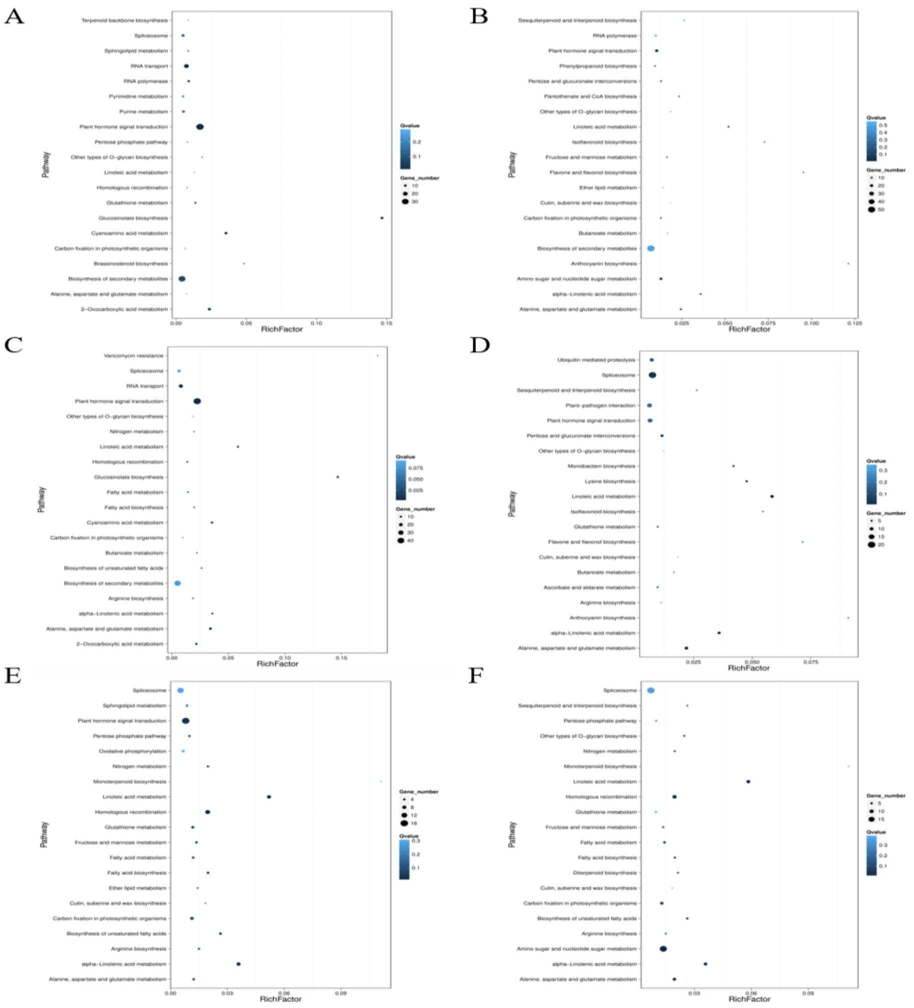

Figure S1. Top 20 enriched and regulated KEGG pathways. A, CKvsM10, B, CKvsM50, C, CKvsM100, D, M10vsM50, E, M10vsM100, F, M50vsM100.

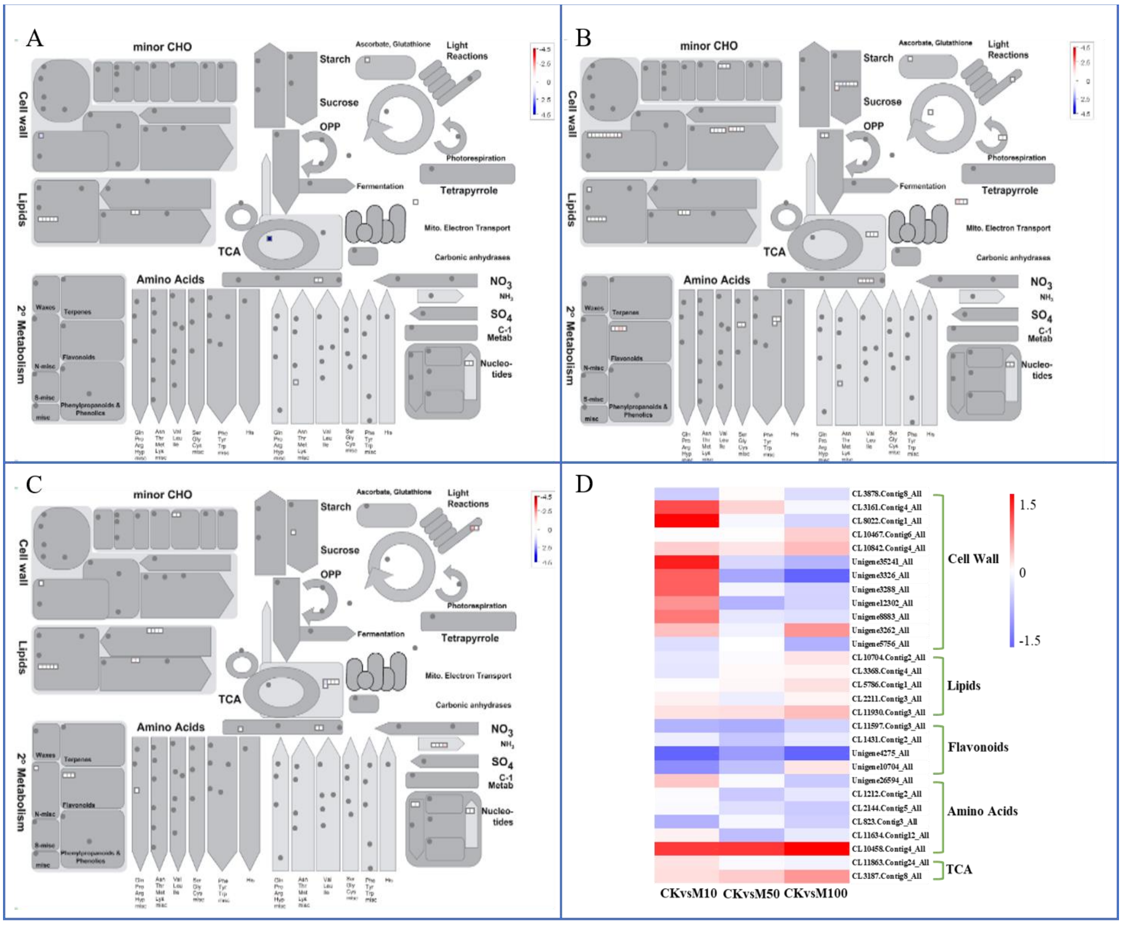

Figure S2. Metabolic pathways in rosemary suspension cells under different concentrations of MeJA. A, Metabolic pathways of differentially target genes in the CKvsM10; B, Metabolic pathways of differentially target genes in the CKvsM50; C, Metabolic pathways of differentially target genes in the CKvsM100; D, Heat maps of expression of differentially target genes under different concentrations of MeJA. A, B, and C： From the red to the blue corresponds to the numerical value of log2(Relative expression) from the low to the high. D: From the red to the blue corresponds to the numerical value of log2(Relative expression) from the high to the low

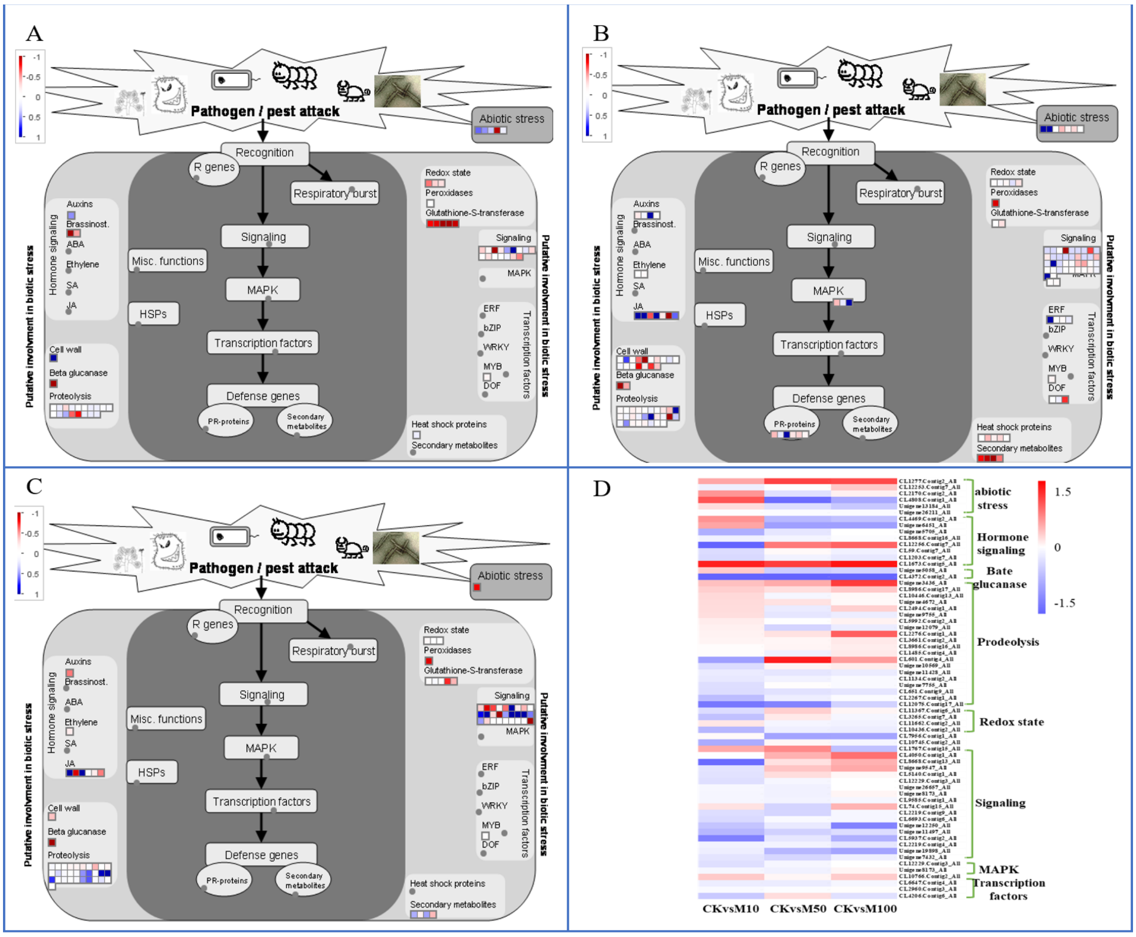

Figure S3. Abiotic stress in rosemary suspension cells under different concentrations of MeJA. A, Abiotic stress of differentially target genes in the CKvsM10; B, Abiotic stress of differentially target genes in the CKvsM50; C, Abiotic stress of differentially target genes in the CKvsM100; D, Heat maps of expression of differentially target genes under different concentrations of MeJA. A, B, and C： From the red to the blue corresponds to the numerical value of log2(Relative expression) from the low to the high. D: From the red to the blue corresponds to the numerical value of log2(Relative expression) from the high to the low

mobile phase gradient elution		
**Time（min）**	**mobile phase A(%)**	**mobile phase B(%)**
**0**	**100**	**0**
**2**	**100**	**0**
**15**	**90**	**10**
**25**	**70**	**30**
**33**	**55**	**45**
**33.1**	**0**	**100**
**38**	**0**	**100**
**38.1**	**100**	**0**
**45**	**100**	**0**

Figure S4. HPLC liquid phase conditions for amino acids determination

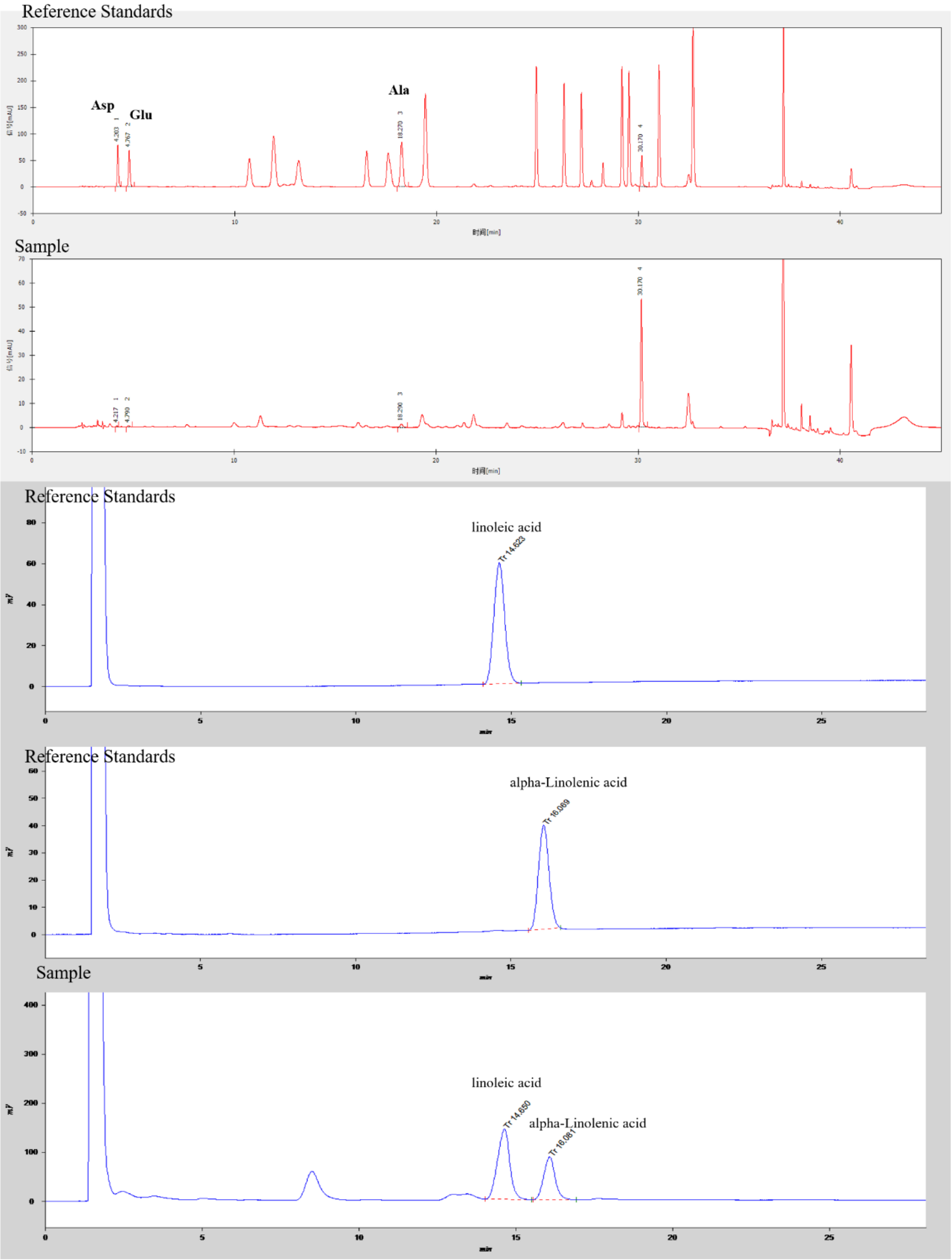

Figure S5. Chromatograms with standard compounds and sample

Table S1. Primers information used for real-time PCR analysis of rosemary genes

Gene name	Primer sequences（5'-3'）
miR156a	TGACAGAAGAGAGTGAGCACA
miR156a-5p	TGACAGAAGAGAGTGAGCAC
miR160a-5p	TGCCTGGCTCCCTGTATGCCA
miR167d_1	TGAAGCTGCCAGCATGATCT
miR171b-3p	TTGAGCCGTGCCAATATCAC
miR171b-3p_3	TTGAGCCGTGCCAATATCACG
miR171d_1	TTGAGCCGCGCCAATATCAC
miR171f_3	TTGAGCCGCGCCAATATCACT
miR396a-3p_5	TTCAATAAAGCTGTGGGAAG
miR399b	TGCCAAAGGAGAGTTGCCCTG
miR6300	GTCGTTGTAGTATAGTGG
CL8954.Contig1_All-F	TTCACCTGAATAGACCACACCG
CL8954.Contig1_All-R	CTTCTGATTGCTCCTCTGTGGT
CL11742.Contig1_All-F	ACGGTTTCGACGACAATGGA
CL11742.Contig1_All-R	TGCCGGAACTTCCAAGTCTC
CL9049.Contig3_All-F	TCACCATCAGCAGCATCAACT
CL9049.Contig3_All-R	GGAGGCGATGACAGAAGAGAG
CL1264.Contig2_All-F	AGCACAGACCTTTGCATACGA
CL1264.Contig2_All-R	CACGTTTGTCTGTGCTGTGAG
CL3157.Contig1_All-F	GACTGGGAGAGCGTGCTATC
CL3157.Contig1_All-R	ATTTGGGAGCGTGGGCATAA
CL10446.Contig22_All-F	GCCCAACTGAAGGAGCAGAT
CL10446.Contig22_All-R	TTCTCATGCCAGCCGTTGAA
CL5140.Contig4_All-F	TCCTGGCTATGGAGTGAGCT
CL5140.Contig4_All-R	ATCCTTTTCTCGCTCACGCA
CL11662.Contig2_All-F	CTGGCATCGTTGAGGACCTT
CL11662.Contig2_All-R	TGCGCTCCAACCGTAGAATT
CL2063.Contig1_All-F	CCCAACGTTGATCCACCTGA
CL2063.Contig1_All-R	TTTCTCCGGTTCGTGGACTG

Table S2. Rosmarinic acid contents of rosemary suspension cells under different MeJA concentrations

MeJA concentrations (μM)	Treatment time (h)	rosmarinic acid contents 1 (mg•g $^{-1}$DW)	rosmarinic acid contents 2 (mg•g $^{-1}$ DW)	rosmarinic acid contents 3 (mg•g $^{-1}$ DW)	Average rosmarinic acid contents (mg•g $^{-1}$ DW)	STDEV	Duncan (5%)
0	48	1.971	2.001	2.046	2.006	0.038	d
10<br>50<br>100	48	2.158	2.223	2.199	2.194	0.033	c
	48	2.954	2.798	3.043	2.932	0.124	b
	48	3.288	3.159	3.428	3.292	0.134	a
	48	2.746	2.695	2.542	2.661	0.106	a

Table S3. Carnosic acid contents of rosemary suspension cells under different MeJA concentrations

MeJA concentrations (μM)	Treatment time (h)	Carnosic acid contents 1 (mg•g $^{-1}$DW)	Carnosic acid contents 2 (mg•g $^{-1}$ DW)	Carnosic acid contents 3 (mg•g $^{-1}$ DW)	Average Carnosic acid contents (mg•g $^{-1}$ DW)	STDEV	Duncan (5%)
0	48	1.501	1.537	1.468	1.502	0.034	d
10	48	1.642	1.669	1.681	1.664	0.020	c
50	48	2.115	2.228	2.183	2.175	0.057	b
100	48	2.746	2.695	2.542	2.661	0.106	a

Table S4. Flavonoids contents of rosemary suspension cells under different MeJA concentrations

MeJA concentrations (μM)	Treatment time (h)	Flavonoids contents 1 (mg•g $^{-1}$DW)	Flavonoids contents 2 (mg•g $^{-1}$ DW)	Flavonoids contents 3 (mg•g $^{-1}$ DW)	Average Flavonoids contents (mg•g $^{-1}$ DW)	STDEV	Duncan (5%)
0	48	14.891	15.608	14.433	14.977	0.592	d
10	48	15.607	17.219	17.060	16.629	0.888	c
50	48	19.050	18.413	18.573	18.679	0.331	b
100	48	20.522	20.383	21.578	20.828	0.653	a

Table S5. GA contents of rosemary suspension cells under different MeJA concentrations

MeJA concentrations (μM)	Treatment time (h)	GA contents 1 (pmol/g FW)	GA contents 2 (pmol/g FW)	GA contents 3 (pmol/g FW)	Average GA contents (pmol/g FW)	STDEV	Duncan (5%)
0	48	0.389	0.457	0.620	0.489	0.119	c
10	48	0.636	0.756	0.688	0.694	0.060	bc
50	48	0.875	0.702	0.843	0.807	0.092	ab
100	48	0.776	0.765	0.792	0.777	0.014	a

Table S6. ABA contents of rosemary suspension cells under different MeJA concentrations

MeJA concentrations (μM)	Treatment time (h)	ABA contents 1 (ng/g FW)	ABA contents 2 (ng/g FW)	ABA contents 3 (ng/g FW)	Average ABA contents (ng/g FW)	STDEV	Duncan (5%)
0	48	1090.825	1278.482	1187.879	1185.729	93.847	a
10	48	1044.542	1038.090	1102.606	1061.746	35.532	ab
50	48	962.915	1120.559	996.856	1026.776	82.972	ab
100	48	978.062	905.972	1091.106	991.7132	93.319	ab

Table S7. IAA contents of rosemary suspension cells under different MeJA concentrations

MeJA concentrations (μM)	Treatment time (h)	IAA contents 1 (μmol/g FW)	IAA contents 2 (μmol/g FW)	IAA contents 3 (μmol/g FW)	Average IAA contents (μmol/g FW)	STDEV	Duncan (5%)
0	48	0.155	0.1185	0.120	0.131	0.021	b
10	48	0.179	0.1467	0.153	0.159	0.018	ab
50	48	0.172	0.144	0.192	0.169	0.024	ab
100	48	0.1645	0.1893	0.218	0.191	0.027	a

Table S8. JA contents of rosemary suspension cells under different MeJA concentrations

MeJA concentrations (μM)	Treatment time (h)	JA contents 1 (pmol/g FW)	JA contents 2 (pmol/g FW)	JA contents 3 (pmol/g FW)	Average JA contents (pmol/g FW)	STDEV	Duncan (5%)
0	48	12.214	16.043	11.391	13.216	2.483	b
10	48	13.782	16.941	14.413	15.045	1.671	ab
50	48	22.061	16.895	19.798	19.58	2.589	a
100	48	21.424	17.089	16.901	18.472	2.559	a

Table S9. SA contents of rosemary suspension cells under different MeJA concentrations

MeJA concentrations (μM)	Treatment time (h)	SA contents 1 (μg/g FW)	SA contents 2 (μg/g FW)	SA contents 3 (μg/g FW)	Average SA contents (μg/g FW)	STDEV	Duncan (5%)
0	48	73.763	56.634	69.697	66.698	8.949	a
10	48	61.880	68.749	55.349	61.992	6.701	a
50	48	45.024	43.867	45.910	44.934	1.025	b
100	48	35.102	44.478	53.727	44.435	9.312	b

Table S10. Alanine contents of rosemary suspension cells under different MeJA concentrations

MeJA concentrations (μM)	Treatment time (h)	Alanine contents 1 (μg/g FW)	Alanine contents 2 (μg/g FW)	Alanine contents 3 (μg/g FW)	Average Alanine contents (μg/g FW)	STDEV	Duncan (5%)
0	48	19.634	20.024	21.123	20.260	0.772	c
10	48	10.635	10.212	9.700	10.182	0.468	d
50	48	28.577	28.175	29.504	28.752	0.681	b
100	48	33.413	33.037	34.014	33.488	0.493	a

Table S11. Glutamate contents of rosemary suspension cells under different MeJA concentrations

MeJA concentrations (μM)	Treatment time (h)	Glutamate contents 1 (μg/g FW)	Glutamate contents 2 (μg/g FW)	Glutamate contents 3 (μg/g FW)	Average Glutamate contents (μg/g FW)	STDEV	Duncan (5%)
0	48	21.251	20.373	19.293	20.306	0.981	c
10	48	18.123	17.471	17.334	17.643	0.422	d
50	48	22.146	21.539	22.529	22.071	0.499	b
100	48	44.515	43.054	43.092	43.554	0.833	a

Table S12. Aspartate contents of rosemary suspension cells under different MeJA concentrations

MeJA concentrations (μM)	Treatment time (h)	Aspartate contents 1 (μg/g FW)	Aspartate contents 2 (μg/g FW)	Aspartate contents 3 (μg/g FW)	Average Aspartate contents (μg/g FW)	STDEV	Duncan (5%)
0	48	10.584	10.806	11.634	11.008	0.554	a
10	48	5.637	5.899	6.026	5.854	0.198	c
50	48	5.487	5.275	5.389	5.384	0.106	d
100	48	10.581	10.812	10.628	10.674	0.122	ab

Table S13. linoleic acid contents of rosemary suspension cells under different MeJA concentrations

MeJA concentrations (μM)	Treatment time (h)	linoleic acid contents 1 (mg/g DW)	linoleic acid contents 2 (mg/g DW)	linoleic acid contents 3 (mg/g DW)	Average linoleic acid contents (mg/g DW)	STDEV	Duncan (5%)
0	48	5.456	5.464	5.481	5.467	0.013	b
10	48	4.514	4.593	4.668	4.591	0.077	d
50	48	25.483	25.684	25.870	25.679	0.193	a
100	48	5.353	5.385	5.414	5.384	0.030	c

Table S14. alpha-Linolenic acid contents of rosemary suspension cells under different MeJA concentrations

MeJA concentrations (μM)	Treatment time (h)	alpha-Linolenic acid contents 1 (mg/g DW)	alpha-Linolenic acid contents 2 (mg/g DW)	alpha-Linolenic acid contents 3 (mg/g DW)	Average alpha-Linolenic acid contents (mg/g DW)	STDEV	Duncan (5%)
0	48	3.525	3.515	3.413	3.484	0.062	c
10	48	3.370	3.569	3.450	3.463	0.100	cd
50	48	21.809	22.043	22.160	22.004	0.179	a
100	48	4.504	4.584	4.646	4.578	0.071	b

Table S15. Summary statistics of small RNA libraries.

Library	Raw reads	Clean Reads	Percentage of clean (%)	Mapped rsads	Percentage (%)
CK	35,202,242	30,808,982	87.52	16,669,449	54.11
M10	34,995,702	31,400,940	89.73	17,162,631	54.66
M50	32,667,184	19,653,455	86.74	11,083,865	58.86
M100	32,667,184	29,666,471	90.81	17,462,009	56.40

Table S16. Summary of detected miRNAs for each sample.

Sample name	CK	M10	M50	M100
Total Known miRNA count	69			
Total novel miRNA count	181			
Known miRNA count	56	53	57	53
Known miRNA precursor count	45	53	46	51
Novel miRNA count	178	178	178	172
Novel siRNA count	16683	15765	16570	13434

Table S17 The information of mature novel miRNAs and their precursor

miRNA id	Sequence(mature)	Sequence(precursor)
novel_mir1	CAAAAAGGAAAGTAGGACATTTGTA	AAGTACTCCCTCCGTCCCATTTAAACTGTCCCCTTTCTTTTGGGCACGGGTATTTAGGAGATAAGTATAAAGTAGTTAAAGTGATAAAAAGTTGGTAGGGTCCACACTTTTTGAAGGGATAATTACTTCCTAAAATGGAATGAGACATTTGTAATGGGACAACCCAAAAAGGAAAGTAGGACATTTGTAATGGGACAGAGGGAGTATTA
novel_mir2	ACGTTTTAAGTGGGACGGAGGGAG	TTATTCCCTCTGTCCCATTTAAAATGTCCTACTTTCCTTTTTGGGTTGTCCCATTACAAATGTCTCATTCCATTTTAGGAAGTAATTATCCCTTCAAAAAATGTGGACCCTACCTACTTTTTAACACTTTAACTACTTTATACCTTTCTCCTAAATAACCGTGTCCAAAAGAAAGGGGACGTTTTAAGTGGGACGGAGGGAGTAT
novel_mir3	ATGGGATTAAGGTAGTGCATTATAA	ATTATATTTATGGTATATGCATAGATCCTTTCCATATAAGATTATGGTAATAATCCTGAGAGTATGGTAATAATCCTAGGAGTATGGTAATTTCCATATAGGATTATGGTAATATTCCATATAGGATTATGGTAATTTCCATATGGGATTAAGGTAGTGCATTATAAATATAAG
novel_mir4	CAAAAGAAATAGGACACTT	ATACTCCATCTGTCCCATTTAAAATGTCCTACTTTTCTTTTTGGGTTGTCCTATTACAAATGTCTCATTCCATTTTAGGAAATAATTATCCCTTCAAAAAGTGTGGGTCCTACTCAATTTTAACACTTTAGCTATTTTATACCTTTCTCCTAAATACTCGTGCCCAAAAGAAATAGGACACTTTAGGCGGGACGGAGGGAGTAC
novel_mir5	CGGTGGTAGAGTTATTGATCCA	TCCAATAACCACCGTAGCTTCGGAGTCTGCATTTAGTGCCGGTGGTAGAGTTATTGATCCACATCGAGCCTCTCTAGCAGTAGACACGGTTCAAATGCTACTTTGTGCGGGAGATTGGT

novel_mir6	ACACTTTTTGAAGGGATAATTA	CGTCCCATTTAAAGTGTCTCTTTTATTTTGGGCACGGGTATTTAGGAGATATGTATAAAGTAGTTAAAGTGATAAAAAGTGGGTAGGGCCCACACTTTTTGAAGGGATAATTACTTCCTAAAATGGAATGAGACATTTGTAATGGGACA
novel_mir7	ATAACATAAAATCGATCGATCAGTTTT	TGAATGAAATAACATAAAATCGATCGATCAGTTTTACGCTAATCTCGACCGGTATTATGCTAATTTTGTTGGTTTTATCTATTTTACGTAAATCCAATTGAATTAGCATAAAACTGATCGATCGGCTTTATGTTATTTCATTTT
novel_mir8	TTAATGTTAATAAAGTAGGAGAGAGAAATA	ATAATACTCCCTCCGTCCCGGCCCAATTGGCACAACACTTTTTGCACAAATATTAAGAAAAAATGGGTTAAGGGGTTTAATGGTGTTGGTCCCACAAGATTTAATGTTAATAAAGTAGGAGAGAGAAATAGAATTGGAGAGAGAAAGAGAGAGTAGAATATAGAGAGACATGATTGGAGAGAGACATAGTAGATTAAGGGATTTAATTTTTGCCATTTTTGGCATGTGCCAATTGGATTGGGACAGCCCAATTTGGCAAGTGTGCCAATTGGGCTGGGACGGAGGGAGTATTAC
novel_mir9	AGGGGCGACATGAGAACATAT	TGAAATCCAGCAGGGGCGACATGAGAACATATAATTGATATGTCGTCATCTTATATGTTCTCAGGTCGCCCCTGTAGGATTTCT
novel_mir10	ATTGAGTGCAGCGTTGATGACATCGTCAT	GGTTAAAAACCTCATTGAGTGCAGCGTTGATGACATCGTCATTCGTCCTTCTCCTTCGTCTCCGGATGTCGTTTCATTGATGCTGCACTCAATGACGTTTTTAATTT
novel_mir11	AATACATGTCATCTTGGGTGGAACTGAA	CTTTTTCAGTTCATCTTGGATGGGACAACCCAAAAAGGAATACATGTCATCTTGGGTGGAACTGAAGAAC
novel_mir12	CCGGAACCGTTGTTTTAAAATTTAATG	GTGGATATGTAATGCTCAATTTTAAAAAAAAAAAAAAAAAAAAAAAAACTGACCGGGTCGGACCGGAACCGGACAAAACGGGTCGGGTCTTGACCGGTCCGGGTCCAAGCGGTTTTGGAACCGGAACCGAAACCGGAACCGTTGTTTTAAAATTTAATGGGTTTTGAACGGTTCCGGATCGGTCCGGGTTAAGACCGCCCGTTGACATCCCTAGTTACAACAATTCAA

novel_mir13	TTGGAGGTCCTCGGTCAGGTCATCCCGGGG	CGCCGGGCCCTGGGTGCCGACCTGGATGACCCTCGAGGGATCGTCGTCGTATAGTTGGAGGTCCTCGGTCAGGTCATCCCGGGGTGTCGGCCTCCGGGGGCTCGGTA
novel_mir14	GAATATGCTATGAAAGTAAATGAATA	AATATATATATATAGGGGAGGGTTAACGTAAAAACCATTTTTATTGTGCGAACGTGAGAACTGAATATGCTATGAAAGTAAATGAATACACTGCGAAAGTTAATGAATAAAAAAAAATAGAATTTTCGCCTCTCATGGGATTCGAACCCTGGTGAAAATTCCGAATTTTTGGATTCATTAACTTTCGCAGAGTATTCATTAACTTTCATAGCATATTCATTTTGTTTTCGTAGTTCTTAGTTCTCACGTTAAAATTGGTTCTTATTTGAACCAAACTCTATATATATATATA
novel_mir15	GTAGTTAAAGTGTTAAAAAGTGGG	ATACTCCCTCCGTCCCACCTAAATGTCCCATTTCTTTTGGGCACGGGTATTTAGGAGAAAGGTATAAAGTAGTTAAAGTGTTAAAAAGTGGGTAGGGTCCACACTTTTTGAAGGGATAATTATTTCCTAAAATGGAATGGGACATTTGTAATGGGACAACCCAAAAAGGAAAGTATGACATTTTAAATGGGACAGAGGGAGTAA
novel_mir16	ACCCATCCATTGACGAGCCAAAGT	TAGTTGATGGTCCTGGTCCGGTCTGGATTCAATGTCTAATGGATCGGATCCAGAACCAGAACCATTACTTATTTATTGGTTCTGGTTCGGGTAAAACCCATCCATTGACGAGCCAAAGTTTAAATAGGCTTGTCAATGAGCTAGTTTTATCCATATCAATTC
novel_mir17	AAATGGAATGAGACATTCATAATGGGACAA	ATTACTCCCTCCGTCCCATTTAATGTGTCTCATTTCTTTTAGGCACGGGTAATTAGGAGAAGGGTATAAAGCAATTAAAGGAGTAAAAAGTGGGTATGGCCCATACTTTTTGAAGGAATAATTATTTCTTAAAATGGAATGAGACATTCATAATGGGACAACCAAAAAAGGAAAGTATGACATTTTAAATGGGATGGAGTAAG

novel_mir18	CTGTTTTGTGCATTATCTTCAACGTCTTAT	GTGAGAACTAAGAACCATGCATAAGACGTTGAAGATGATGCACAAGATGATATAAAGTTGTGCATAACGAAAACTTTATAAAATTATTTTGTTGGGCAACAATTTTAGAAAGTTTTCGTTATGCACAACTTTATACTGTTTTGTGCATTATCTTCAACGTCTTATGCATGGTTCTCAAATTCTCAA
novel_mir19	TTTCATAGCATATTCATTTTGTTCGTAA	CGTGAGAACCATTTTTATTGTGAGAACATGAGAAATGAATATGCTATGAAAGTTAATGAATACGCTGCGAAAGTTAATGAATAAAAAAAAAATAGAATTTTCGCCTCTCATGGGATTCGAACCCTGGTGAAAATTCCGAATTTTTGGATTCATTAACTTTCGCAGAGTATTCATTAACTTTCATAGCATATTCATTTTGTTCGTAAAGAATTTTCGTAGTTCTTAGTTCTCACGTTAAAATTGGTTCTTATT
novel_mir20	TTAATGAATACTCTGCGAAAG	TTTAACGTGAGAACTAAGAACTACGAAAATTCTTTACGAACAAAATGAATATGCTATGAAAGTTAATGAATACTCTGCGAAAGTTAATGAATCCAAAAATTCGGAATTTTCACCAGGGTTCGAATCCCATGAGAGGCGAAAATTCTATTTTTTTTTATTCATTAACTTTCGCAGCGTATTCATTAACTTTCATAGCATATTCATTTCTCATGTTCTCACAATAAAAATGGTTCTCACGTTAAC
novel_mir21	TGAGAACCATGCATAAGACGTTGAAGATA	ACTACATACTTCTCAATTCTTAGAACTTATCTGCGGGCTTGTTTTGTATTCGAATTTGTATTCTTCGAATGCATGTTAGTCTAGATGAAAGGATAAAACCTACTTAAATGCAATATTGCAGTTGTTTGGAGATGAGTTTGTTAATTATATATATATATATAGGGGTTGGATCCAGTAAGAACTCATCTTTTTTGAGAATTTGAGAACCATGCATAAGACGTTGAAGATAATGCACAAAACAGTATAAAGTTGTGCATAACGAAAACTTTATAAAATTATTTTGTTGGGCAACAATTTTAGAATGTTTTCGTTATGCACAACTTTATACTGTTTTGTGCATTATCTTCAACGTCTTATGCATGGTTCTCAAATTCTCAAAAAAGATGAGTTCTTACTGGATCCAACCCCTATATATATATATATAATTAACAAACTCATCTCCAAACAACTGCAATATTGCATTTAAGTAGGTTTTATCCTTTCATCTAGACTAACATGCATTCGAAGAATACAAATTCGAATACAAAACAAGCCCGCAGATAAGTTCTAAGAATTGAGAAGTATGTAGTT

novel_mir22	CAGTGGCGGAGCCACGTTGGGGCCT	TACAGACAGTGGCGGAGCCACGTTGGGGCCTGGGGGGCCCTTGGGCCCCCCAGAATTTTTTATTATTAGTAGTATATATTCAGTAAATAGACAAAGACTTCAATGTAGTTCAGTTGGTTTTGTT
novel_mir23	CGCCGAGGACTACCCAGATCTGGACA	GGCCGAGGCGGCGAGGGCCGAGGTTGCCTCGATGAAGGCTGAGGTGGAACAGGTTCGCCGTCGGGAGGCGGACATGGCGAGCTCTCAGGGAGAAACGCTTCGCGCCTTCGCGGAGAGGCTGTTCCTGTCGGCGGGCTTCTCGGGGATGGTCTCGGACGCGGCTGATGTGCTTAACAAGCGCGCCGTCTCGGACGCAATCGAAGAGGTCGCCGAGGACTACCCAGATCTGGACAAAGCCAAGTATGGGTACGAGGAGACCGAGGATGCTGTCCTGAGACGGGCCTACTTTGATGTGCTCGTCCGCGGCCTGAAGGAGCTTCCCGTGCTGCAGAACCTTGCCTCCTCCTCGGTT
novel_mir24	TCGCCGAGGACTACCCAGACCT	GAGGAGGCGGCAAGGGCCGAGGCGGCGAGGGCCGAGGTTGCCTCGATGAAGGCTGAGGTGGAACAGGTTCGCCGTCGGGAGGCGGACATGGCGAGCTCTCAGGGAGAAACGCTTCGCGCCTTCGCGGAGAGGCTGTTCCTGTCGGCGGGCTTCTCGGGGATGGTCTCGGACGCGGCTGATGTGCTTAACAAGCGCGCCGTCTCGGACGCAATCGAAGAGGTCGCCGAGGACTACCCAGACCTGGACAAAGCCAAGTATGGGTACGAGGAGACCGAGGATGATGTCCTGAGACGGGCCTATTTTGATGTGCTCGTCCGCGGCCTGAAGGAGCTTCCCGTGCTGCAGAACCTTGCCTCCTCCTCG
novel_mir25	ATTTTAAAGCAACGGTTCCGGTT	GGGGTTGTCTCTAGTAGGGATGTCAACGGGCGGTCTTTACCCGGATCGATCCGGAACCGTTCAAAGCCCATTAAATTTTAAAGCAACGGTTCCGGTTCTGGTTCTGGTTCTAGAACCGCTTGGACCCGGACCAGTTCAGACCCGGCCCATTTTGTCTGGTTCCGGTCCGACCCG

novel_mir26	CCGATTGTCACACGTTGAGCCG	CCCACGATGAGCGCCGATTGTCACACGTTGAGCCGCGTCGAGCATCGAGCGTCGAGCGCTGAAAGATGCCGAGCGTCGAGTGCCGAGCGGGCTTGCGGATGTGTGGGTGTGCGAGCAAAGGTGGC
novel_mir27	CGCGATCTTGATACCACCAATGGTGG	ACCACCGCTAAGGAGGCTTTGATGCGGAGAGCGACGGCGGAGCTACCACCATTGTTGGTATCAATATCAGCCAGTGTTAAGGAAGCTGTCGCGATCTTGATACCACCAATGGTGGCGGCGCCGCCGCCACTGAATTGCCTTCGAATTTGCATCATAAACGCCATAGCCGATTTGTTTAGATCAATTTTGAGTATGGCCGCTTACTTTCTACCTCTGTATCTTCTGTTTCTTCTTCTATATTTGGTGGTGGG
novel_mir28	GAGCTACCACCATTGTTGGTATCAAAGTCA	ACGGCGGAGCTACCACCATTGTTGGTATCAAAGTCAGCCAGCGTTGAGGAAGCTGTAGTGATCTTGATACCACCAATGGTGGCGGCGCCGCCGC
novel_mir29	CGGTTAACCGAAACCGACCCGATAATCA	ATTTTTTTTAAAATTTAATTTTAATTTCGGTTAACCGATAACCGAACCGACTAACCGAATCGGTTAATCGGTTAACCGATTAACCGATTGGTATCGGTTCCGGTTATCTATTTTGGGTTAATTCGGTTTCGGTTAACCGAAATTTCGGTTTCGGTTAACCGAAACCGACCCGATAATCACCCCTATTTAAGACCAACAATTTTCAAAGAGGAA
novel_mir30	TGAATCCGTTGAGAAAGTTAATGAATT	AATGAATTCATTATGAAAGTTGATGAATCCGTTGAGAAAGTTAATGAATTCAAAAATTCAGAATTTTTCACCCGGGTTCGAATCCCATGAGGGGCGAAAATTTATTTTTTTGGATTCAGTAACTTTCCCAGCGGATTCATCAACTTTCACTGTGAATTCATC
novel_mir31	TTATTGTGAGAACGTGAGAACCAA	CGTGAGAACCATTTTTATTGTGAGAACGTGAGAACCAAAAAATGATGAATTCACAGTGAAAGTTGATGAATCCGCTGGGAAAGTTACTGAATCCAAAAAAATAAAATTTTCGCCCCTCATGGGATTCGAACCCGGGTGAAAAATTCTGAATTTTTGAATTCATTAACTTTCTCAACGGATTCATCAACTTTCATAATGAATTCATTTGGTTCTTAGTTTTTAGGATAAGAATGGTTTTTATTTGAACCAAACTCTATATATATATATATATATATATAGGGGAGGGTTAACGTGAGAACCATTTTTATTGTGAGAACGTGAGAACCAAAAAATGATGAA

		TTCACAGTGAAAGTTGATGAATCCGCTGGGAAAGTTACTGAATCCAAAAAAATAAAATTTTCGCCCCTCATGGGATTCGAACCCGGGTGAAAAATTCTGAATTTTTGAATTCATTAACTTTCTCAACGGATTCATCAACTTTCATAACGAATTCATTTGGTTCTTAGTTTTTAGGATAAAAATGGTTCTTATT
novel_mir32	TAACTTACTCAACGGATTCATCAACT	CGTGAGAACCATTTTTATCATGAGAACGTGAGAACCAAAAAATGATGAATTCATTGTGAAAGTTGATGAATCCGCTGGGAAAATTACTGAATCCAAAAAAATAGAATTTTCGCCCCTCATGGGATTCGAACCCGGGTGAAAAATTCTGAATTTTTGAATTCATTAACTTACTCAACGGATTCATCAACTTTTATAACTAATTCATTTGGTTCTTAGTTTTTAGGATAAGAATGGTTCTTATT
novel_mir33	ACCGGAACCGAACCGAAACCGAT	TAAACGGTTTTCGGTTTTCGGTTAGAACCGGAACCGAACCGAAACCGATAAATTCGGTTAACCGAAAACCGACAAATTCGGTTATCGGTTCGGTTTCGGTTCCAGAGTTGAAAAAAAATTCGGTTATTGGTTAACCGATAACCGAACGGTTCGGAACCGAAAAACCGAAAAACCGTTTT
novel_mir34	CTTATAAGCTCTTAAAGCTTATTTTGA	AGACTTAGAGGGTGTTTGGCTAAGCTTATTTTAGAGAGCTTATAAGCTCCAACAACTTATAAGTTGTTTTAAGAACTTATAAGATATAATGTATTAAGAGCTTATAAGATGCTGAAGTGTTTGGATAACAAGAGTAAACATGGAAATAAGAAAATATATCAGGATTATTTTTGTAGTTTTGTTGTAACTTATAAGATCATTAAAAAAATAAGTTGGGTTTTGAAGCTTTTTTTTAATAGAGCTTATAAGCTCTTAAAGCTTATTTTGAAAACTTATAACCTCTCTAACAACTTAATTTGCCAAACACATTGATTGAGCTTATAAGCTCTTAAACAACTTATAAGCTGTTTTAAAGAGCTTATAAGCTCAGCCAAACACCCTCTAAGTCA
novel_mir35	TTCAGTAACTTTCCCAGCGGATTCATCAA	AAACTAAGAACCAAATGAATTCGTTATGAAAGTTGATGAATCCGTTGAGAAAGTTAATGAATTCAAAAATTTAGAAATTTTCACCCGGGTTCGAATCCCATGAGGGGCGAAAATTCTATTTTTTTGGATTCAGTAACTTTCCCAGCGGATTCATCAACTTTCACTGTGAATTCATCATTTTTTGGTTCTCATGTTC

novel_mir36	ATCCGCTGGGAAAGTTACTGAATCCA	CGTGAGAACCATTTTTATTGTGAGAACATGAGAACCAAAAAATGATGAATTCACAGTGAAAGTTGATGAATCCGCTGGGAAAGTTACTGAATCCAAAAAAATAGAATTTTCGCCCCTCATGGGATTCGAACCCGGGTGAAAATTTCTAAATTTTTGAATTCATTAACTTTCTCAACGGATTCATCAACTTTCATAACGAATTCATTTGGTTCTTAGTTTTTAGGATAAGAATGGTTCTCATT
novel_mir37	ATAGGAATGAATCTGGGCCTTTGATTTG	CCACTGAAGGGTAAATTTATTGTCCAGTTATTGGTAGTCCAAATTTATATATATATATATATATATATATATATATATATATATAGGAGATGGATCAGGTAGGAATGGATAATTTGGTAGGAAATAGGAATGAATCTGGGCCTTTGATTTGCTATATCAATGGTTGAGATTTAATTTTCGGGTGTGCTACAGTTTTGTGTTTTGTGTGCTACACGGATTATTGTGCAACACTATAACTCTTCTTGTGC
novel_mir38	TTCGTTTTCATACATTGTAGCTATAACA	AAAGTTTCATTCTTTTGAATTTCGTTTTCATACATTGTAGCTATAACAACAAATGCATAACAACAATTTGATGATCCTTGATTTGACACACGATGAGGCTCAACGATCATTAAATTATTGTTATGCATTTGTTGTTAAAGCTACTATGTATGAAGACGAAGTTCATTAGAGGGGAACTTC
novel_mir39	TATAAACGACATCGTAGAGAGATGA	ATTTGACATCTAAAAAATTTACTATAGACAAAACGGTTGTTTTAATTAGTTTTATAAACGACATCGTAGAGAGATGATATCGATTGTTTTGATCCCAACAAGAACCAGATGATAAAACAAACGAAAATATTTGATTTTTGTAATGAGAGTTGACGCAATGAATCATAGAAATAAAAAAATCTGATTTATTTTGATGGAATGACGATGTTTTATATCATCTCTCTATGATGTCGTTTATATCACTAACTGTTTTGCCTATTGTAAATTTTTGAGATATCAAGC

novel_mir40	GGCCGAGTTGGTGGTCTCCA	ACCCATTATTATGGAAGGAGACCGTCGGCTTGGATGGCCATCTTTGCAGGAGATTGTTGACGCTTTCTTTGCCCGGGAAACGTTCGGCAAAAATTCCGAGAAGACATAGGCGAAGTTCTTCCTCGGTAGATTGGTAGTCCGTCTTTTCCAAGTTGAGGCAGAAATTATCCAAAGCAACCTCAATTTTCTTGAGTTTCATGGCCGAGTTGGTGGTCTCCACCATATGAGTGGGG
novel_mir41	GAAGACACGAGATAGATCAAT	TAGGTATCCTCTTGATTCTCTGCTTCTTAACGAGTACTGGAGCGAGAGAGATGAGTGTTTCTCGACAAGATTTGGCGATGATCTGTGCAGGCTCACTCGGACAGATGTGTTGAAGAGCAGGGGATTGGATGAAGACACGAGATAGATCAATGTCGGGATCATGCTGGCGTTCTTCGTCTTCTATCAGATACTTT
novel_mir42	TCATGTGACAGAGGATGTAGTACACCTAA	ATAGGTGTATTACATCCTCTCTCATATGATAATATTTTATTAATATAAATTAAATTAATAAAATATTATCATGTGACAGAGGATGTAGTACACCTAA
novel_mir43	GTGCGAGCGTCGATCTGGTGGTCCGAG	GTCGGATCCCGGATGGATGCGTGCAGGTGCAACGAGGAAGGCCGTGTGCAGCACAGCAGCAGGACGGACGTGTGCAGTGCAACGAGGGGGCCAGGACGGTGCGAGCGTCGATCTGGTGGTCCGAG
novel_mir44	CACAGCAGCAGGACGGACGTGTGCAGTGCA	GTCGGATCCCGGATGGATGCGTGCTGGTGCAGCGAGGAAGGCCGTCTGCAGCACAGCAGCAGGACGGACGTGTGCAGTGCAGCGAGGAAGCGAGGACGGCGCTGGATGTGCAGCGAGGGGGCCAGGACCGTGCGAGCGTCGATCTGGTGGTCCGAG
novel_mir45	TTTAAACTCTAAACTATATATGTTTTATCA	AGGGTAAATATTAATTTAAACTCTAAACTATATATGTTTTATCAAAAATATCATGTACTTTCAAAAATATAAATTTAAACCCTAAATTATCATATTTGTATCAATTTAAATCCCAAACTATCCTGAACACAGGGGTATTTTTGATACAAATATGATAGTTTGGGGTTTAAATTGATATTTTTGAAAGTTCAGGATATTTTTGATAAAACATGTATAGTTTAGGGTTTAAATTGATATTTACCCA

novel_mir46	AAGTTGTGCACAACGAAAACTTTCTAAAAT	GTATAAAGTTGTGCACAACGAAAACTTTCTAAAATTGTTTTGTTATGCAACAATTTTAGAATGTTTTCGTTGTGCACAACTTTATAG
novel_mir47	AACCATGCATAAGACGTTGAAGATGATGCA	TTGAGAATTTGAGAACCATGCATAAGACGTTGAAGATGATGCACAAGATGATATAAAGTTGTGCATAACGAAAACATTCTAAAATTATTTTGTTGGGCAACAATTTTAGAATGTTTTCGTTATGCATAACTTTATATGTTTTATTCATCAATTTTATACATTTGTGCATAGTTCTCATGTTCTCAC
novel_mir48	CTTGCCGACCATACTGATATATCACC	AGATTGGTTGATTTGATGGATTTGTGGGCAATGACGAGTATTGTGATAGGTCTCGTTCGTCGTAATGGCATATTGCGTGCCTTGCCGACCATACTGATATATCACCACCCATAAACTGAGTCCTTTCCATTTCAAATTTCCAATTC
novel_mir49	GTTCGTGTCGGACAGATCCTTAACG	ATCATATTCGTGTTCGTGTCGTGTCCGTGTAGTACATTTCGTGTCGTGTTCGTGTTCGTGTCGGACAGATCCTTAACGGGTCGTGTTCGTGTTTGGCCTTATCAGGTCGTGTCGTTATCGTGTCGACACGATAACGACCCGACACGATAAGGCCAAACACGAACACGACCCGTTAAGGATCTGTCCGACACGAACACGAACACGACACGAAATGTACTACACGGACACGACACGAACACGAATATGAC
novel_mir50	GTCACAGGTTCGAACCCCACCAAA	AATGTGGATATGCAGTCCAACATGAGTCTCGTGCAACTTGTTTCGAGTCTTGACGTTCGATATGCATTAACTAGGGACAGCCCAATGGTTAAGGTGGGTTCCTGTGGCCGAGAGGTCACAGGTTCGAACCCCACCAAACGCCTCTATGGGTTTGAGTCTGTCACACCTGATTTTCCAAGTGTGGTTCACATG

novel_mir51	TCGTGGCTATAATTTGATATTCCT	TTGTGGAAATACATATATATAGCCATCCAAGTTAAGGAATGTCAAATTATAGCCACGAACATAAAAATGTCAAATTATAGCCAAATAGTACCAATAGTACCAATAGGACCAAATGGTCCTATCGGTACTATTTGGTGATAATAGTCCCAATAGGACCAACAACTTAGCTATAATTTGATATTTTTATGTTCGTGGCTATAATTTGATATTCCTTAACTTGAATGGCTATATATATGTATTTCCACAT
novel_mir52	AAAGGAAAGTGAGACATTTTAAATGGGACA	ATACTCCATCCGTCCCGCCTATAGTGTCCCCTTTCTTTTGGGTACGAGTATTTAGGTGAAAGGTATAAAGTAGTTAAAGTATTAAAAAGTGAGTAAGGTCCAAACTTTTTGAAAGGTTAATTATTTCCTAAAATGGAATGAGATATTTGTAATGAGACAATCCAAAAAGGAAAGTGAGACATTTTAAATGGGACAGAGGGAGTAG
novel_mir53	AAGGTGGGCCATGAGTGTTTTTTTGTAAAT	TTATACTCCCTCCGTCCACCAAAAACGTGCATCGATTTTGATGGCACGAGTTTTAAGAAAACTGTAATGAGTGTAAAGTAAATGTGTATAAAAAGGTGTGTCATTGGGATAATAAATGAATATATTAATGGATTGTGAGTGTAAAAAGGTGGGCCATGAGTGTTTTTTTGTAAATAAGTGGATAAGTTGAGGGTAAAAATGTAAAGTTGGTGTTTCTAAAAAAGGAAAGTGCACATTTTTGGTGGACGGACCGAAAAGGAATTAAATGCACGTTTTTGGTGGACGGAGGGAGTATAT

novel_mir54	TTGTTGACAAGTCGGTGTGGACACAGAA	ACCTGGAAATATCTGTAAATGAGATGTCACTTTTGTTGAATATTTCCACATCAAGTGGACCCACAGCCAACATATTTGACAATGGTGTGGGAAAAGGAGTTGGAAGAAGAGGTGCGGCCGCCAATGTTGACTCCAGCCGCCGTCACTTTTTGACATTGAGCCACTGTCCAGCCGCCGTTGTTGACAAGTCGGTGTGGACACAGAAGCCGCAATTTTTTACAATTGCGGATAACGGCTCAACCCCTCCTATAATTTTTCCTCACTTTTTCTTCCCTCAAAATGCACTCACCAAACTCTCTCTTGTTTATCTCACACTTTGCTGCACTTGCTCTTGCACACTTCTGATTGGGAAGAAATGAGCTCTATTTATAGGAGGGGTTGAGCCGTTATCCGCAATTGTAAAAAATTGCGGCTTCTGTGTCCACACCGACTTGTCAACAACGGCGGCTGGACAGTGGCTCAATGTCAAAAAGTGACGGCGGCTGGAGTCAACATTGGCGGCCGCACCTCTTCTTCCAACTCCTTTTCCCACACCATTGTCAAATATGTTGGCTGTGGGTCCACTTGATGTGGAAATATTCAACAAAAGTGACATCTCATTTACAGATATTTCCAGGG
novel_mir55	CCCTCTGTCCCATTTAAAATGTCCTACT	CTACTCCCTCTGTCCCATTTAAAATGTCCTACTTTCCTTTTTGGGTTGTCCCATTACAAATGTCTCATTCCATTTTAGGAAATAATTAACCCTTCAAAAAGTGTGGACCCTACCCACTTTTTAACACTTTAACTACTTTATACCTTTCTCCTAAATACCCGTGTCCAAAAGAAAATGGACACTTTAAGCGGGACGGAGGGAGTAC
novel_mir56	ATAAGTGGATAAGTTGAGGGT	CTCCGTCCACCAAAAACGTGCATCGATTTTGGTGGCACGGGTTTTAAGAAAACTGTAATGAGTGTAAAGTAAATGTGTATAAAAAGGTGTGTCATTGGGATAATAAATGAATATATTAATGGATTGTGAGTGTAAAAAGGTGGGCCATGAGTGTTTTTTTGTAAATAAGTGGATAAGTTGAGGGTAAAAATGTAAAGTTGGTGTTTCTAAAAAAGGAAAGTGCACATTTTTGGTGGACGGAC

novel_mir57	AGTGATGAAATTTCGATAGTTTGTGAGTT	ACACGATAGTTTTCATGGTGTGCACAACCGTGTAGTGATGAAATTTCGATAGTTTGTGAGTTTAGCAACTTAGAAATTGAATTCAGATTGCTATGTTGTTGATTTTATGTGAAAATTAGTGTGGTCGAAAATACACGGTTAGAACCGTGTACACTGTTTGAATCGTGTA
novel_mir58	GGTCATCTTGGGTAGGACAGTCCAAAAA	CGCGCATAGAAGATCATACAAACGATGAGTTTTTAGTACTCCGTCCAAAATTGTTCATTTCTTTTTGACATGATTATTTAGAAGAAGGGATAAAGTGGTTAAAATGATAAAAAAGTAGGTAGGATCCATAACTTTTGAAGAGTTAATTATTTCCTAAAAATGAAACAGGTCATCTTGGGTAGGACAGTCCAAAAAGGAATACGTGTCATCTTGAGTGGGACGGAGGGAGTATTTGATTTCAATGAGTATGTGTTACACCTCAAGTTTGTATGTCGATTGCGCA
novel_mir59	ATCTTGGGTGGGACGGAGGGAGTA	ATACTCCCTCCGTCCCACCCAAGACGGCTCCTTTCTTTTCGGCACTGTTTATTTAATAGAAGGGTATAAAGTGACTAAATTGATAAAAAGTAGGTAGGTCCCACAACTTTTAAAGAGATAATTATTTCCTAAAAAGGAAACGTGCCATCTTGGGTGGGACAGCTCAAAAAGGAAAGCGAGCCATCTTGGGTGGGACGGAGGGAGTAC
novel_mir60	AGGTCATCTTGGGTAGGAC	TAATACTCCCTCCGGCCCACCCAAGACGGCTCCTTTCTTTTCGGCACGGTTATTTAGGAGAATGGTATAAAGTGAATAAAGTGATAAAAAGTAGGTAGGTCCCACAACTTTTGAAGAGATAATTATTTCCTAAAAATGAAACAGGTCATCTTGGGTAGGACAGTCCAAAAAGGAATACGTGTCATCTTGAGTGGGACGGAGGGAGTATTT
novel_mir61	TCCGACTCGATAAGCCTGCTGGCG	ACTGCCAGGTCGGAGATGAAGGAGGAAAAACTCTCCGACTCGATAAGCCTGCTGGCGAAGGCCCTCAACATAGGTCCTTGCCCCGCCAGCAAGGCGCTTGTCTGCCCGGCCAGCTTCCTCTCCGCCTCGGCAGC

novel_mir62	GAACGGTTCCGGATCGGTCCGGG	ACCGGGTCGGACCGGAACCGGACAAAACGGGTCGGGTCTTGACCGGTCCGGGTCCAAGCGGTTCTGGAACCGGAACCGGAACCAGAACCGTTGTTTTAAAATTTAATGGGCTTTGAACGGTTCCGGATCGGTCCGGG
novel_mir63	GTTCGGTTTTCGGTTAACC	ATTTTCGGTTAACCGAAAACCGATCGGTTCCGACGGTTCGGTTTTCGGTTAACCGAAAAA
novel_mir64	TAATTTTAATTCGGTTTTTCGGT	CATCGGGGTTCGGGGTTCGGGGTTCGGGGTTCGGGTTTAGGGTTTAGGGTTTAGAATTAAAGTTAATCGACGGTTTGATTCTATTTATATATTTTATTTATTTTTAATAAGTTTCAATTTTTTAATTTTATTTTATTTATTTTATTTATTATTTTATTTATTTTTATTATTAATTAATTTTAATTCGGTTTTTCGGTTAACCGAAAACCGAACCGTCGGAACCGATC
novel_mir65	TTGGCACGTTTCCTAAAAAGGAAA	AATACTATCTCCGTCCCACCATTCTTGGCTCGTTTCCTTTTTGGGCTGTCCCAACAAGTTGGCACGTTTCCTAAAAAGGAAACAATTTTACACTAAAAAGTATCTCGAATTTCGTGTCACCTACTTTTTACACACAAAACACAACTCTCCTTAATCTACGTGCAAAAAGAAAGGAGCCATGATTCATGGGACGGAGGGAGTATA
novel_mir66	ATTTTTAGGGGAGCTTATAAGCTGTTGG	TAGAGAGATAAAATTGAAGAGAGGTGAAGTTGATAGTGTATGTGTAGAAAATAACGAACTATAATAAGATTATTTTGGTAAATTCATGATTGCTTATAAGTTTATGAAAAAATAAGTTAGGGTTGACCAACTTATTTTTAGGGGAGCTTATAAGCTGTTGGAGCTTATTTTCACAGCTTATAAGCTGTTTAGGAGCTTATTTTGTCAAACACTTTGCAATAGATTATAAGCTCCTAAACAGCTTATAAGCTCCGCCAAACACCCTCTAGAACTATCCACACACTTTACTTCTCTGTCTCTCTT

novel_mir67	AGGAAACGAGCCAAGAATGGTGGGACGG	CCTCCGTCCCATCATTCTTGGTTCATTTCTTTTTGCACGTAGATTAAGGAGAGTTGTGTTTTGTGTGTAAAAAGTAGGTGGCACAAAATTCGAGATACTTTTTAGTGTAATTTTTTTTTTTTTTTTGAAACGTGTCAACTTATTGGGACAGCCCAAAAAGGAAACGAGCCAAGAATGGTGGGACGGAGA
novel_mir68	CCGTCCCATGAATCATGGCTCCTT	GTTATACTCCCTCCGTCCCATGAATCATGGCTCCTTTCTTTTTGCACGTAGATTAAGGAGAGTTGTATTTTATGTGTAAAGTAGTGTGTGGTGCGAAATTCGAGGTACTTTTTAGTGTAAAATTATTTCATTTTTTGGAACGGGCCAACAATAGCGGAACAACCCAAAAAGGAAAATGAGCCAACAATAATGGGACAGAGGGAGTATAAG
novel_mir69	GACGTAGCTCAGTTGGTTAAGAGCT	AAGAGGCTACATGGTTTGCCCCCACGGACGTAGCTCAGTTGGTTAAGAGCTAGCAGATTTGTAAACAAGGATCAAGGATCGAATCCTAGCAGCCGCAGTGTGGGAGTTTCACCCTCTTTAGGGTGGCTCCTTGTGGATATTAGGTAAAAGTCCTCCTAGCTTCTTAACCGCTGAGTCACTGTGATTTACCCTCTC
novel_mir70	TCAATAAAGTTGTGGGAAGATATGATGA	ATGGCCCTCTTCTTATTCTTCCACAGCTTTCTTGAACTGCATATCACTGTATGCCTGCTGATCATTCTTTGTAGTTCAATAAAGTTGTGGGAAGATATGATGATGGTCAA
novel_mir71	TTCAATAAGGTTGTGGGAA	CTTCCACAGCTTTCTTGAACTGCAATCACTGTATGCCTCCTTACTGCAGCTGATCATACTTTGTAGTTCAATAAGGTTGTGGGAA
novel_mir72	AGATAGCGGCTGCGGGTTCCTCAT	CCCAACCCCCCTTTTCTATGTGGTTTGCAAGCTATTGCATAGGAACGTGGGGTTTACCCAGTGCACACCTAAGATAGCGGCTGCGGGTTCCTCATGATATCCAAAAAAAAAGAAGATTTCTTTCTACAACGAAGCTAGCAAAAAATTTATGTGGGATTATGGGGATGGA
novel_mir73	CGTGGCGAGGATCAATGGTGAATG	GTCACATGTCGCCTATTTGACACTTCACGCCTCGCACGACCCGTTGCCGAGATGGGGCAAGTCGTGGCGAGGATCAATGGTGAATGGCGAGTGTGGA

novel_mir74	ATAAAGTGAATAAAGTGATAAAAAGTAGGT	TTACTCCCTCCTCCGTCCCATTCAAAACGATCCATTTTTTTTGACACGGTTATTTAGGAGAATGGTATAAAGTGAATAAAGTGATAAAAAGTAGGTAAGTCCCACAACTTTTGAAGAGATAATTATTTCCTAAAAAGGAAACGTGCCATCTTGGGTGGGACAGCTCAAAAAGGAAAGCGAGCCATCTTGGGTGGGACGGAGGGAGTAT
novel_mir75	AGATTTACATGATTGTTATTTT	TCAGGGGCTAAGTGACAGCAAGAAAGTGAATCCGGGGAAGGTCGATCTACGCGTGGGAAATGGAGCAAGAGTTGTTGCTGAACGCGTGAGGACTTATAGATTAGATCTTCGAGATATAGACTAGATTTACATGATTGTTATTTTGTTCCTGC
novel_mir76	AGAGTTGCTGCCGAACGCGTGGGGACTT	TGTCTATAAATAATTCACATTCTTGGGTATTAGATACCGGCTGTGGCTCACACATTTGTAATAATGTGGAGGGGCTTAGTGACAGCAGGAAGGTGAATCCTGGGGAGGTCGACCTACGCGTGGGAAATGGAGCAAGAGTTGCTGCCGAACGCGTGGGGACTTATAGATTGGATCTTCCTTCGGGTTATAGACT
novel_mir77	TGCTTTAAAGGTTCCTCCAATTATGCTA	AGTGGTTGGATGGTTGGATTTTTAGGTGGTTGTTGATAAACATGGTCTTATGGCAGCTAGATTTCTCTTGGATTGCTTTAAAGGTTCCTCCAATTATGCTACCACC
novel_mir78	GATGAATCCGTTGAGAAAGTTAATGAAT	ATATATATATATATATATAGAGTTTGGTTCAAATAAGAACCATTCTTATCCTAAAAACTAAGAACCAAATGAATTCGTTATGAAAGTTGATGAATCCGTTGAGAAAGTTAATGAATTCAAAAATTCAGAATTTTTCACCAGGGTTCGAATCCCATGAGGGGCGAAAATTCTATTTTTTTGGATTCAGTAATTTTCCCAGCGGATTCATCAACTTTCACTGTGAATTCATCATTTTTTGGTTCTCACGTTCTCACAATAAAAATGGTTCTCACGTTAACCCTCCCCTATATATATATATATATAG

novel_mir79	TGAATTCGTTATGAAAGTTGATGA	CCATTTTTATCCTAAAAACTAAGAACCAAATGAATTCGTTATGAAAGTTGATGAATCCGTTGAGAAAGTTAATGAATTCAAAAATTCAGAATTTTTCACCCGGGTTCGAATCCCATGAGGGGCGAAAATTCTATTTTTTTGGATTCAGTAATTTTCCCAGTGGATTCATCAACTTTCACTGTGAATTCATCAATTTTTGGTTCTTAGGTTCTCACAATAAAAATGA
novel_mir80	CTCGACCGGTCACGGCGACTATGTGTA	TTGGTCCACCAACGTTAAAAAAGGGTAACGATCGGATCCGACTTAGTTGAGGACTAACTCGGATTGTGTACACGTGTGACTGTGTGTTAAGGTTAGCTACCTCGACCGGTCACGGCGACTATGTGTAGGTGGCCAC
novel_mir81	AAGAATTCATCTTGATCATCCATTTGCC	ATATATATATATATATATATATATATAGGGTTGGGTTATAATACAAACCCCCTTAAAATAAGAAATAAGAATTCATCTTGATCATCCATTTGCCATTAATCAATGGTTAAAGATTAAGTTATCACATTCCCTATAATTAAGGGACGGTTATTTTCGTAATATTCTGTAATTACCATTATACCCTAATTTGATTTAATCACAACCACACATCTTCAAAAATCAATGGTTCAAATTTGTTCTCATTTCTTATTTTTGAATGTGTTTTTATTTGAACCCAATCCTATATATATATATATATATATATGT
novel_mir82	AAGGAGCCGTCTTGGGTGGGACGGAGG	AATACTCCCTCCGTCCCACCCAAGATGGCTCGCTTTCCTTTTTGAGCTGTCCCACCTAAGATGGTCTGTTTCCTTTTTAGGAAATAATTATCTCTTCAAAAGTTGTGGGACCTACCTACTTTTTATCACTTTATTCACTTTATACCATTCTCCTAAATAACCGTGCCGAAAAGAAAGGAGCCGTCTTGGGTGGGACGGAGGGAGTATA
novel_mir83	AAATGAGCCGTCTTGGGTGGGACGGA	AAATACTCCATCCGTCCCATCCAAGATGGTTCGCTTTTCTTTTTGAACTGTCCCACCCAAAATGGCTCATTTCCTTTTTAGGAAATAATTATCTCTTCAAAAGTTGTGGGACCTACCTACTTTTTATCACTTTAACCACTTTATACCATTCTCCTAAATAACCGTGCCGAAAAGAAATGAGCCGTCTTGGGTGGGACGGAGGGAGTATTA

novel_mir84	TTGATCCCTTGGCCGAGAATGTGTTGTTGC	TGTGCTTTCATTGATGCTATTGATCCCTTGGCCGAGAATGTGTTGTTGCAGGAACATAGCCTCGAGGAAGTTTTTGCAGGATCGGCTGACGAGGAAGAGTGGGCACGTGAAGTTGCTAATATGACAAAGCCCACTCTAGAAATGAAGGAACT
novel_mir85	GTCCATACTTTTTAAATGGATAATTAT	CTCCCTCCGTTCCGCTTAAAGTGTTTAATTTCTTTTGGGCACGGGTATTTAGGAGAAATATATAAAATAGTTAAAGTGTTAAAAAGTGGGTAGGGTCCATACTTTTTAAATGGATAATTATTTCCTAAAATGGAATGAGATATTTGTAATGAGACAATCCAAAAAGGAAAGTGAGACATTTTAAATGGGACAGATGGAA
novel_mir86	TAAGCATAGGAACTCGATTTTTAGA	AATTGCAGTCCTGTGTGTGTTGGCCTAGTGGTAGAGTGGTTAATGCTCGAGGCCAAAGGTCTCGGGTTCGAATCCACCGTGACGCAATTTTTAAAAATTTCTGTATTAATTGAAAAAAAAGAAAAAAAAATGTATATGGTTAAGCATAGGAACTCGATTTTTAGAAAGCAAACTTTAGCAACCACCTCGTGTCAACCACCATTCCGACATCCACCGCAGTT
novel_mir87	GCCATGAATGGCGGGACGGAGG	TCTCCGTCCCACCATTCTTGGCTCGTTTCCTTTTTGGGCTGTCCCAACAAGTTGGCACGTTTCCTAAAAAGGAAACAATTTTACACTAAAAAGTATCTCGAATTTCGTGTCACCTACTTTTTACACACAAAACACAACTCTCCTTAATCTACGTGCAAAAAGAAAGGAGCCATGAATGGCGGGACGGAGG
novel_mir88	CGTCTTAACATTGATCCGGCCAC	ATGGTCTCCATTGGATATGACTGGGGTCTCGCCCGAGCTCATAACTCATCGTCTTAACATTGATCCGGCCACTAAACCAGTTCGGCAGAAGAAAAGAAGTTTTGCCGCGGAGCGAAGCCAGGTCATAAGAGATGAGGTAACCCAACTCCAGGAGGCTAAAATCCTGTACGAGGTTAAATACCCAACTTGGATGGCTAA

novel_mir89	AGGAAAGCGAGTCATCTTGAGTGGGACGGA	TTACTCCCTCCGTCCCACCTAAGACGGCTTCTTTCTTTTCGGCACGATTATTTAGGAAAATGATATAAAGTGATAAAAAGTAGGTAGCTCCCACAACTTTTGAAGATATAATTATTTCCTAAAATGGAAACGTGTCATCTTGGGTGGGACAACTCAAAAAGGAAAGCGAGTCATCTTGAGTGGGACGGAGGGAGTAT
novel_mir90	GCGCACTCCACTAGTCGAAA	TGCTTTTGAAGAAATGCAGCCACACGTGAGCTGCCGGCTTTAAAAGAGTGGCTCGCAAGGTGCGCACTCCACTAGTCGAAAAATCCCATTTAGCTCCGTCAATGGCTGCAACTTCGAGGGCC
novel_mir91	TCTTCCTCGAAGCTGCCACCTCGCTCG	ACATGCGTCCTAAGTCGGGGATGGCCTCATACGACCAAATATGTAAGGCCCAAACAGGATCATAGAAATGGTACGTCTTCCTCGAAGCTGCCACCTCGCTCGGGTGCCTCTTCAGCACATCAATCCCGTGATAGAGGACCTGGAATGAATAGGAGCCCCAAGGAAAACTCTGCCACATATCAAAATCCTCAGCCAACGCCCAAACCCATGGCTCGATGGCTTCCCTATCGCGGCAAAGGACGATGA

novel_mir92	GAAATGTGCGTTCAAAACAATCAAGCGG	GCAACTCTTTCAAATGACGTCAAACATTCTTGATACACGATGCCACCGTCAAATCTCGGTGGTGAAAAATTATTGTTCGCCGACAAAAACTCTTCGATATTATCTAACGACATCGGCCTACAATGTGATTCTTCCCATCCTGATTCTGTTGTTTCTTGATTGATTTCTTCCCAATCAGAACCTGAATTGATTTCTTCCGAATCAGAACCTGCTTCATGTCCTGGATTGATTTTTTCCAAATCAGAACCTGAATCAGAACTGGAAATGGGAAACGTCACTTGCTTGTTTTGAATTCACATTTTGCCACTCTTTCCTCCCCATTTTTTTTTCCAAATGAAAAGACTGACCCTTAAAATTGAATTTATGATATTTTGCTATCGCGCGCAAACACATTTTTTTCTTTTTCTTTTTTTATTTCGTTTATCTTTTATTTTTCTTACTTTTCCCAAAATTTGAGTTACAATATATTAATCAAATTTATTTTGGGAAAAGTAAGAAAAATAAAAGATAAACGAAATAAAAAAAGAAAAAAATGTGTTTGCGCGCGATAGCAAAATATCATAAATTCAATTTTAAGGGTCAGTCTTTTTATTTGGAAAAAAATTGGGGGAAAAGAGTGGCGAAATGTGCGTTCAAAACAATCAAGCGGCGTTTCCCATTTCCAGTTATGATTCAGGTTCTGATTTGGAAGAAATCAATCCCGGGCATGAAGCAGGTTCTGATTCGGAAGAAATCAATCCATGTTCTGATTGGGAAGAAATCAATCAAGAAACAACAGAATCAGGATGGGAAGAACCAAATTGTAGGCCGATGTCGTTAGATAATATCGAAGAGTTTTTGTCGGCGAACAATAATTTTTCACCACCGAGATTTGACGGTGGCATCGTGTATCAAGAATGTTTGACGTCATTTGAAAGAGTTG
novel_mir93	ACGTGCCATGAATATTGGGACGGAGGGA	CTATGTACTCCCTCCGTCCCAATATTCATGGCACGTTTTCCTTTTCGGGCTGTCCCATTAAGTTGGTCCGTTTCTAAAAAAGGAAACACTTTTTACACTAAAAAGTGTATCGAATTTCGTGTCATCCACTACTTTACACACAAAACACAATTCTCCTTAATCTACGTATAAAAAAGAAACGTGCCATGAATATTGGGACGGAGGGAGTATATGA

novel_mir94	AACTACCAGGCCCAAGCATC	CGTTTTCTGCGTCTTTTGGGGGGGGGGGTTGTGATAGGATACGGCCCAACTACCAGGCCCAAGCATCAGGCCCAATACAAGTAAGCCTGACATCAAGCTCGGCCTGGACAAGTTCGGCCCAAGAAGACTCCTAAAACCAGTGGGAACGCAGAAGGCT
novel_mir95	TGTTTTCGTTGTGCACAACTTTATAGAT	CGAACTATGCATAAGACGTGTAAAGATGATGCACAGAACGGTATAAAGTTGTGCATAACGAAAACTTTCTAAAATTGTTTTGTTGTGCAACAATTTTAGAATGTTTTCGTTGTGCACAACTTTATAGATTTTATTCATCAACTTTATACGTCTGTGCATAGTTCT
novel_mir96	CATTTAAAGTGTCCATTTTCTTTTGGG	AAGTTCTTATATTATACTCCCTCCGTCCCATTTAAAGTGTCCATTTTCTTTTGGGCACGGGTATTTAGGAGAAAGGTATAAAGTAGTTAAAGTGTTAAAAAGTGGGTAGGGCCCACACTTTTTGAAGGGATAATTACTTCCTAAAATGGAATGAGACATTTGTAATGGGACAACCCAAAAAGGAAAGTGAGACATTTTAAATGGGACAGAGGGAGTATTACACTTCAACTA
novel_mir97	ATGAATATGCTATGAAAGTTAATGA	ATATATATATATATATATATATATAGAGAAGGTTTAACGTGAGAACCATTTTTATTGTGAGAACATGAGAAATGAATATGCTATGAAAGTTAATGAATACGCTGCGAAAGTTAATGAATAAAAAAAAAATAGAATTTTCGCCTCTCATGGGATTCGAACCCTGGTGAAAATTCCGAATTTTTGGATTCATTAACTTTCGCAGAGTATTCATTAACTTTCATAGCATATTCATTTTGTTTTCGTAGTTCTTAGTTCTCACGTTAAAATTGGTTCTTATTTGAACCTCACTCTATATATATATATATATATATA
novel_mir98	AACATGACACGGATCCGACACGAAATT	ACGATCCGACACGAACACAAATGCAACACGAAAATTATCGGGTTACACGAACATGACACGGATCCGACACGAAATTATCGTGTCGACACGATAAGGACAAGAACACGATAAGGTTGAACACTAACCTGCTAATTTCGTGCGTGTTTGTGTCGTGTTCTCGTGTCGTGTCGA

novel_mir99	AATGTGTATAAAAAGATGTGTCAT	ATGTACTCCCTCCGTCCACCAAAATCGTTCATCGATTTTGGTGGCATGGGTTTTAAGAAAACTGTAATGAGTGTAAAGTAAATGTGTATAAAAAGATGTGTCATTGGGATAATAAATGAATATATTAATGGATTGTGAGTGTAAAAAAGTGGGTCATGAGTGTTTTTTTTGTAAATAAGTGGATAAGTTGAGGGTAAAAATGTAAGTTAGTGTTTCTAAAAAAGGAAAGTGCACATTTTTGATGGACGGATCGAAAAGGAATTAAATGCACGTTTTTGGTGGATGGAGGGAGTATAA
novel_mir100	GTTGGCTGAGGATTTTGATATGTGGC	TCGGGATCGGAGGACTACTATTTCAGAGCTCCGCAGCCGATTTATCAAACAACGGCTGGGTGATGATGCCGCAGACTACTTGAAGGCGGCTTTCATATTAGTAGCTTACCTCATCGTCCTTTGCCGCGATAGGGAAGCCATCGAGCCATGGGTTTGGGCGTTGGCTGAGGATTTTGATATGTGGCAGAGTTTTCCTTGGGGCTCCTATTCATTCCAGGTCCTCTATCACGGGATTGATGTGCTGAAGAGGCACCCGAGCGAGGTGGCAGCTTCGAGGAAGACGTACCATTTCTATGATCCTGT
novel_mir101	CGCTTAAGACCCATAGTTGATGGTCCG	AGTTCTGGTTCTTGTCCGGTTCTGGTCCGGTTCTGGTTCCAGACCCGCTCAGATCCGGACCGCTTAAGACCCATAGTTGATGGTCCGGGTCCGATCCGGATCCAGTGTCTAATGGGTCGATTCCAGAACCAGAACC
novel_mir102	TGAGAACTTGAGAACCATGCATAAGACGT	TGGTTCTTATATTCACACTTTAAAACTTCAACACCTCTCTCTCTTTGATCCACTCTCTATATAGAGGTTGGATCCAGTGAGAACTTATCTTTTTTGAGAACTTGAGAACCATGCATAAGACGTTGAAGATAATGCACAAAACAGTATAAAGTTGTGCATAACGAAAACTTTCTAAAATTGTTGCACAACAAAATAATTTTAGAAAGTTTTCGTTATGCACAACTTTATACCGTTTTGTGCATCATCTTCAACGTCTTATGCATGGTTCGCAGAGTTCGCAAAAAAGATGTGGTTCTCATTTGATCCACTCCCTATATATATATATATATAATTAAAAAAAAAGAACCC

novel_mir103	GAGCTTATTTTGCCAAACA	TAACAGCTTATAAGCTGTTTAGGAGCTTATTTTGCCAAACACTTTGCAACAGCTTATAAGCTCCTAAACAACTTATAAGCTGTTT
novel_mir104	AATTTGGACCCTAAAGCTGCCGGTGCAA	GTGCACCGCGCATTTTGGGCCATCAAAAAGCTGAATTTGGACCCTAAAGCTGCCGGTGCAA
novel_mir105	TGGTGTTTTGAAGGCTGTTCGGTA	TGGCATGCTTTCCCGAAAACCAACCGATATGGAGACATTCCCAATGGTGTTTTGAAGGCTGTTCGGTAGGCCCACAAGGCGTCATCCAAGCGTAGAGACCAGTCCTTTCGGTTGGGATTCACCACTTTCTCGAGTATTCCCTTGATTTCTCTATTGGAAAGCTCTGCTT
novel_mir106	AAGTTGATGAATCCGCTGGGAAAATTACT	GTGAGAACCAAAAAATGATGAATTCACAGTGAAAGTTGATGAATCCGCTGGGAAAATTACTGAATCCAAAAAAATAAAATTTTCGTCCCTCATGGGATTCGAACCCGGGTGAAAATTTCTAAATTTTTGAATTCATTAACTTTCTCAACGGATTCATCAACTTTCATAACGAATTCATTTGGTTCTTAG
novel_mir107	ATCGTGAGAACGTGAGAACCAAAAAATGAT	AGGTTAACGTGAGAACCATTTTTATCGTGAGAACGTGAGAACCAAAAAATGATGAATTCATTGTGAAAGTTGATGAATCCGTTGGAAAAGTTACTGAATCCAAAAAAAAGAATTTTTGTCCCTCATGGGATTCGAACCAGGGTGAAAATTTCTGAATTTTTGAATTCAGTCATCAACTTTCATAGCAATTCATTTGGTTTTTAGTTTTTAGGATAAAAATGGTTTTCATTTGAACCC
novel_mir108	GTTTTAATAAAAGTTGTTGAGTTA	TTATACTACCTCCGTCCCCCATATTCCTTCCTAGGAATATGGAGTGCGGGTTTTAATAAAAGTTGTTGAGTTATTGTGAGTGGAATAGGGGTCCCACATTAATATAATATTAAATTAGTTAGTGGTGGACCATAGGTGGTAAAAGGTGTAAATAAAGAATATTATTGAGGGTAAAGTTGGAAAGATAGTTTCCATAAATAGATAGGAAGGAAATTGGGGGACGTCCCAAAAAGAAAAACTAGGAAGGAATTTTGGGGACGGAGGGAGTATA

novel_mir109	AGCTTATAAGCTCTTATAACTTAT	AAGCTTATAAGCTCCAACAACTTATAAGTTGTTTTAAGAACTTATAAGATATGGTGTATTAAGAGCTTATAAGATGCTAAAGTGTTTGGATAATAAGGGTAAACATGGAAATAAGAAAATATATCAGGGTTATTTTTGTAGTTCTGTTGTAACTTATAAGATCATTAAAAAATAAATTGGGTTTTGGAGCTTTTTTAATAGAGCTTATAAGCTCTTATAACTTATTTTGAAAACTTATAAGCTCTCTAATAACTTAATTTGTCAAACACATTGATTGAACTTATAAGCTCTTAAACAGCTTATAAGCTG
novel_mir110	TGAGGCTCGAAAGTGGCTCAGCC	TCCGGCTCGTGAGGCTCGAAAGTGGCTCAGCCTCGACCCATTTTATGAAGTAATCCACAGCGACGACTAAGTATCGAAGCTGACCTGGTGCTCGATCGAACGGGCCAACGATGTCGATCCCCCACTGCATGAAAGGCCATGGACTCTGCAGGCTGCTTAGATCGGTCTGAGGCAGCTTTTTGACCGTGGAGTGAAGCTGGCACTTCGGGCACTTTTTGACGAGCTCGGC
novel_mir111	AGTGAAGTAAGTAATCGAGAAAGCAAGGA	GGGGATTGTTATGACTCGGAAAGATAGTGAAGTAAGTAATCGAGAAAGCAAGGACGCTATGAGTACTCGCTAATGCTTGCTCTTTACTAATATAAATAGGGCCTTTGATTGCTTACTTCGCTATCCCGGGATGATACTTTACTTATTCATGAACAAAGTCATAATTCTCA
novel_mir112	AATATCAGGCCTAGTACACGTA	ATGAGCTTAACTTCTTGAGTAGAGGGATAAAATATACCTGCATTCAGAGTGCCTTTAATATAGCACAGAATCCTCTTAGCAGCATTCAAATGGGATTGTGCCGGTATTTCCATGTATCTGCTGATTAGTTCAACTCCATAAAAAATATCAGGCCTAGTACACGTAAGATACCTTAGACTTCCAACTAGGCTTTTGAAATAAGTTGGATCCACCTGCGCTTCGCCGTCACTCATAAGTTCAC

novel_mir113	TGTCTTAATAATGGGCCGATTTT	GGTTGGGCTGAATTTATTTTAATGTCTGGGCTGGACTTCATTTTAATTAATTGCTGATGGGTTATGTCTTAATAATGGGCCGATTTTTATAATAAAGGAATGGGCCGAGAATTTAAAAGAAGTGGGCCGAAAATAAATGGGTTGGGCTCCAGTGAATTTAAATAATAGTTGGGCTTAGATTTAATTAATGGTTGGATTTTAGTTTAATT
novel_mir114	CGTGCCTCATCAACTGATTGCC	CCATCAGCTGATTCCCGTGCCTCATCAACTGATTGCCGTGCCACGGGATGAGTATGATGATCGGATGGAGAATGGGTCGGTGT
novel_mir115	AATGGCAGCAGAGTCGTGAACAAGAAAAGC	AAATGGCAGCAGAGTCGTGAACAAGAAAAGCTTCGTTCTTTGTTGTTGTAGCTGGGTAGCCTCCGATATGGCTAGGCTATTAAGTCAGAGATGGGCCTTGAGACGGGATCAACTACAATTGGCCTATCGCTTCTGTAGACTATTCCTGGTGAGTTGCCTACCCGAGCCCGAACTCGCTTAAGGGAAAAAGAGCAAGCAACTGAAGACATCACATCGCTTTCTTCCTCCAAGACTATGCTACCATTA
novel_mir116	ACTCGATGTAGATGTTCGCATAG	TCGTTGGATCACAGATACGATCCATGATGTGGTCCGAGAGAATATTCAAGGACTGAAGACGTGGTTTGAGTCTATGCTAGATGAGACTCGATGTAGATGTTCGCATAGTACTGAGGCATATACGTCATCCCGTCGTGGAACTACCCCACCGCCGATGCGGAGTGACGATCAGGGACGGCAACATTCTATTCGTACTCCTACGTCGGGTTCAGATGTACCCCAACAGACTCATCTATCAGATCCGACGT
novel_mir117	TAAATTCCTATGACCGAGTCGTTCTG	AGGTGGTCTAAATTCCTATGACCGAGTCGTTCTGGCCCCTGTGAACATCTTTTTTTAATGTATTAAAGAGGGCCTCTCTAACCGTGAAAAGTGGTGGGTGCCCACTAACGGCCATTCCTGGCTCGGCTCCGATAACGCAATTCCGGGAGGAGTCGGTAGTTGGGCACTGGATCCCTTCGGACCTGGAGAACGTGTGACGCTGGGTCGGAGTTTGGTGAACCA

novel_mir118	TAATTCTCCTGGCGTAGATCTACTTGTTGT	ATTTCTCCTGACACATTACTAATTCTCCTGGCGTAGATCTACTTGTTGTTAATAGATCGGTAAGAGTATTGTTCCGATAGATAACTCTTCTATAGAGTTCATTAATATCTGAGCTCATTAATTTACCCCCATCTATCTGAATGATGGGTCTTAACTCAGGAGGAAGAACCGGTAATAGACACAAAACCATCCATTCTGGTTCTATATTTGTTCGAAGAAAA
novel_mir119	AAACAAACCAAACCGTTCGTGAGCTACT	TTGGTAGGGCTGTAAACAAACCAAACCGTTCGTGAGCTACTCGGAGCTCGGCTCGAAAAAAGCTCGTTCGAAATTCGATCGTTTTGTAAACGAGCCGAACTCGAGCCTCGAATCGAGCTCGATAAGTTTATCGAGCCGAACTCGAGCTTAGAGATGTTCGGCTCGTAGGCTCGCGAACATGTTTGGTTCGAACTCAAACGAGCCAG
novel_mir120	GGAAAAAATCGAAGCCGCTT	CAGATTGCGGTGGAGGAAAAAATCGAAGCCGCTTTATTGGACGATGGCTTTTCTCCAGATGCGATTTT
novel_mir121	CAGGTCTGATTTTCGTCGGTCTGAAC	TTCCCAGAGATTGAACCGTTAGATCGCCTTGAGTTTTGGACAGCAGCTTCACAACATCTGGGCCAACGTTTTAGACGGTGGAGATCGGATTTTGAGTTTTACAGGTCTGATTTTCGTCGGTCTGAACAGTGAAGAATTTTTGGTGAT
novel_mir122	AAGTTTTCGTTATGCACAATTTTATACT	TATATATATATATATATATATAGGGGTTGGATCCAGTGAGAACTCATCTTTTTTGAGAACTTGAGAACCATGCATAAGACGTTGAAGATGATGCACAAAACGGTATAAAGTTGTGCATAACGAAAACTTTCTAAAATTATTTTGTTGTGCAACAATTTTAGAAAGTTTTCGTTATGCACAATTTTATACTATTTTGTGCATTATCTTCAACGTCATATGCATGGTTCTCAAGTTCTCAAAAAAGATGAGTTCTCACTTGATCCAACCCCTATATATATATATATATATT

novel_mir123	TCGATCCGGATAAAATCCATCCATTG	TCACTAGGTATTGGTTCTGGTTCCAAAACCGGAACCGGACCGGTTGGACCATCAAAATATGGATCTAAAGCGAGTCTGGAATCGGAACCGGAACTAGAACCGCTGCTTTAAAATTTAATGGGTTTTGAACGATTCCGGATCGATCCGGATAAAATCCATCCATTGACATCTCTAATTAAGGCTATCAGAAAGAAAAAGGAAAAAAAAAAAAAGATAATATAAGGAAAAAGCAATGATAGTTGCAAGAAGATGTCTAGTGC
novel_mir124	TTCGCGGGTACGGAAATGGTTTGA	CCGCGGGTATCCGTCCGATTATTCGCGGGTTTGGTGTGCATTTCGTATCCGCGGGTACGTTCGTGGTTGAAAACTAGGACCCGTTTACTTCGCGGGTACGGAAATGGTTTGACAATACCCGTACCCGTGAAACCCGTATACCTGCGAATTTTACCCGCAAAAACACCTGCGAATTACCCGCGA
novel_mir125	CCTCTTTCCTTCTTTCTTATGCCA	GGACTCCACCCCTTTCTTCACTCCTCTTTCCTTCTTTCTTATGCCAAATTTTTTTCTTAAACATTTTTGGCGTGAGAAAGAAGGAAAGAGGAGTGGAGAAAGGGGTGGAGTTG
novel_mir126	TGGATAAAACTAGCTCATTGACA	ATAAATAAGTATGAGTTCTGGTTCTGGAATCGACCCATTAGATATTGGATCCGGACCTGACCCGGACCATCGACTATGGGTCTTAAACGGTTCGGATCTGAACGAGTCTGGAACCAGAACCAAAACTAGAATCGAAATCGCTGCTTTATAATTTAATGGGCTATGAGTGATTTCAGATCGATATGGATAAAACTAGCTCATTGACAAGCTTATTTAA
novel_mir127	CGCGAACTAGAACCAATAAAT	AGGTTGGTTTTGAGTGACTAATGTGGTGACATGCCTTCCACTTAATAGGTGGGATTAGGGATGTCAATAGGTGGATTTTACGCGAACTAGAACCAATAAATAAGTAATGGTTCTAGTTCTGGAACCGACCCATTAGATACTAGATCCGGACCAGACCC
novel_mir128	ATCCGGCAGTAGATCCTTTAGATT	ATTTGCACATTTAGATGCTACTACCGTACTATCAAGAGGATTGGCTGCCAAAGGGATCTATCCGGCAGTAGATCCTTTAGATTCAACCTCAACCATGCTTCAACCTCGGATCGTTGGTGAGGAACATTATGAAACTGCGCAAAG

novel_mir1 29	ATTTTTTGGATTCAT TAACTTTCGC	ATGATGGATTGGCTGTGAAAGTTGATGAATCCGCTGAGAAAATTATCGAATCCAAAAAACAATTTTA TTTCCTTTTTAATTTTCACTCCTTATGAGATTAGAACTTAGTGAATTTTTTGAATTTTTTGGATTCATTAA CTTTCGCAATTGACACATTAA
novel_mir1 30	TCTCCGCATCTTGG GTGGTGGC	TCTCGATAAGGTTTTAGATGAGTATGAGTTATGACCATCACTTTGACTTGTTATGAGTGAGGGTTATAT GTTCTAACCCAATATTCTCCGCATCTTGGGTGGTGGCAGTGCTTGTATGTCATGCTAGTTACCTGTGTC GAGT
novel_mir1 31	CGAGGTAATTTTTA GTGTAAAAAG	GCCTCGACACGTCGGTGATGGGAGCTGACTACAGAACCTTTGTTGAGATTGTGTTTGAGAACAGAG AAAACATCATTCAGAGCTGGCATCTTGATGGATACTCGTTCTTTGTAGTAGGGTGAGCCCTCATAAAA GTCGCTAAAGTAATACTCCCTCCGTCCCAATATTCATGACATGTTTCTTTTTTGCATGTAGATTGTAGAT TAAAGAGAGTTGTGTTTTGTGTGTAAAGTAGTGGATGACACGAAATTCGAGGTAATTTTTAGTGTAAA AAGTGTTTCCTTTTTTAGAAACGGACCAACTTAATGGGACAGCCCGAAAAGGAAAACGTGCCATGA ATATTGGGACGGAGGGAGTATTACTTTAGCGACTTTTATGAGGGCTCACCCTACTACAAAGAACGAG TATCCATCAAGATGCCAGCTCTGAATGATGTTTTCTCTGTTCTCAAACACAATCTCAACAAAGGTTCT GTAGTCAGCTCCCATCACCGACGTGTCGAGGCG
novel_mir1 32	ATATAAACGACATC GTAGAGAGAAGAT A	GATATAAACGACATCGTAGAGAGAAGATATCAATTGTTTTGATCCCAACTAGAACCAGACGATGAAA CAAATGAGAAAACTTGATTTTTATAATGAGAGTTAACGCGATGAATCCTAAAAATCAAAAAAACTTA TTTATTTTGATGGAACGACGATATTTTATGTCCTCTCTGTACGATGTCATTTATATCA
novel_mir1 33	GTTGGTCGAGGAGT CTTCTTGG	AGATATGCATCCGAATCTTCTACGTCTCCAATTGCGATTAGAGTTGTAGTTGGTCGAGGAGTCTTCTT GGGCCGAACTCACATGGGCCGAGCTTAATGTCGGGGCCCATTTATATTGGGCCTGATGCTTGGGCCT AGTAGTTGGGCCGTATCCTATCATATCC

novel_mir134	AGTTTCTTGATAGAGCTTAA	AGACAGAGATTGACAGTTTCTTGATAGAGCTTAAACATGAAAGAAAGATGTGGAAATTCATATATATAGCCATCCAAGTTAAGGAATGTCAAATTATAGCCACCAACATAAAAATGTCAAATTATAGCTAAGTTGTTGGTCCTATTGGGACTATTATCACCAAATAGTACCAATAGGTCGTCC
novel_mir135	TCGACGGATTCATCAACTTTCACAATGAAT	AAGAACCATTTTTAACGTAAGAACTAAGAACCATAAAATTTCGCTATGAACAAAATGAATTCGCTATGAAAGTTGATGCATCCGTTGGAAAAGTTAATAAATCCAAAAATTAAATATTTTTCACCCGGGTTCAAATACCATGAGGGAAGAAAATTCCAAAAAAATTTGGATATTTTATGGATTCGGTAAATTTTTCGACGGATTCATCAACTTTCACAATGAATTTATAATTTTTTGGTTCTCCCGTTCTTACGAAAAAAATGGTTCTC
novel_mir136	TTGTGTTTTATGTGTAAAGCAGTAGGTG	TTCATGACCCCTTTCTTTTTTGCACGTAAATTAATGAGAGTTGTGTTTTATGTGTAAAGCAGTAGGTGATACGAAATTCGAGACACTTTTTAGTGTAAAAAGTGTTTCCTTTTTTGGAAACGGACCAACTTAATGGGAGAGCCCAAAAAGGAAAACGTGTCAACAATAATGGGACGGATGGAG
novel_mir137	ACTCCTGGCGTGTTCGCAGCAC	CGAGCTACGAGGCCCCTCTCCGAATCGTCGTCCACAATGGCACGACGGACAGTCTCAGTCACAGCAGCAATGGAGGCCTCGTCTAGTCGAACACTCCTGGCGTGTTCGCAGCACACGTGGTCTGGCTCTGGCTCCTCCTCATGTCTACGACGCTTCGACTGCGATCGAGATGCGCCAGGTCGACTCGGCTCA
novel_mir138	AAGCCTGTGGGTTGGTCCAA	TTGGCCCAAAAAAGCCTGTGGGTTGGTCCAATTGAACAATCACTTTTTTTGGGCCAT
novel_mir139	AACAATAATGGGACAGAGGGAG	GTCCTTCCGTCCCGATATTGTTGGCATTAGATTAAGGAGAATTGTCTTTTGTGTGTAAAGTAGTGTGTGACACGAAATTCGAGGCACTTTTTAGTGTAAAATTATTTCATTTTTTGGAACGGGCCAACAATAGCGGAACAACCCAAAAAGGAAAATGAGCCAACAATAATGGGACAGAGGGAG

novel_mir140	CTCGACCGGTCACGGCGACTATGTGT	TGAGTTGGTCCACCAACGTTAAAAAAGGGTAACGATCGGATCCGACTTAGTTGAGGACTAACTCGGATTGTGTACACGTGTGACTGTGTGTTAAGGTTAGCTACCTCGACCGGTCACGGCGACTATGTGTAGGTGGCCACCTCC
novel_mir141	GACTGTGTGTTAAGGTTAG	ACGTGGTATGAGAATGTGTTGGTCCACCAACGTTAAAAAAAGGGTAACGATCGGATCCGACTTAGTTAAAGGCTAACTCGGATTGTGCACACGTGTGACTGTGTGTTAAGGTTAGCTACCTCGACCGGTCACGGCGACTATGTGTAGGTGGCCACCTCCATAGTGCGC
novel_mir142	AACGTGCTATGAATATTGGGACGGA	CATATACTCCATCTGTCCCATTAAGTTGGCCCGTTTCCAAAAAAGTAAACACTTTTTACACTAAAAAGTGTTTCGAATTTCGTGTCACCCACTATTTTACACACAAAACACAATTCTCCTTAATCTACGTATAAAAAAGAAACGTGCTATGAATATTGGGACGGAGGGAGTATATA
novel_mir143	CCTGTAAGCTCAATTACCCAAAT	GAGGGTGTTTGGCTGAGCTTATAAGCTCTTTAAAACGGCTTATAAGCTGTTTAGGAGCTTATAATCTATTGCAAAGTGTTTGTCAAAATAAGCTCTTAAACAGATTATAAGCTGTGAAAATAAACTCCAACAGCTTATAAGATCTCCTAAAAATAAGTTGGTTAACCCTAACTTATTTTTTCATAAACTTATAAGCAATCATGAATTTAGCAAAATAACCTTATTATGATTCGTTATTTTCAAATCCTATTAACTTCGCATCTCTTTAATTTTCTCTTTCTAATTTACGATTATTTCCGGCCTGTAAGCTCAATTACCCAAATACTTTG
novel_mir144	ATAAAGTAGTTAAAGTGTTAAAAAGT	CGTCCCATTTAAAGTGTCTCATTTCTTTTGGGCACGGGTATTTAGGAGAAATGTATAAAGTAGTTAAAGTGTTAAAAAGTGGGTAGGGCCCACACTTTTTGAAGGGATAATTACTTCCTAAAATGGAATGAGACATTTGTAATGGGACA

novel_mir145	GAGTTGTAGTTGGTCGAGGAGTCTTCTTGG	AGATATGCATCCGAATCTTCTACGTCTCCAATTGCGATTAGAGTTGTAGTTGGTCGAGGAGTCTTCTTGGGCCGAACTCACATAGGCCGAGCTTAATGTCGGGGCCCATTTATATTGGGCCTGATGCTTGGGCCTAGTAGTTGGGCCGTATCCTATCAGAAACCTTATAGCATCTGAATTGCACGCCCATCTTAATCCCTATAATGACATATCA
novel_mir146	CAATGTATGTTGAATTAAACATCCAAGA	ATGCTAGGTATATAATCAATGTATGTTGAATTAAACATCCAAGATAGATCCTCGGGAGAGGGTTAAAATCAGATTTTCCTACAATTGGTATCAGAGCCTGGGATCAGTATCTTGGCTCTGTTGGATTAATTCACGTTTGATTATTACGTGCGCAA
novel_mir147	CCATCTTGGATGGGACGGAGG	AATATTCACTCCGTCCCATTCAAGATGACTCGCTTTCCTTTTTGAGCTATCCCACCTAAGATGACACGTTTCCTTTTTAGGAAATAATTATCTCTTTAAAAGTTGTGGGACCTACCTACTTTTTATCAATTTATTCACTTTATATCATTCTCCTAAATAACCGTACCGAAAAGAAAGGAACCATCTTGGATGGGACGGAGGGAGTATT
novel_mir148	GGTTCGGGGAAAGGATCCCCT	ATATTTGTTTCGGTAAATATGCTCTTCAGGCACTTGAACCCGCTTGGATCACGTCGAGACAAATCGAAGCGGGTCGCCGGGCAATGACACGAAATGCACGCCGTGGTGGAAAAATATGGGTCCGCCTCTTTCCAGACAAACCAGTTACAGTAAGACCTGCTGAAACGCGTATGGGTTCGGGGAAAGGATCCCCTGAATATTGGGTAGCTGTTGTTAAACCGGGACGAATAC
novel_mir149	ATCGTGCTCAAGCTTGGTTTGTTTAGTATC	TAGCTAGGGGTGTAATCGAACCGAGCCGAGCCGAACACCATCGTGCTCAAGCTTGGTTTGTTTAGTATCGAGCCGAGTCTCGAGTTTTATTTATCGAATATTTCGAGCTCACGAGCTTATTCGAGCTTTAGTCGAACCCAAACAAGCTT
novel_mir150	GCTAAGGTGAAAGCCATTGAG	GTAGTCGAGGTGTGGCAGTTGATAGGGCTAAGGTGAAAGCCATTGAGGAATGGCCCACGCCCAAGACATGCTCCGAGGTAAGATCTTTTCATGGTTTAGCAGGATTTTATCGTCGTTTTGTGCGTGATTTTTCATCTATTGCTGCACCATTGACTGA

novel_mir151	TCATCAACTTTCATAGCAATTC	ATTTGATCCATCTCCTATATATATATATATATATATATATATATATGGGAAGGTTAACGTGAGAACCATTTTTATCATGAGAACGTGAGAACCAAAAAATGATGAATTCATTGTGAAAGTTGATGAATCCGTTGGAAAAGTTACTGAATCCAAAAAAAAAGAATTTTTGTCCCTCATGGGATTCGAACCAGGGTGAAAATTTCTGAATTTTTGAATTCAGTCATCAACTTTCATAGCAATTCATTTGGTTTTTAGTTTTTAGGATAAAAATGGTTTTCATTTGAACCCAACTCTCTCTCTCTCTCTCTCTCTCTATATATATATATATATATATATATAGGTAGTGGATCAAA
novel_mir152	TTTAAACGGGACGGAGGGAG	CGTACTCCCTCTGTCCCATTTAAAATATCTCACTTTCCTTTTTGGATTGTCCCATTACAAATGTCTCATTCCATTTTAGGAAATAATTATCCCTTCAAAAAGTGTGGACCCTACCCACTTTTTAACACTTTAACTACTTTATACATTTCTCCTAAATAACCGTGTCCAAAAGAAAAGGGACGTTTTAAACGGGACGGAGGGAGTACT
novel_mir153	ACGTCTTATGCATGGTTCGCAGAGTTC	CATATATATATATATATATAGGGGTTGGATCCAGTGAGAACTCATCTTTTATGAGAACTTGAGAACTATGCACAAACGTATAAAGTTGATGAATAAAACCTATAAAGTTGTGCATAACGAAAACATTCTAAAATTGTTGCACAACAAAATAATTTTAGAAAGTTTTCGTTATGCACAACTTTATACTGTTTTGTGCATTATCTTCAACGTCTTATGCATGGTTCGCAGAGTTCGCAAAAAAGATGTGGTTCTCATTTGATTCACTCCCTATATATATATATATATA
novel_mir154	CAAGATGACTCGTATTCCAT	ATACTTCCTCCCTCCCATCCAAGATGACTCGTATTCCATTTTGGACTGTCCCACCCAAGATGACATGTTTCCTTTTAAGGAAATAATTAACTCTTCAAAAGTTGTGGATCCTATCTATTTTTTTATCACTTTAACCATTTTATCTCTTCTCCTAAATAACCATGCCGAAAAGAAATGAGCCGTCTTGGACGGGACGGAGGGAGTAC

novel_mir155	CGTAGGAGCTTATAAGTTCCGAA	AAGAGGGTGTTCGGCTAAACTTATTTTAGAGAGCTCATATGATGTGTCAAGAGCTTATAAGATGTAATGTCTCAAAAGCTTATAAGTTGTCAAAATGTTTGGATAATTGAACTTATAAGCTAGAGAAAAAATCGTGAGTTAGAGAGAAAATTGACATGTGAAGTTGATAGTGTATATGAAGGAATAATGAACCATAGTAAAATTATTTTGGTAAATTCATTATTATTTATAAGCTTATGAAAAAATATGTTAGGATTGAGCAACTTATTTTTAAGAGAGTTTATAAACTGTTGGAGCTTATTTTCACAATTTATAAGTTGTTTATGAATTTATTTTGCTAAACACTTCGTAGGAGCTTATAAGTTCCGAAACAACTTATAAGCTATTTTGACAACCAAACACCCTCTA
novel_mir156	GCTTTAGACCCGGACCGTTTAGATCCATAG	CGGTTCTGGTTCTGATTCCGGTTCCAGACTCGCTTTAGACCCGGACCGTTTAGATCCATAGTTTGATGGTCTGGTCCAATTCGGATCTGGTTCCAGAACCAGAACTA
novel_mir157	AGACTTGTAAATAGACGGGTTTCTT	ATGGGTCTTAAACGGTCCGGATCTGAGCGGATCTAGAACCAGAACCGGACAAGAACCAGAACTGAAATCGAAACCGTTGCTTTATAATTTAATGGGTTATGAGTGATTTCGAATTGATATGGATAAAACTAGCTCATTGACAAACATATTTAAACTTAGACTTGTAAATAGACGGGTTTCTTTCGGACCAGAACCAATAAATAAGTAATGGTTCTGGTTCTGGATCCGACCCATTAGACACTGGATCCGGACCGGACCAGGACCATCAACTATGGGTCTTAAGCGGTCCGGATCTGAGCGGGTCTGGAACCAGAACCGGACCAGAACCAG

novel_mir158	CTATTTGATTGTCTTCTTCAA	TGTTACTTCCTCCGTACAGCTTAAAGTGATCATTTTCTTTTGGACACGAGTATTTAGAAAAAAGATATAAAGTAGTTAAAGTGTTAAAAAGTGAGTAGAGTCCATACTTTTTAAAGAGATAATTATTTCCTAAAATGGAATGAGACATTTGTAATAGGACAACCCAAAAAGAAAAGTAGGACATTTTAAATGGGACAGATGGAGTATTTCTTTTCGGCAAACACATGCACGATCATAATAAACATTCCTATTTGATTGTCTTCTTCAAAAACTTTTGTGTTTACTTTAGTCTTTTGGAGAATTTGATAACTTTTGGGAGTGATC
novel_mir159	CTGGCGGAGTGCAAAGCAGCG	GGAGCCGTATCCTGGCGGAGTGCAAAGCAGCGATGCATTGCGGACTCTACCATGGAAGCCGAATATGTAGCCGCTTCGGAGGCTTCTAAGGAGGCTGTATGGTTCA
novel_mir160	TGCAACAGCTTATAAGCTCCTAAACA	GGGTGTTTGGCTGAGCTTATAAGCTCTTTGAAACAACTTATAAATTGTTTAGGAGCTTATTTTGCCAAACACTTTGCAACAGCTTATAAGCTCCTAAACAATTTATAAGTTGTTTTAAAGAGCTTATAAGCTCAGCCAAACACTA
novel_mir161	TTTTGGTGGCACGGGTTTTAAGAA	ATATACTCCCTCCGTCCACCAAAAACGTGCATCGATTTTGGTGGCACGGGTTTTAAGAAAACTGTAATGAGTGTAAAGTAAATGTGTATAAAAAGATGTGTCATTGGGATAATAAATGAATATATTAATGGATTGTGAGTGTAAAAAGGTGGGCCATGAGTGTTTTTTTTGTAAATAAGTGGATAAGTTGAGGGTAAAAATGTAAGTTAGTGTTTCTAAAAAAGGAAAGTGCACATTTTTGATGGACGGATCGAAAAGGAATTAAATGCACGTTTTTGGTGGATGGAGGGAGTATAA
novel_mir162	GTCAATGGGTGGGTTTTATCCGGA	ACAGGCACTGCACTTAGGGATGTCAATGGGTGGGTTTTATCCGGACCAGAACCAATAAATAAGTAATGGTTCTGGTTCTGGATCCGACCCATTAGACACTGGATCCGGACCGGACCAGGACCATCAACTATGGGTCTTAAGCGGTCCGGATCTGAGCGGGTCTGG

novel_mir163	GGGAGAAGGAGAAGAGAAGAAGAAGC	AAATATAAAGTCTTAAAGGCATTGGTAAAGTCTTAGAGGCCATTGGAGGGAGAAGGAGAAGAGAAGAAGAAGCGCTGCTGTGTGTTTTCTTTCTTTTAGACCGTTGCAACCTTAGTTTATATTA
novel_mir164	GGGTTAGAGAGAGAAAATCATAGTTAGAT	ATTCTCAAAGTTCTTCAATATAGCTCGATTGATTATGAGGGTGTTTCTTTAAATTTATTTTAAAGTACTTATAAGCTTCAAGAGCTTATAAGATATTTCAAGAGCTTATATAATATAATATTTAAATAGTTTATAAATTGTCAACGTGTTTGTATAATTGAGCTTATAAGCTAGAGAAAGAATCATGGGTTAGAGAGAGAAAATCATAGTTAGATATAGAATTGATGAGAGG
novel_mir165	ATGAAAGATATGCTACTATTCGTA	GAAAGATGTGCTAAACTAATATATTTAGATATGTGCTACGAATGGATGAAATATGTGCTAAACTTTCATGTGCAATACATTAATTCTGATTGTGCTACACAATATGAAAGATATGCTACTATTCGTACTTTCGTACATTAAGGAGCTTTCTCACCATAACCAACCCCTATATATATATATATATATATATAGGGGTTGGTTATGGTGAGAAAGCTCCTTAATGTACGAAAGTATGAATAGTAGCATATCTTTCATATTGTGTAGCACAATCAGAATTAATGTATTGCACATGAAAGTTTAGCACATATTTCATCCATTCGTAGCACATATCTAAATATATTAGTTTAGCACATCTTT
novel_mir166	ATCAGAACCTGCTTCATGTCCTTGATTGAT	TTGGTTCTTCCCATCCTGATTCTGTTGTTTCTTGATTGATTTCTTCCCAATCAGAACATGGATTGATTTCTTCCGAATCAGAACCTGCTTCATGTCCTTGATTGATTTCTTCCAAATCAGAACCTGAATCAGAACTGGAAATGGGAAACGCCGC
novel_mir167	ATATGGATAAAACTAGCTCATTGA	GTCAATGGGTGGGTTTTACCCGGACCAGAACCAATAAATAAGTAATGGTTCTGGTTCTGGATCCGACCCATTAGACACTGGATCCGGACCGGACCAGGACCATCAACTATGGGTCTTAAGCGGTCCGGATCTGAGCGGGTCTGGAACCAGAATTGGAACCGAAACGTTGCTTTATAATTTAATTGGCTATGAGTGATTTCAGATCGATATGGATAAAACTAGCTCATTGAG

novel_mir168	AATGAGCCGTCTTGGATGAAACGGA	ATACTCCATCCGTCCCATCCAAGATGGTTCGCTTTTCTTTTTGGGTTGTCCCACCCAAGATGGTCTGTTTCCTTTTTAGGAAATAATTAACTCTTCAAAAGTTGTGGGCCCTACCTATTTTTTTATCACTTTAACCACTTTATTTCTTCTTCTAAATAACCGTGCCGAAAAGAAATGAGCCGTCTTGGATGAAACGGAAGGAGTAG
novel_mir169	TTACTTTGCATGATTAGGTTGGATAGATAA	ACCTTAAGATGGTGTTTACTTTGCATGATTAGGTTGGATAGATAAAATTAATCCAACCTAATCAAGTGTTTACTTTTAAGGA
novel_mir170	GGGATTATGGATGTCGCTCA	AGGGCGGATGTGCCATGGGCACGCAGTGAAGTTGGGAGTGGACAGTGTTTTGCCGGTGCAGAATTCCTTGGTTCATTTTTATGCTTGTTGTGGGATTATGGATGTCGCTCA
novel_mir171	AAAAAGATTTGTCTAAGCCACTT	GTCAGATAGATAGATTTGCCTCAATGTATGCACTTCCGTGTACTAGCTAAAGCGTTATGAGGTTAGCGACAAAAAAAGATTTGTCTAAGCCACTTCGTTTCTTTTTGTCCAAGTCACTTCTTTTTTTGTCCAAGTTGCTAAGTAATTGCCTCATAGATCCTATATCTATCTATATGAA
novel_mir172	CAATTTCATAACTAGTTGGTGCGT	TAACGCTTTAGCTAGTACACGGAAGTGCATACATTGAGGCAAATCTATCTATCTGACGAGCTAGGACAAGAGTCGAGGCTGTCAGTGCAATTTCATAACTAGTTGGTGCGTTC
novel_mir173	TTGATAATACATCGTCATATGTA	ACAGAATCTCATACAGGTTGAGCAGATGATGGATGAAGCAGTGTTGAGCAATAGGAATATTGTCGCTGAAAGATTTATACCTTGATAATACATCGTCATATGTACATATCACATGTTTTCCGCTGCAAGAACTTAAGTTATGTACTAAAGTCCAAGAACATGCTTGTCATATGAATTACGGCTTTTGTTAAGGAGAATTCCCCTGTTCAATTATACCTGTTTAGAGTTACAATCCCTTCTAAATCATACTTAGGTTTGAGGGGATGTGACAAATGTACTGC

novel_mir174	CAGATCCGGATCGGACCGGACCAT	CTTAGGCTTGTCAATAAACAAGTTTTAATCGGGACTAGAACCAATAAATAAGTATTGGTTCTGGTTCTGAAATCAGAACCAGATCCGGATCGGACCGGACCATCAAACTATGGATCTAAACGGTTCGGGTCTAAAACGAGTCTGAA
novel_mir175	ATGCAAAAAAGAAACATGTCA	ATACTCCCTCCGTCCCAATATTCATGTCACGTTTTCCTTTTTGGGCTGTCCCATTAAGTTGGTCCGTTTCTAAAAAAGGAAACACTTTTTACACTAAAAATTACCTCGAATTTCGTGTCATCCACTACTTTACACACAAAACACAACTCTCTTTAATCTACAATCTACATGCAAAAAAGAAACATGTCATGAATATTGGGACGGAGGGAGTAC
novel_mir176	GGTTAATGCCCGAAGCCAA	AGCCCACATCTTCATCGTCATTACATTTGTGTGTGTTGGCCTAGTGATAGAGTGGTTAATGCCCGAAGCCAAAGGTCTCGGGTTCGAGTCCACCGTGACGCAGCCTTTAAAAATTTCTGTGCTGCTCCTATTTTGTGGGCG
novel_mir177	TCTTAAGCGGTCCGGATCTGA	ATGGTTTTGATTCTGGAACCGATCCATTAGACACTGGATCCGGACCGGATCCGGACCATCAACTATGGGTCTTAAGCGGTCCGGATCTGAGCGGATCTGGAACCAGAACTGGAACCAA
novel_mir178	ATTCGATGTATTTTTTTGTATAAAAATA	GTAGTACTCCCTCCCTCCCAATAATACCCCGTCCTAATAATGATGGTTACTTTTTTTTGTCCCAATAATGATAGTTACTCTTTTTTACACGTAAATTAAGGAGAGTTGTGTTTTGTGTGCAAATTAGTTGGTAACATGAAATTCGATGTATTTTTTTGTATAAAAATATTTCATTTTTTGGAAACATATCTACTTAATGGGACAATTCAAAAAAGAAAAGGTACCAACAAAAATGGGACGAAGGGAGTATTAA
novel_mir179	GTGTGTGTGTGTTGGCCTAGTGGTAGAGT	ATTGGTGTGTGTGTGTTGGCCTAGTGGTAGAGTGGTTAATGCCCGAGGCTAAAGGTCTCGGGTTCGAGTCCACCGTGACGCGGCCTTTAAAAATTTCTGTATTTAATTGATCAAAAAAAAGAATAAATGGGAGATTTGAGCACCCCTACTTAACCAACACCCCTACTTAACCAAC

novel_mir180	GTCTGTTTGGTAGCTCTGTTTTG	TGTTTGGTAGCTCTGTTTCGTATCCTTACGGCCCGTGTTTTAATGACGGGCTTGGTGGTTTTGTCTGTTTGGTAGCTCTGTTTTGCACTATTTCTTTAGGATTTGTAACCTACCTAAATGGTGGGTTACGAAACCTAACTGCACAGTACATTTTAACTAGACGAAACCTAACTAGACGAAACCTAACTAACCTACCAAACG
novel_mir181	TCTCACTGGATCCAACCCCTATATATATAT	CGGTGCATTAGTTTTATACATAAAATGCATTAATTTTATACTTAAATGCATTGGTTTTTAGGTTTTTACGAAAAAATAGGTTCTTATTATAACTCGACCACATATATATATATATATAGGGGTTGGATCAAGTGAGAACTCATCTTTTTTGAGAACTTGAGAACCATGCATATGACGTTGAAGATAATGCACAAAATAGTATAAAATTGTGCATAACGAAAACTTTCTAAAATTGTTGCACAACAAAATAATTTTAGAAAGTTTTCGTTATGCACAACTTTATACCGTTTTGTGCATCATCTTCAACGTCTTATGCATGGTTCTCAAGTTCTCAAAAAAGATGAGTTCTCACTGGATCCAACCCCTATATATATATATATATATATATATATATATATAGGGGTTGGATCCAGTGAGAACTACATCTTTTTTGCGAACTCTGCGAACCATGCATAAGACGTGTAAAGATGATGCACAAAACGGTATAAAGTTGTGCACAACGAAAACTTTCTAAAATTGTTTTGTTGTGCAACAATTTTAGAATGTTTTCGTTGTGCACAACTTTATAGATTTTATTCATCAACTTTATACGTCTGTGCATAGTTCTCAAGTTCTCAAAAAAGATGAGTTCTCACTTGATCCAACCCCTATATATATATATATATGTGGTCGAGTTATAATAAGAACCTATTTTTTCGTAAAAACCTAAAAACCAATGCATTTAAGTATAAAATTAATGCATTTTATGTATAAAACTAATGCACCC

Table S18. known miRNA members in each family in rosemary

miRNAs	Number	Family members
miR6300	1	miR6300
miR6173	1	miR6173
miR5658	1	miR5658
miR5141	1	miR5141
miR1171	1	miR1171
miR858	1	miR858b
miR845	1	miR845d
miR530	1	miR530a
miR408	4	miR408d、miR408b-5p_2、miR408-5p_8、miR408-3p_2
miR399	3	miR399b、miR399a_6、miR399_1
miR398	2	miR398b、miR398a-3p
miR397	3	miR397a_3、miR397-5p_1、miR397-5p
miR396	7	miR396h、miR396g-3p、miR396b、miR396a-5p、miR396a-3p_5、miR396a-3p_4、miR396a-3p_1
miR172	1	miR172a_4
miR171	8	miR171n-5p、miR171f_3、miR171d-5p_2、miR171d_1、miR171c_3、miR171b-3p_3、miR171b-3p、miR171a-3p_1
miR169	3	miR169m、miR169e_3、miR169e_2
miR168	6	miR168b_1、miR168a-5p、miR168a-3p、miR168-5p、miR168-3p、miR168
miR167	11	miR167d-5p、miR167d_1、miR167d、miR166u、miR166m_2、miR166h-3p_1、miR166h-3p、miR166e-3p、miR166e、miR166a-3p、miR166a
miR160	4	miR160h_1、miR160b_1、miR160a-5p、miR160
miR159	3	miR159c_5、miR159a_1、miR156q
miR156	6	miR156k_2、miR156c、miR156b_2、miR156a-5p、miR156a_2、miR156a

Table S19. Network of MeJA-responsive miRNAs and their targets

CKvsM10			CKvsM50			CKvsM100		
**miRNA id**	**type**	**Target id**	**miRNA id**	**type**	**Target id**	**miRNA id**	**type**	**Target id**
miR396a-3p_4	Up	CL5140.Contig8_All	novel_mir97	Up	CL11756.Contig7_All	miR396a-3p_4	Up	CL5140.Contig8_All
miR396a-3p_4	Up	CL5140.Contig4_All	novel_mir97	Up	Unigene12426_All	miR396a-3p_4	Up	CL5140.Contig4_All
miR396a-3p_4	Up	CL5140.Contig1_All	novel_mir97	Up	CL8986.Contig17_All	miR396a-3p_4	Up	CL5140.Contig1_All
miR396a-3p_4	Up	Unigene13456_All	novel_mir97	Up	Unigene11539_All	miR396a-3p_4	Up	Unigene13456_All
miR396a-3p_4	Up	CL10745.Contig5_All	novel_mir97	Up	CL11756.Contig1_All	miR396a-3p_4	Up	CL10745.Contig5_All
miR396a-3p_4	Up	CL2219.Contig7_All	novel_mir97	Up	Unigene9326_All	miR396a-3p_4	Up	CL2219.Contig7_All
miR396a-3p_4	Up	CL5140.Contig7_All	novel_mir97	Up	CL11930.Contig3_All	miR396a-3p_4	Up	CL5140.Contig7_All
miR396a-3p_4	Up	CL5140.Contig3_All	miR160	Up	Unigene22926_All	miR396a-3p_4	Up	CL5140.Contig3_All
miR396a-3p_4	Up	CL6647.Contig4_All	miR160	Up	CL11742.Contig5_All	miR396a-3p_4	Up	CL6647.Contig4_All
miR396a-3p_4	Up	CL2219.Contig4_All	miR396a-5p	Up	CL623.Contig3_All	miR396a-3p_4	Up	CL2219.Contig4_All
miR396a-3p_4	Up	Unigene25309_All	miR396a-5p	Up	CL623.Contig1_All	miR396a-3p_4	Up	Unigene25309_All
miR396a-3p_4	Up	CL10745.Contig4_All	miR396a-5p	Up	CL82.Contig3_All	miR396a-3p_4	Up	CL10745.Contig4_All
miR396a-3p_4	Up	CL11248.Contig15_All	miR396a-5p	Up	Unigene15827_All	miR396a-3p_4	Up	CL11248.Contig15_All
miR396a-3p_4	Up	Unigene26657_All	miR396a-5p	Up	Unigene13456_All	miR396a-3p_4	Up	Unigene26657_All
miR396a-3p_4	Up	CL10745.Contig7_All	miR396a-5p	Up	Unigene35300_All	miR396a-3p_4	Up	CL10745.Contig7_All
miR396a-3p_4	Up	CL7970.Contig2_All	miR396a-5p	Up	CL2267.Contig9_All	miR396a-3p_4	Up	CL7970.Contig2_All
miR396a-3p_4	Up	CL7970.Contig1_All	miR396a-5p	Up	Unigene565_All	miR396a-3p_4	Up	CL7970.Contig1_All
miR396a-3p_4	Up	CL10745.Contig2_All	miR396a-5p	Up	CL2267.Contig1_All	miR396a-3p_4	Up	CL10745.Contig2_All
miR396a-3p_4	Up	CL2219.Contig9_All	miR396a-5p	Up	CL7970.Contig3_All	miR396a-3p_4	Up	CL2219.Contig9_All
miR396a-3p_4	Up	CL12157.Contig3_All	miR396a-5p	Up	CL10458.Contig4_All	miR396a-3p_4	Up	CL12157.Contig3_All
miR396a-3p_4	Up	CL12157.Contig2_All	miR396a-5p	Up	CL6693.Contig6_All	miR396a-3p_4	Up	CL12157.Contig2_All
miR396a-3p_4	Up	CL10745.Contig8_All	miR396a-5p	Up	CL7970.Contig1_All	miR396a-3p_4	Up	CL10745.Contig8_All
novel_mir97	Up	CL11756.Contig7_All	miR396a-5p	Up	CL7970.Contig2_All	novel_mir97	Up	CL11756.Contig7_All
novel_mir97	Up	Unigene12426_All	miR396a-5p	Up	CL623.Contig2_All	novel_mir97	Up	Unigene12426_All

**novel_mir97**	**Up**	**CL8986.Contig17_All**	**miR396a-5p**	**Up**	**CL82.Contig1_All**	**novel_mir97**	**Up**	**CL8986.Contig17_All**
**novel_mir97**	**Up**	**Unigene11539_All**	**miR166e-3p**	**Up**	**Unigene23840_All**	**novel_mir97**	**Up**	**Unigene11539_All**
**novel_mir97**	**Up**	**CL11756.Contig1_All**	**miR166e-3p**	**Up**	**CL252.Contig8_All**	**novel_mir97**	**Up**	**CL11756.Contig1_All**
**novel_mir97**	**Up**	**Unigene9326_All**	**miR166e-3p**	**Up**	**CL252.Contig7_All**	**novel_mir97**	**Up**	**Unigene9326_All**
**novel_mir97**	**Up**	**CL11930.Contig3_All**	**miR166e-3p**	**Up**	**CL252.Contig9_All**	**novel_mir97**	**Up**	**CL11930.Contig3_All**
**novel_mir106**	**Up**	**CL1290.Contig17_All**	**miR166e-3p**	**Up**	**CL127.Contig8_All**	**miR160**	**Up**	**Unigene22926_All**
**novel_mir106**	**Up**	**Unigene5040_All**	**miR166e-3p**	**Up**	**CL127.Contig9_All**	**miR160**	**Up**	**CL11742.Contig5_All**
**novel_mir106**	**Up**	**Unigene3477_All**	**miR166e-3p**	**Up**	**Unigene19733_All**	**miR166e-3p**	**Up**	**Unigene23840_All**
**novel_mir106**	**Up**	**CL12266.Contig2_All**	**miR166e-3p**	**Up**	**Unigene23833_All**	**miR166e-3p**	**Up**	**CL252.Contig8_All**
**novel_mir106**	**Up**	**Unigene6451_All**	**miR166e-3p**	**Up**	**Unigene23835_All**	**miR166e-3p**	**Up**	**CL252.Contig7_All**
**novel_mir106**	**Up**	**CL1290.Contig42_All**	**miR166e-3p**	**Up**	**CL127.Contig13_All**	**miR166e-3p**	**Up**	**CL252.Contig9_All**
**novel_mir106**	**Up**	**Unigene12079_All**	**miR166e-3p**	**Up**	**Unigene23839_All**	**miR166e-3p**	**Up**	**CL127.Contig8_All**
**novel_mir106**	**Up**	**CL222.Contig47_All**	**miR166e-3p**	**Up**	**CL127.Contig7_All**	**miR166e-3p**	**Up**	**CL127.Contig9_All**
**novel_mir106**	**Up**	**Unigene7755_All**	**miR166e-3p**	**Up**	**Unigene19729_All**	**miR166e-3p**	**Up**	**Unigene19733_All**
**novel_mir106**	**Up**	**CL1925.Contig3_All**	**miR166e-3p**	**Up**	**CL127.Contig6_All**	**miR166e-3p**	**Up**	**Unigene23833_All**
**novel_mir106**	**Up**	**Unigene30000_All**	**miR166e-3p**	**Up**	**CL127.Contig1_All**	**miR166e-3p**	**Up**	**Unigene23835_All**
**novel_mir106**	**Up**	**CL994.Contig6_All**	**miR166e-3p**	**Up**	**Unigene23829_All**	**miR166e-3p**	**Up**	**CL127.Contig13_All**
**novel_mir106**	**Up**	**Unigene6866_All**	**miR166e-3p**	**Up**	**CL127.Contig12_All**	**miR166e-3p**	**Up**	**Unigene23839_All**
**novel_mir106**	**Up**	**CL994.Contig5_All**	**miR166e-3p**	**Up**	**CL127.Contig11_All**	**miR166e-3p**	**Up**	**CL127.Contig7_All**
**novel_mir106**	**Up**	**CL1925.Contig1_All**	**miR166e-3p**	**Up**	**CL11080.Contig3_All**	**miR166e-3p**	**Up**	**Unigene19729_All**
**novel_mir106**	**Up**	**Unigene6520_All**	**miR171b-3p**	**Up**	**CL3157.Contig1_All**	**miR166e-3p**	**Up**	**CL127.Contig6_All**
**novel_mir106**	**Up**	**CL12266.Contig1_All**	**miR171b-3p**	**Up**	**CL3157.Contig4_All**	**miR166e-3p**	**Up**	**CL127.Contig1_All**
**miR396a-5p**	**Up**	**CL623.Contig3_All**	**miR171b-3p**	**Up**	**CL1203.Contig7_All**	**miR166e-3p**	**Up**	**Unigene23829_All**
**miR396a-5p**	**Up**	**CL623.Contig1_All**	**miR399b**	**Up**	**CL2276.Contig2_All**	**miR166e-3p**	**Up**	**CL127.Contig12_All**
**miR396a-5p**	**Up**	**CL82.Contig3_All**	**miR399b**	**Up**	**Unigene8906_All**	**miR166e-3p**	**Up**	**CL127.Contig11_All**
**miR396a-5p**	**Up**	**Unigene15827_All**	**miR399b**	**Up**	**CL2276.Contig5_All**	**miR166e-3p**	**Up**	**CL11080.Contig3_All**
**miR396a-5p**	**Up**	**Unigene13456_All**	**miR399b**	**Up**	**CL2276.Contig1_All**	**miR171b-3p**	**Up**	**CL3157.Contig1_All**
**miR396a-5p**	**Up**	**Unigene35300_All**	**miR399b**	**Up**	**CL3187.Contig4_All**	**miR171b-3p**	**Up**	**CL3157.Contig4_All**

miR396a-5p	Up	CL2267.Contig9_All	miR399b	Up	CL2276.Contig8_All	miR171b-3p	Up	CL1203.Contig7_All
miR396a-5p	Up	Unigene565_All	miR399b	Up	CL2276.Contig6_All	miR399b	Up	CL2276.Contig2_All
miR396a-5p	Up	CL2267.Contig1_All	miR399b	Up	CL11662.Contig2_All	miR399b	Up	Unigene8906_All
miR396a-5p	Up	CL7970.Contig3_All	miR399b	Up	CL3187.Contig6_All	miR399b	Up	CL2276.Contig5_All
miR396a-5p	Up	CL10458.Contig4_All	miR399b	Up	CL3187.Contig8_All	miR399b	Up	CL2276.Contig1_All
miR396a-5p	Up	CL6693.Contig6_All	miR399b	Up	CL2276.Contig7_All	miR399b	Up	CL3187.Contig4_All
miR396a-5p	Up	CL7970.Contig1_All	miR399a_6	Up	CL2276.Contig2_All	miR399b	Up	CL2276.Contig8_All
miR396a-5p	Up	CL7970.Contig2_All	miR399a_6	Up	CL2276.Contig5_All	miR399b	Up	CL2276.Contig6_All
miR396a-5p	Up	CL623.Contig2_All	miR399a_6	Up	CL2276.Contig1_All	miR399b	Up	CL11662.Contig2_All
miR396a-5p	Up	CL82.Contig1_All	miR399a_6	Up	CL3215.Contig5_All	miR399b	Up	CL3187.Contig6_All
novel_mir120	Up	CL5776.Contig3_All	miR399a_6	Up	CL2276.Contig8_All	miR399b	Up	CL3187.Contig8_All
novel_mir120	Up	CL5776.Contig2_All	miR399a_6	Up	CL858.Contig3_All	miR399b	Up	CL2276.Contig7_All
novel_mir120	Up	CL6074.Contig2_All	miR399a_6	Up	CL2276.Contig6_All	miR399a_6	Up	CL2276.Contig2_All
novel_mir120	Up	CL6074.Contig7_All	miR399a_6	Up	CL3215.Contig3_All	miR399a_6	Up	CL2276.Contig5_All
novel_mir120	Up	CL6074.Contig5_All	miR399a_6	Up	CL3381.Contig1_All	miR399a_6	Up	CL2276.Contig1_All
novel_mir120	Up	CL6074.Contig10_All	miR399a_6	Up	CL11662.Contig2_All	miR399a_6	Up	CL3215.Contig5_All
novel_mir120	Up	CL6074.Contig4_All	miR399a_6	Up	CL2276.Contig7_All	miR399a_6	Up	CL2276.Contig8_All
novel_mir120	Up	CL957.Contig5_All	miR399a_6	Up	CL3215.Contig2_All	miR399a_6	Up	CL858.Contig3_All
novel_mir120	Up	CL11242.Contig3_All	miR399a_6	Up	CL3215.Contig4_All	miR399a_6	Up	CL2276.Contig6_All
novel_mir120	Up	CL5783.Contig2_All	miR168b_1	Up	Unigene35241_All	miR399a_6	Up	CL3215.Contig3_All
novel_mir120	Up	CL6074.Contig6_All	miR168b_1	Up	Unigene13184_All	miR399a_6	Up	CL3381.Contig1_All
novel_mir120	Up	CL957.Contig1_All	miR168b_1	Up	CL462.Contig7_All	miR399a_6	Up	CL11662.Contig2_All
novel_mir120	Up	CL6074.Contig11_All	miR168b_1	Up	CL725.Contig2_All	miR399a_6	Up	CL2276.Contig7_All
novel_mir120	Up	Unigene20529_All	miR168b_1	Up	Unigene3288_All	miR399a_6	Up	CL3215.Contig2_All
novel_mir120	Up	CL6074.Contig3_All	miR168b_1	Up	Unigene3262_All	miR399a_6	Up	CL3215.Contig4_All
novel_mir120	Up	CL5776.Contig5_All	miR168b_1	Up	CL4078.Contig3_All	novel_mir47	Up	Unigene11437_All
novel_mir120	Up	CL5776.Contig4_All	miR168b_1	Up	CL1134.Contig2_All	novel_mir47	Up	Unigene2403_All
novel_mir47	Up	Unigene11437_All	miR168b_1	Up	CL4078.Contig1_All	novel_mir47	Up	Unigene4672_All

novel_mir47	Up	Unigene2403_All	miR168b_1	Up	CL10842.Contig4_All	novel_mir47	Up	Unigene9755_All
novel_mir47	Up	Unigene4672_All	miR168b_1	Up	Unigene3326_All	novel_mir47	Up	CL1485.Contig4_All
novel_mir47	Up	Unigene9755_All	miR168b_1	Up	Unigene26983_All	novel_mir47	Up	Unigene7432_All
novel_mir47	Up	CL1485.Contig4_All	miR168b_1	Up	CL725.Contig49_All	novel_mir47	Up	CL11930.Contig5_All
novel_mir47	Up	Unigene7432_All	miR168b_1	Up	Unigene12302_All	novel_mir47	Up	Unigene11850_All
novel_mir47	Up	CL11930.Contig5_All	miR168b_1	Up	Unigene26984_All	novel_mir47	Up	CL11930.Contig11_All
novel_mir47	Up	Unigene11850_All	miR168b_1	Up	Unigene26694_All	novel_mir72	Up	CL8205.Contig3_All
novel_mir47	Up	CL11930.Contig11_All	miR168b_1	Up	CL1134.Contig5_All	novel_mir72	Up	CL2494.Contig5_All
novel_mir72	Up	CL8205.Contig3_All	miR168b_1	Up	CL11386.Contig5_All	novel_mir72	Up	CL228.Contig2_All
novel_mir72	Up	CL2494.Contig5_All	miR168b_1	Up	CL1134.Contig3_All	novel_mir72	Up	CL7956.Contig1_All
novel_mir72	Up	CL228.Contig2_All	miR168b_1	Up	Unigene9352_All	novel_mir72	Up	Unigene2896_All
novel_mir72	Up	CL7956.Contig1_All	miR168b_1	Up	CL12178.Contig8_All	novel_mir72	Up	CL10111.Contig3_All
novel_mir72	Up	Unigene2896_All	miR168b_1	Up	CL462.Contig5_All	novel_mir72	Up	CL11367.Contig4_All
novel_mir72	Up	CL10111.Contig3_All	miR168b_1	Up	CL3109.Contig3_All	novel_mir72	Up	CL9944.Contig2_All
novel_mir72	Up	CL11367.Contig4_All	miR168b_1	Up	Unigene26982_All	novel_mir72	Up	Unigene19898_All
novel_mir72	Up	CL9944.Contig2_All	miR168b_1	Up	CL3109.Contig1_All	novel_mir72	Up	Unigene6418_All
novel_mir72	Up	Unigene19898_All	novel_mir72	Up	CL8205.Contig3_All	novel_mir72	Up	CL4372.Contig2_All
novel_mir72	Up	Unigene6418_All	novel_mir72	Up	CL2494.Contig5_All	novel_mir72	Up	Unigene3591_All
novel_mir72	Up	CL4372.Contig2_All	novel_mir72	Up	CL228.Contig2_All	novel_mir72	Up	CL10111.Contig1_All
novel_mir72	Up	Unigene3591_All	novel_mir72	Up	CL7956.Contig1_All	novel_mir72	Up	Unigene9592_All
novel_mir72	Up	CL10111.Contig1_All	novel_mir72	Up	Unigene2896_All	novel_mir72	Up	Unigene7472_All
novel_mir72	Up	Unigene9592_All	novel_mir72	Up	CL10111.Contig3_All	novel_mir72	Up	CL2494.Contig1_All
novel_mir72	Up	Unigene7472_All	novel_mir72	Up	CL11367.Contig4_All	novel_mir72	Up	Unigene30896_All
novel_mir72	Up	CL2494.Contig1_All	novel_mir72	Up	CL9944.Contig2_All	novel_mir72	Up	Unigene3832_All
novel_mir72	Up	Unigene3832_All	novel_mir72	Up	Unigene19898_All	novel_mir72	Up	CL2352.Contig11_All
novel_mir72	Up	CL2352.Contig11_All	novel_mir72	Up	Unigene6418_All	novel_mir72	Up	Unigene11537_All
novel_mir72	Up	Unigene11537_All	novel_mir72	Up	CL4372.Contig2_All	novel_mir72	Up	Unigene13829_All
novel_mir72	Up	Unigene13829_All	novel_mir72	Up	Unigene3591_All	novel_mir72	Up	CL2144.Contig6_All

novel_mir72	Up	CL2144.Contig6_All	novel_mir72	Up	CL10111.Contig1_All	novel_mir72	Up	CL9115.Contig1_All
novel_mir72	Up	CL9115.Contig1_All	novel_mir72	Up	Unigene9592_All	novel_mir72	Up	CL2144.Contig5_All
novel_mir72	Up	CL2144.Contig5_All	novel_mir72	Up	Unigene7472_All	novel_mir72	Up	CL8205.Contig4_All
novel_mir72	Up	CL8205.Contig4_All	novel_mir72	Up	CL2494.Contig1_All	novel_mir72	Up	Unigene10328_All
novel_mir72	Up	Unigene10328_All	novel_mir72	Up	Unigene30896_All	novel_mir72	Up	Unigene37588_All
novel_mir72	Up	Unigene37588_All	novel_mir72	Up	Unigene3832_All	novel_mir72	Up	CL7956.Contig6_All
novel_mir72	Up	CL7956.Contig6_All	novel_mir72	Up	CL2352.Contig11_All	novel_mir72	Up	CL5977.Contig2_All
novel_mir72	Up	CL5977.Contig2_All	novel_mir72	Up	Unigene11537_All	novel_mir72	Up	CL11367.Contig6_All
novel_mir72	Up	CL11367.Contig6_All	novel_mir72	Up	Unigene13829_All	novel_mir72	Up	Unigene4211_All
novel_mir72	Up	Unigene4211_All	novel_mir72	Up	CL2144.Contig6_All	novel_mir72	Up	Unigene24882_All
novel_mir72	Up	Unigene24882_All	novel_mir72	Up	CL9115.Contig1_All	novel_mir72	Up	CL10048.Contig1_All
novel_mir72	Up	CL10048.Contig1_All	novel_mir72	Up	CL2144.Contig5_All	novel_mir72	Up	Unigene9743_All
novel_mir72	Up	Unigene9743_All	novel_mir72	Up	CL8205.Contig4_All	novel_mir72	Up	CL11367.Contig7_All
novel_mir72	Up	CL11367.Contig7_All	novel_mir72	Up	Unigene10328_All	novel_mir72	Up	Unigene24406_All
novel_mir72	Up	Unigene24406_All	novel_mir72	Up	Unigene37588_All	novel_mir72	Up	CL10956.Contig2_All
novel_mir72	Up	CL10956.Contig2_All	novel_mir72	Up	CL7956.Contig6_All	novel_mir168	Up	CL601.Contig6_All
novel_mir140	Up	Unigene3543_All	novel_mir72	Up	CL5977.Contig2_All	novel_mir168	Up	CL11469.Contig2_All
novel_mir140	Up	CL11092.Contig9_All	novel_mir72	Up	CL11367.Contig6_All	novel_mir168	Up	CL7212.Contig3_All
novel_mir140	Up	Unigene6994_All	novel_mir72	Up	Unigene4211_All	novel_mir168	Up	Unigene14756_All
novel_mir140	Up	Unigene6703_All	novel_mir72	Up	Unigene24882_All	novel_mir168	Up	Unigene7928_All
novel_mir140	Up	Unigene11042_All	novel_mir72	Up	CL10048.Contig1_All	novel_mir168	Up	Unigene5920_All
novel_mir140	Up	Unigene10722_All	novel_mir72	Up	Unigene9743_All	novel_mir168	Up	CL11469.Contig1_All
novel_mir90	Up	Unigene6694_All	novel_mir72	Up	CL11367.Contig7_All	novel_mir168	Up	Unigene10278_All
novel_mir90	Up	Unigene37279_All	novel_mir72	Up	Unigene24406_All	novel_mir168	Up	Unigene2414_All
novel_mir90	Up	Unigene11054_All	novel_mir72	Up	CL10956.Contig2_All	novel_mir168	Up	CL9491.Contig1_All
novel_mir90	Up	Unigene16886_All	novel_mir140	Up	Unigene3543_All	novel_mir168	Up	CL9811.Contig2_All
novel_mir90	Up	CL1650.Contig2_All	novel_mir140	Up	CL11092.Contig9_All	novel_mir168	Up	CL11143.Contig7_All
novel_mir43	Up	Unigene20079_All	novel_mir140	Up	Unigene6994_All	miR171f_3	Up	CL3157.Contig1_All

novel_mir43	Up	CL3020.Contig4_All	novel_mir140	Up	Unigene6703_All	miR171f_3	Up	CL3157.Contig4_All
novel_mir43	Up	CL3020.Contig2_All	novel_mir140	Up	Unigene11042_All	novel_mir11	Up	CL11597.Contig3_All
miR156a-5p	Up	Unigene32383_All	novel_mir140	Up	Unigene10722_All	novel_mir11	Up	Unigene4441_All
miR156a-5p	Up	CL11653.Contig6_All	miR396a-3p_5	Up	CL5140.Contig8_All	miR168-3p	Up	CL4078.Contig1_All
miR156a-5p	Up	CL3247.Contig4_All	miR396a-3p_5	Up	CL3506.Contig7_All	miR168-3p	Up	CL4078.Contig3_All
miR156a-5p	Up	CL11653.Contig21_All	miR396a-3p_5	Up	CL5140.Contig4_All	novel_mir43	Up	Unigene20079_All
miR156a-5p	Up	CL11653.Contig20_All	miR396a-3p_5	Up	CL3506.Contig2_All	novel_mir43	Up	CL3020.Contig4_All
miR156a-5p	Up	CL5489.Contig5_All	miR396a-3p_5	Up	CL3025.Contig5_All	novel_mir43	Up	CL3020.Contig2_All
miR156a-5p	Up	CL11221.Contig3_All	miR396a-3p_5	Up	Unigene15223_All	novel_mir62	Up	CL71.Contig43_All
miR156a-5p	Up	CL11653.Contig7_All	miR396a-3p_5	Up	Unigene1142_All	novel_mir62	Up	Unigene3515_All
miR156a-5p	Up	CL11221.Contig1_All	miR396a-3p_5	Up	CL3506.Contig5_All	novel_mir62	Up	Unigene2336_All
miR156a-5p	Up	CL10499.Contig1_All	miR396a-3p_5	Up	CL5140.Contig3_All	novel_mir62	Up	Unigene8140_All
miR156a-5p	Up	CL11221.Contig2_All	miR396a-3p_5	Up	CL3025.Contig12_All	novel_mir62	Up	CL11634.Contig12_All
miR156a-5p	Up	Unigene20076_All	miR396a-3p_5	Up	Unigene26657_All	novel_mir62	Up	CL11827.Contig3_All
miR156a-5p	Up	CL4021.Contig2_All	miR396a-3p_5	Up	CL9790.Contig2_All	novel_mir62	Up	CL10474.Contig1_All
miR156a-5p	Up	CL4424.Contig7_All	miR396a-3p_5	Up	CL7970.Contig2_All	novel_mir62	Up	Unigene2580_All
miR156a-5p	Up	CL5489.Contig1_All	miR396a-3p_5	Up	CL7970.Contig1_All	novel_mir62	Up	CL12075.Contig17_All
miR156a-5p	Up	CL4553.Contig1_All	miR396a-3p_5	Up	Unigene32495_All	novel_mir62	Up	Unigene26594_All
miR156a-5p	Up	CL4803.Contig6_All	miR396a-3p_5	Up	CL1165.Contig17_All	novel_mir62	Up	Unigene4989_All
miR156a-5p	Up	CL2170.Contig2_All	miR396a-3p_5	Up	CL2219.Contig9_All	novel_mir62	Up	CL651.Contig9_All
miR156a-5p	Up	CL9049.Contig3_All	miR396a-3p_5	Up	CL9790.Contig1_All	novel_mir62	Up	Unigene34330_All
miR156a-5p	Up	CL2170.Contig8_All	miR396a-3p_5	Up	CL6402.Contig2_All	novel_mir62	Up	CL5073.Contig11_All
miR156a-5p	Up	CL2558.Contig12_All	miR396a-3p_5	Up	CL3025.Contig13_All	novel_mir62	Up	CL4469.Contig2_All
miR156a-5p	Up	CL8954.Contig3_All	miR396a-3p_5	Up	CL1165.Contig3_All	novel_mir62	Up	CL651.Contig3_All
miR156a-5p	Up	CL5489.Contig2_All	miR396a-3p_5	Up	CL3025.Contig1_All	novel_mir62	Up	Unigene7005_All
miR156a-5p	Up	CL11653.Contig2_All	miR396a-3p_5	Up	CL5140.Contig1_All	miR168-5p	Up	CL4078.Contig1_All
miR156a-5p	Up	CL2558.Contig11_All	miR396a-3p_5	Up	CL6402.Contig3_All	miR168-5p	Up	CL4078.Contig3_All
miR156a-5p	Up	CL5489.Contig4_All	miR396a-3p_5	Up	CL10745.Contig5_All	novel_mir170	Up	CL1071.Contig3_All

**miR156a-5p**	**Up**	**CL11653.Contig19_All**	**miR396a-3p_5**	**Up**	**Unigene13456_All**	**novel_mir170**	**Up**	**Unigene8115_All**
**miR156a-5p**	**Up**	**CL2170.Contig4_All**	**miR396a-3p_5**	**Up**	**CL3025.Contig7_All**	**novel_mir170**	**Up**	**CL1071.Contig4_All**
**miR156a-5p**	**Up**	**Unigene26237_All**	**miR396a-3p_5**	**Up**	**CL2219.Contig7_All**	**miR396a-3p_5**	**Up**	**CL5140.Contig8_All**
**miR156a-5p**	**Up**	**Unigene20170_All**	**miR396a-3p_5**	**Up**	**CL5140.Contig7_All**	**miR396a-3p_5**	**Up**	**CL3506.Contig7_All**
**miR156a-5p**	**Up**	**CL11653.Contig8_All**	**miR396a-3p_5**	**Up**	**CL2219.Contig4_All**	**miR396a-3p_5**	**Up**	**CL5140.Contig4_All**
**miR156a-5p**	**Up**	**CL3247.Contig2_All**	**miR396a-3p_5**	**Up**	**CL6647.Contig4_All**	**miR396a-3p_5**	**Up**	**CL3506.Contig2_All**
**miR156a-5p**	**Up**	**CL8954.Contig4_All**	**miR396a-3p_5**	**Up**	**CL10745.Contig2_All**	**miR396a-3p_5**	**Up**	**CL3025.Contig5_All**
**miR156a-5p**	**Up**	**CL2170.Contig6_All**	**miR396a-3p_5**	**Up**	**CL3506.Contig4_All**	**miR396a-3p_5**	**Up**	**Unigene15223_All**
**miR156a-5p**	**Up**	**CL8954.Contig1_All**	**miR396a-3p_5**	**Up**	**CL3025.Contig2_All**	**miR396a-3p_5**	**Up**	**Unigene1142_All**
**miR156a-5p**	**Up**	**Unigene185_All**	**novel_mir176**	**Up**	**Unigene5058_All**	**miR396a-3p_5**	**Up**	**CL3506.Contig5_All**
**miR156a-5p**	**Up**	**CL5489.Contig3_All**	**novel_mir176**	**Up**	**CL163.Contig3_All**	**miR396a-3p_5**	**Up**	**CL5140.Contig3_All**
**miR168**	**Up**	**Unigene13184_All**	**novel_mir176**	**Up**	**Unigene24445_All**	**miR396a-3p_5**	**Up**	**CL3025.Contig12_All**
**miR168**	**Up**	**CL462.Contig7_All**	**novel_mir176**	**Up**	**CL5614.Contig4_All**	**miR396a-3p_5**	**Up**	**Unigene26657_All**
**miR168**	**Up**	**CL11386.Contig5_All**	**novel_mir176**	**Up**	**Unigene32511_All**	**miR396a-3p_5**	**Up**	**CL9790.Contig2_All**
**miR168**	**Up**	**CL4078.Contig3_All**	**novel_mir176**	**Up**	**CL12253.Contig40_All**	**miR396a-3p_5**	**Up**	**CL7970.Contig2_All**
**miR168**	**Up**	**CL4078.Contig1_All**	**novel_mir176**	**Up**	**Unigene12217_All**	**miR396a-3p_5**	**Up**	**CL7970.Contig1_All**
**miR168**	**Up**	**Unigene26983_All**	**novel_mir176**	**Up**	**CL7229.Contig5_All**	**miR396a-3p_5**	**Up**	**Unigene32495_All**
**miR168**	**Up**	**CL462.Contig5_All**	**novel_mir176**	**Up**	**Unigene8781_All**	**miR396a-3p_5**	**Up**	**CL1165.Contig17_All**
**miR168**	**Up**	**Unigene26982_All**	**novel_mir176**	**Up**	**Unigene3801_All**	**miR396a-3p_5**	**Up**	**CL2219.Contig9_All**
**miR168**	**Up**	**Unigene26984_All**	**novel_mir176**	**Up**	**CL8468.Contig3_All**	**miR396a-3p_5**	**Up**	**CL9790.Contig1_All**
**miR168**	**Up**	**Unigene26694_All**	**novel_mir176**	**Up**	**CL163.Contig9_All**	**miR396a-3p_5**	**Up**	**CL6402.Contig2_All**
**miR168-5p**	**Up**	**CL4078.Contig1_All**	**novel_mir176**	**Up**	**CL163.Contig12_All**	**miR396a-3p_5**	**Up**	**CL3025.Contig13_All**
**miR168-5p**	**Up**	**CL4078.Contig3_All**	**novel_mir176**	**Up**	**CL3878.Contig8_All**	**miR396a-3p_5**	**Up**	**CL1165.Contig3_All**
**miR156a**	**Up**	**CL4803.Contig6_All**	**novel_mir176**	**Up**	**CL4883.Contig3_All**	**miR396a-3p_5**	**Up**	**CL3025.Contig1_All**
**miR156a**	**Up**	**CL9049.Contig3_All**	**novel_mir176**	**Up**	**Unigene9362_All**	**miR396a-3p_5**	**Up**	**CL5140.Contig1_All**
**miR156a**	**Up**	**CL2170.Contig2_All**	**novel_mir176**	**Up**	**CL5246.Contig1_All**	**miR396a-3p_5**	**Up**	**CL6402.Contig3_All**
**miR156a**	**Up**	**CL2170.Contig8_All**	**novel_mir176**	**Up**	**Unigene9623_All**	**miR396a-3p_5**	**Up**	**CL10745.Contig5_All**
**miR156a**	**Up**	**CL11653.Contig6_All**	**novel_mir176**	**Up**	**CL6239.Contig4_All**	**miR396a-3p_5**	**Up**	**Unigene13456_All**

miR156a	Up	CL3247.Contig4_All	novel_mir176	Up	CL12253.Contig20_All	miR396a-3p_5	Up	CL3025.Contig7_All
miR156a	Up	CL5489.Contig2_All	novel_mir176	Up	Unigene3363_All	miR396a-3p_5	Up	CL2219.Contig7_All
miR156a	Up	CL11653.Contig21_All	novel_mir176	Up	CL9876.Contig18_All	miR396a-3p_5	Up	CL5140.Contig7_All
miR156a	Up	CL11653.Contig2_All	novel_mir176	Up	CL9876.Contig16_All	miR396a-3p_5	Up	CL2219.Contig4_All
miR156a	Up	CL5489.Contig4_All	novel_mir176	Up	Unigene12107_All	miR396a-3p_5	Up	CL6647.Contig4_All
miR156a	Up	CL11653.Contig20_All	novel_mir176	Up	CL7229.Contig3_All	miR396a-3p_5	Up	CL10745.Contig2_All
miR156a	Up	CL5489.Contig5_All	novel_mir176	Up	CL9876.Contig14_All	miR396a-3p_5	Up	CL3506.Contig4_All
miR156a	Up	CL2170.Contig4_All	novel_mir176	Up	CL1212.Contig4_All	miR396a-3p_5	Up	CL3025.Contig2_All
miR156a	Up	CL11653.Contig19_All	novel_mir176	Up	Unigene3858_All	novel_mir101	Up	Unigene7460_All
miR156a	Up	CL11653.Contig7_All	novel_mir176	Up	CL163.Contig10_All	novel_mir101	Up	Unigene9709_All
miR156a	Up	CL11221.Contig1_All	novel_mir176	Up	Unigene4733_All	novel_mir101	Up	Unigene7468_All
miR156a	Up	Unigene20170_All	novel_mir176	Up	CL9876.Contig2_All	novel_mir101	Up	Unigene8657_All
miR156a	Up	CL11653.Contig8_All	novel_mir176	Up	CL6668.Contig11_All	novel_mir101	Up	Unigene10161_All
miR156a	Up	CL3247.Contig2_All	novel_mir176	Up	Unigene4444_All	novel_mir101	Up	Unigene7152_All
miR156a	Up	CL12256.Contig7_All	novel_mir176	Up	CL9876.Contig13_All	novel_mir101	Up	CL7502.Contig7_All
miR156a	Up	Unigene7368_All	novel_mir176	Up	CL3878.Contig13_All	novel_mir101	Up	Unigene3049_All
miR156a	Up	Unigene20076_All	novel_mir176	Up	Unigene6545_All	novel_mir101	Up	Unigene35289_All
miR156a	Up	CL12256.Contig12_All	novel_mir176	Up	CL9876.Contig17_All	novel_mir101	Up	CL11827.Contig8_All
miR156a	Up	CL5489.Contig1_All	novel_mir176	Up	CL10420.Contig2_All	novel_mir101	Up	Unigene4309_All
miR156a	Up	CL2170.Contig6_All	novel_mir176	Up	Unigene5756_All	novel_mir101	Up	Unigene2941_All
miR156a	Up	CL5489.Contig3_All	novel_mir176	Up	CL1212.Contig1_All	novel_mir101	Up	Unigene9455_All
novel_mir36	Down	Unigene30000_All	novel_mir176	Up	Unigene8658_All	novel_mir101	Up	Unigene3540_All
novel_mir36	Down	Unigene5040_All	novel_mir176	Up	Unigene2668_All	novel_mir101	Up	Unigene10331_All
novel_mir36	Down	CL994.Contig6_All	novel_mir176	Up	CL12323.Contig2_All	novel_mir101	Up	CL8130.Contig17_All
novel_mir36	Down	Unigene3477_All	novel_mir176	Up	CL11961.Contig11_All	novel_mir101	Up	Unigene10734_All
novel_mir36	Down	CL12266.Contig2_All	novel_mir176	Up	Unigene4107_All	novel_mir101	Up	Unigene11794_All
novel_mir36	Down	Unigene6866_All	novel_mir176	Up	CL163.Contig4_All	novel_mir101	Up	Unigene3531_All
novel_mir36	Down	CL994.Contig5_All	novel_mir176	Up	Unigene2556_All	novel_mir101	Up	Unigene4821_All

novel_mir36	Down	CL222.Contig47_All	novel_mir176	Up	Unigene2465_All	novel_mir101	Up	Unigene9547_All
novel_mir36	Down	Unigene6520_All	novel_mir176	Up	CL6668.Contig7_All	novel_mir101	Up	Unigene10661_All
novel_mir36	Down	Unigene11066_All	novel_mir176	Up	CL8468.Contig1_All	novel_mir101	Up	Unigene5826_All
novel_mir36	Down	CL12266.Contig1_All	novel_mir176	Up	CL10704.Contig2_All	novel_mir101	Up	Unigene8149_All
novel_mir161	Down	Unigene5846_All	novel_mir176	Up	CL11123.Contig1_All	novel_mir101	Up	Unigene9779_All
novel_mir161	Down	CL5937.Contig42_All	novel_mir176	Up	CL1615.Contig4_All	novel_mir101	Up	Unigene11497_All
novel_mir161	Down	CL5937.Contig55_All	novel_mir176	Up	CL4883.Contig2_All	miR168a-5p	Up	CL4078.Contig1_All
novel_mir161	Down	CL5290.Contig2_All	novel_mir176	Up	Unigene10699_All	miR168a-5p	Up	CL4078.Contig3_All
novel_mir161	Down	CL6981.Contig1_All	novel_mir176	Up	CL9876.Contig8_All	miR160a-5p	Down	CL8053.Contig2_All
novel_mir161	Down	CL3265.Contig7_All	novel_mir176	Up	Unigene6405_All	miR160a-5p	Down	CL11742.Contig1_All
novel_mir161	Down	Unigene13971_All	novel_mir176	Up	CL163.Contig20_All	miR160a-5p	Down	CL11742.Contig4_All
novel_mir161	Down	CL5937.Contig57_All	novel_mir176	Up	Unigene3222_All	miR160a-5p	Down	CL11742.Contig3_All
novel_mir161	Down	Unigene5995_All	novel_mir176	Up	Unigene10001_All	miR160a-5p	Down	CL8053.Contig3_All
novel_mir161	Down	Unigene9188_All	novel_mir176	Up	CL7229.Contig6_All	miR160a-5p	Down	CL8053.Contig1_All
novel_mir161	Down	Unigene9982_All	novel_mir176	Up	Unigene7911_All	miR160a-5p	Down	CL11742.Contig2_All
novel_mir161	Down	CL12079.Contig4_All	novel_mir176	Up	CL12253.Contig4_All	miR160a-5p	Down	CL7275.Contig1_All
novel_mir161	Down	Unigene11533_All	novel_mir176	Up	Unigene6459_All	miR160a-5p	Down	CL7275.Contig2_All
novel_mir161	Down	CL12282.Contig3_All	novel_mir176	Up	Unigene15439_All	miR167d_1	Down	CL1264.Contig9_All
novel_mir161	Down	Unigene5935_All	novel_mir176	Up	CL746.Contig32_All	miR167d_1	Down	CL10446.Contig1_All
novel_mir161	Down	CL5937.Contig76_All	novel_mir176	Up	CL1277.Contig4_All	miR167d_1	Down	Unigene9900_All
novel_mir161	Down	CL10762.Contig2_All	novel_mir176	Up	CL1212.Contig2_All	miR167d_1	Down	CL1264.Contig7_All
novel_mir161	Down	CL5937.Contig2_All	novel_mir176	Up	CL3878.Contig4_All	miR167d_1	Down	CL1264.Contig1_All
novel_mir67	Down	CL7599.Contig11_All	novel_mir176	Up	Unigene11990_All	miR167d_1	Down	CL10446.Contig22_All
novel_mir67	Down	Unigene11166_All	novel_mir176	Up	Unigene37603_All	miR167d_1	Down	CL1085.Contig12_All
novel_mir67	Down	CL7599.Contig1_All	novel_mir176	Up	CL40.Contig26_All	miR167d_1	Down	CL7983.Contig2_All
novel_mir67	Down	Unigene10773_All	novel_mir176	Up	Unigene10704_All	miR167d_1	Down	CL1264.Contig10_All
novel_mir67	Down	CL11385.Contig2_All	novel_mir176	Up	CL5614.Contig2_All	miR167d_1	Down	CL1264.Contig3_All
novel_mir67	Down	CL7599.Contig12_All	novel_mir176	Up	CL8468.Contig2_All	miR167d_1	Down	CL5786.Contig2_All

miR167d_1	Down	CL1264.Contig9_All	novel_mir176	Up	Unigene6781_All	miR167d_1	Down	CL1264.Contig8_All
miR167d_1	Down	CL10446.Contig1_All	novel_mir176	Up	Unigene4272_All	miR167d_1	Down	CL1264.Contig11_All
miR167d_1	Down	Unigene9900_All	novel_mir176	Up	Unigene36819_All	miR167d_1	Down	Unigene16296_All
miR167d_1	Down	CL1264.Contig7_All	novel_mir176	Up	Unigene10799_All	miR167d_1	Down	CL5786.Contig8_All
miR167d_1	Down	CL1264.Contig1_All	novel_mir176	Up	CL2520.Contig1_All	miR167d_1	Down	Unigene2643_All
miR167d_1	Down	CL10446.Contig22_All	novel_mir176	Up	CL1309.Contig2_All	miR167d_1	Down	CL1264.Contig4_All
miR167d_1	Down	CL1085.Contig12_All	novel_mir176	Up	CL10443.Contig3_All	miR167d_1	Down	Unigene8353_All
miR167d_1	Down	CL7983.Contig2_All	novel_mir176	Up	Unigene5877_All	miR167d_1	Down	CL3833.Contig1_All
miR167d_1	Down	CL1264.Contig10_All	novel_mir176	Up	Unigene4275_All	miR167d_1	Down	CL5786.Contig1_All
miR167d_1	Down	CL1264.Contig3_All	novel_mir176	Up	Unigene30367_All	miR167d_1	Down	CL5786.Contig7_All
miR167d_1	Down	CL5786.Contig2_All	novel_mir176	Up	CL12116.Contig8_All	miR167d_1	Down	Unigene12094_All
miR167d_1	Down	CL1264.Contig8_All	novel_mir176	Up	CL12106.Contig7_All	miR167d_1	Down	CL5786.Contig3_All
miR167d_1	Down	CL1264.Contig11_All	novel_mir176	Up	CL1277.Contig2_All	miR167d_1	Down	CL1085.Contig8_All
miR167d_1	Down	Unigene16296_All	novel_mir176	Up	CL11400.Contig1_All	miR167d_1	Down	CL10446.Contig9_All
miR167d_1	Down	CL5786.Contig8_All	novel_mir176	Up	Unigene3835_All	miR167d_1	Down	CL10446.Contig19_All
miR167d_1	Down	Unigene2643_All	novel_mir176	Up	CL163.Contig1_All	miR167d_1	Down	CL10446.Contig13_All
miR167d_1	Down	CL1264.Contig4_All	novel_mir176	Up	CL463.Contig6_All	miR167d_1	Down	CL1264.Contig5_All
miR167d_1	Down	Unigene8353_All	novel_mir176	Up	Unigene9281_All	miR167d_1	Down	CL1264.Contig2_All
miR167d_1	Down	CL3833.Contig1_All	novel_mir176	Up	CL6757.Contig1_All	miR167d_1	Down	CL10446.Contig4_All
miR167d_1	Down	CL5786.Contig1_All	novel_mir176	Up	Unigene3776_All	miR167d_1	Down	CL1264.Contig12_All
miR167d_1	Down	CL5786.Contig7_All	novel_mir176	Up	CL11400.Contig7_All	miR167d_1	Down	CL10446.Contig18_All
miR167d_1	Down	Unigene12094_All	novel_mir43	Up	Unigene20079_All	novel_mir67	Down	CL7599.Contig11_All
miR167d_1	Down	CL5786.Contig3_All	novel_mir43	Up	CL3020.Contig4_All	novel_mir67	Down	Unigene11166_All
miR167d_1	Down	CL1085.Contig8_All	novel_mir43	Up	CL3020.Contig2_All	novel_mir67	Down	CL7599.Contig1_All
miR167d_1	Down	CL10446.Contig9_All	novel_mir61	Up	CL11652.Contig33_All	novel_mir67	Down	Unigene10773_All
miR167d_1	Down	CL10446.Contig19_All	novel_mir61	Up	Unigene4832_All	novel_mir67	Down	CL11385.Contig2_All
miR167d_1	Down	CL10446.Contig13_All	novel_mir61	Up	CL11652.Contig1_All	novel_mir67	Down	CL7599.Contig12_All
miR167d_1	Down	CL1264.Contig5_All	novel_mir61	Up	CL11652.Contig29_All	miR166h-3p	Down	Unigene23840_All

miR167d_1	Down	CL1264.Contig2_All	novel_mir61	Up	Unigene8810_All	miR166h-3p	Down	CL5531.Contig2_All
miR167d_1	Down	CL10446.Contig4_All	novel_mir61	Up	CL11652.Contig7_All	miR166h-3p	Down	CL252.Contig8_All
miR167d_1	Down	CL1264.Contig12_All	novel_mir61	Up	CL6502.Contig1_All	miR166h-3p	Down	CL252.Contig7_All
miR167d_1	Down	CL10446.Contig18_All	novel_mir61	Up	CL11652.Contig23_All	miR166h-3p	Down	CL5531.Contig5_All
miR396a-3p_1	Down	CL5140.Contig8_All	novel_mir61	Up	CL11652.Contig28_All	miR166h-3p	Down	CL252.Contig9_All
miR396a-3p_1	Down	CL5140.Contig4_All	novel_mir61	Up	CL11514.Contig4_All	miR166h-3p	Down	CL127.Contig8_All
miR396a-3p_1	Down	CL5140.Contig1_All	novel_mir61	Up	CL6502.Contig3_All	miR166h-3p	Down	CL127.Contig9_All
miR396a-3p_1	Down	Unigene13456_All	novel_mir61	Up	CL11652.Contig32_All	miR166h-3p	Down	Unigene19733_All
miR396a-3p_1	Down	CL10745.Contig5_All	miR396a-3p_1	Up	CL5140.Contig8_All	miR166h-3p	Down	Unigene23833_All
miR396a-3p_1	Down	CL2219.Contig7_All	miR396a-3p_1	Up	CL5140.Contig4_All	miR166h-3p	Down	Unigene23835_All
miR396a-3p_1	Down	CL5140.Contig7_All	miR396a-3p_1	Up	CL5140.Contig1_All	miR166h-3p	Down	CL127.Contig13_All
miR396a-3p_1	Down	CL5140.Contig3_All	miR396a-3p_1	Up	Unigene13456_All	miR166h-3p	Down	Unigene23839_All
miR396a-3p_1	Down	CL2219.Contig4_All	miR396a-3p_1	Up	CL10745.Contig5_All	miR166h-3p	Down	CL127.Contig7_All
miR396a-3p_1	Down	CL6647.Contig4_All	miR396a-3p_1	Up	CL2219.Contig7_All	miR166h-3p	Down	Unigene19729_All
miR396a-3p_1	Down	Unigene26657_All	miR396a-3p_1	Up	CL5140.Contig7_All	miR166h-3p	Down	CL5531.Contig6_All
miR396a-3p_1	Down	CL7970.Contig1_All	miR396a-3p_1	Up	CL5140.Contig3_All	miR166h-3p	Down	CL127.Contig6_All
miR396a-3p_1	Down	CL7970.Contig2_All	miR396a-3p_1	Up	CL2219.Contig4_All	miR166h-3p	Down	Unigene23829_All
miR396a-3p_1	Down	CL10745.Contig2_All	miR396a-3p_1	Up	CL6647.Contig4_All	miR166h-3p	Down	CL127.Contig1_All
miR396a-3p_1	Down	CL2219.Contig9_All	miR396a-3p_1	Up	Unigene26657_All	miR166h-3p	Down	CL127.Contig12_All
miR396a-3p_1	Down	CL12157.Contig3_All	miR396a-3p_1	Up	CL7970.Contig1_All	miR166h-3p	Down	CL127.Contig11_All
miR396a-3p_1	Down	CL12157.Contig2_All	miR396a-3p_1	Up	CL7970.Contig2_All	miR166h-3p	Down	CL11080.Contig3_All
miR167d-5p	Down	CL10446.Contig19_All	miR396a-3p_1	Up	CL10745.Contig2_All	miR171b-3p_3	Down	CL3157.Contig1_All
miR167d-5p	Down	Unigene16296_All	miR396a-3p_1	Up	CL2219.Contig9_All	miR171b-3p_3	Down	CL3157.Contig4_All
miR167d-5p	Down	CL10446.Contig22_All	miR396a-3p_1	Up	CL12157.Contig3_All	miR167d-5p	Down	CL10446.Contig19_All
miR167d-5p	Down	CL7620.Contig1_All	miR396a-3p_1	Up	CL12157.Contig2_All	miR167d-5p	Down	Unigene16296_All
miR167d-5p	Down	CL7620.Contig2_All	novel_mir73	Up	CL823.Contig15_All	miR167d-5p	Down	CL10446.Contig22_All
miR167d-5p	Down	CL10446.Contig18_All	novel_mir73	Up	CL823.Contig3_All	miR167d-5p	Down	CL7620.Contig1_All
novel_mir22	Down	Unigene32506_All	novel_mir73	Up	CL823.Contig4_All	miR167d-5p	Down	CL7620.Contig2_All

**novel_mir22**	**Down**	**CL11936.Contig1_All**	**novel_mir11**	**Up**	**CL11597.Contig3_All**	**miR167d-5p**	**Down**	**CL10446.Contig18_All**
**novel_mir22**	**Down**	**Unigene9245_All**	**novel_mir11**	**Up**	**Unigene4441_All**	**miR6300**	**Down**	**CL2760.Contig9_All**
**novel_mir22**	**Down**	**CL1767.Contig15_All**	**novel_mir125**	**Up**	**Unigene27688_All**	**miR6300**	**Down**	**CL4050.Contig2_All**
**novel_mir22**	**Down**	**CL11936.Contig3_All**	**novel_mir125**	**Up**	**CL1025.Contig6_All**	**miR6300**	**Down**	**CL4050.Contig13_All**
**novel_mir22**	**Down**	**CL10666.Contig2_All**	**novel_mir125**	**Up**	**CL9876.Contig6_All**	**miR6300**	**Down**	**CL11509.Contig2_All**
**novel_mir22**	**Down**	**CL11690.Contig11_All**	**novel_mir179**	**Up**	**CL163.Contig3_All**	**miR6300**	**Down**	**CL2760.Contig2_All**
**novel_mir22**	**Down**	**Unigene12250_All**	**novel_mir179**	**Up**	**Unigene32511_All**	**miR6300**	**Down**	**CL11863.Contig24_All**
**novel_mir22**	**Down**	**CL11690.Contig6_All**	**novel_mir179**	**Up**	**CL7229.Contig5_All**	**miR6300**	**Down**	**CL2896.Contig7_All**
**novel_mir22**	**Down**	**CL1767.Contig19_All**	**novel_mir179**	**Up**	**Unigene3801_All**	**miR6300**	**Down**	**CL12239.Contig2_All**
**novel_mir22**	**Down**	**Unigene23188_All**	**novel_mir179**	**Up**	**CL8468.Contig3_All**	**miR6300**	**Down**	**CL3349.Contig9_All**
**novel_mir22**	**Down**	**CL11936.Contig6_All**	**novel_mir179**	**Up**	**CL163.Contig9_All**	**miR6300**	**Down**	**CL2063.Contig11_All**
**novel_mir22**	**Down**	**CL11690.Contig3_All**	**novel_mir179**	**Up**	**CL9876.Contig6_All**	**miR6300**	**Down**	**CL4808.Contig1_All**
**novel_mir22**	**Down**	**CL11936.Contig2_All**	**novel_mir179**	**Up**	**CL368.Contig9_All**	**miR6300**	**Down**	**CL11863.Contig1_All**
**novel_mir22**	**Down**	**CL11690.Contig4_All**	**novel_mir179**	**Up**	**CL8705.Contig6_All**	**miR6300**	**Down**	**CL3661.Contig15_All**
**novel_mir22**	**Down**	**CL451.Contig1_All**	**novel_mir179**	**Up**	**CL3878.Contig8_All**	**miR6300**	**Down**	**CL2760.Contig7_All**
**novel_mir22**	**Down**	**Unigene11422_All**	**novel_mir179**	**Up**	**Unigene9362_All**	**miR6300**	**Down**	**CL9543.Contig1_All**
**novel_mir22**	**Down**	**Unigene10264_All**	**novel_mir179**	**Up**	**CL1737.Contig8_All**	**miR6300**	**Down**	**CL6146.Contig4_All**
**novel_mir75**	**Down**	**CL11927.Contig2_All**	**novel_mir179**	**Up**	**CL6753.Contig12_All**	**miR6300**	**Down**	**CL3368.Contig8_All**
**novel_mir75**	**Down**	**CL9741.Contig7_All**	**novel_mir179**	**Up**	**CL6239.Contig4_All**	**miR6300**	**Down**	**CL4050.Contig18_All**
**novel_mir75**	**Down**	**CL7875.Contig1_All**	**novel_mir179**	**Up**	**CL1737.Contig9_All**	**miR6300**	**Down**	**CL1217.Contig2_All**
**novel_mir75**	**Down**	**CL9741.Contig6_All**	**novel_mir179**	**Up**	**Unigene12107_All**	**miR6300**	**Down**	**CL2760.Contig1_All**
**novel_mir75**	**Down**	**CL9741.Contig4_All**	**novel_mir179**	**Up**	**Unigene12111_All**	**miR6300**	**Down**	**CL3368.Contig5_All**
**miR397a_3**	**Down**	**Unigene31825_All**	**novel_mir179**	**Up**	**CL9876.Contig14_All**	**miR6300**	**Down**	**CL2696.Contig4_All**
**miR397a_3**	**Down**	**CL5992.Contig2_All**	**novel_mir179**	**Up**	**Unigene4733_All**	**miR6300**	**Down**	**CL3661.Contig14_All**
**miR397a_3**	**Down**	**CL11482.Contig2_All**	**novel_mir179**	**Up**	**Unigene4444_All**	**miR6300**	**Down**	**CL6146.Contig6_All**
**miR397a_3**	**Down**	**CL6305.Contig6_All**	**novel_mir179**	**Up**	**CL11824.Contig4_All**	**miR6300**	**Down**	**CL656.Contig8_All**
**miR397a_3**	**Down**	**CL5992.Contig3_All**	**novel_mir179**	**Up**	**Unigene6545_All**	**miR6300**	**Down**	**CL2696.Contig5_All**
**miR397a_3**	**Down**	**CL11482.Contig1_All**	**novel_mir179**	**Up**	**CL9876.Contig17_All**	**miR6300**	**Down**	**CL3661.Contig2_All**

**miR397a_3**	**Down**	**CL235.Contig2_All**	**novel_mir179**	**Up**	**CL59.Contig7_All**	**miR6300**	**Down**	**CL3661.Contig9_All**
**miR397a_3**	**Down**	**CL8692.Contig1_All**	**novel_mir179**	**Up**	**CL10467.Contig13_All**	**miR6300**	**Down**	**CL4050.Contig5_All**
**miR397a_3**	**Down**	**CL6305.Contig7_All**	**novel_mir179**	**Up**	**CL1212.Contig1_All**	**miR6300**	**Down**	**Unigene1749_All**
**miR397a_3**	**Down**	**CL11442.Contig1_All**	**novel_mir179**	**Up**	**Unigene8658_All**	**miR6300**	**Down**	**Unigene36691_All**
**miR397a_3**	**Down**	**CL8692.Contig2_All**	**novel_mir179**	**Up**	**Unigene16780_All**	**miR6300**	**Down**	**CL2896.Contig6_All**
**novel_mir119**	**Down**	**Unigene3605_All**	**novel_mir179**	**Up**	**CL2997.Contig3_All**	**miR6300**	**Down**	**CL4050.Contig11_All**
**novel_mir129**	**Down**	**CL5381.Contig3_All**	**novel_mir179**	**Up**	**Unigene2127_All**	**miR6300**	**Down**	**CL656.Contig40_All**
**novel_mir129**	**Down**	**Unigene30000_All**	**novel_mir179**	**Up**	**Unigene2556_All**	**miR6300**	**Down**	**Unigene434_All**
**novel_mir129**	**Down**	**Unigene9369_All**	**novel_mir179**	**Up**	**CL6668.Contig7_All**	**miR6300**	**Down**	**CL4050.Contig17_All**
**novel_mir129**	**Down**	**CL11930.Contig2_All**	**novel_mir179**	**Up**	**Unigene10699_All**	**miR6300**	**Down**	**CL1431.Contig2_All**
**novel_mir129**	**Down**	**Unigene10067_All**	**novel_mir179**	**Up**	**Unigene6405_All**	**miR6300**	**Down**	**CL4050.Contig9_All**
**novel_mir129**	**Down**	**CL11930.Contig3_All**	**novel_mir179**	**Up**	**Unigene3222_All**	**miR6300**	**Down**	**CL3661.Contig3_All**
**novel_mir129**	**Down**	**CL1290.Contig27_All**	**novel_mir179**	**Up**	**CL7229.Contig6_All**	**miR6300**	**Down**	**CL11863.Contig23_All**
**novel_mir129**	**Down**	**CL1290.Contig42_All**	**novel_mir179**	**Up**	**CL1222.Contig7_All**	**miR6300**	**Down**	**Unigene35124_All**
**novel_mir151**	**Down**	**Unigene3477_All**	**novel_mir179**	**Up**	**CL8705.Contig5_All**	**miR6300**	**Down**	**Unigene28554_All**
**novel_mir151**	**Down**	**Unigene12426_All**	**novel_mir179**	**Up**	**CL12315.Contig15_All**	**miR6300**	**Down**	**CL3368.Contig4_All**
**novel_mir151**	**Down**	**CL1290.Contig42_All**	**novel_mir179**	**Up**	**CL1212.Contig2_All**	**miR6300**	**Down**	**CL3368.Contig9_All**
**novel_mir151**	**Down**	**CL222.Contig47_All**	**novel_mir179**	**Up**	**CL5614.Contig2_All**	**miR6300**	**Down**	**CL3661.Contig10_All**
**novel_mir151**	**Down**	**CL1925.Contig3_All**	**novel_mir179**	**Up**	**CL6753.Contig18_All**	**miR6300**	**Down**	**CL2760.Contig3_All**
**novel_mir151**	**Down**	**CL11286.Contig9_All**	**novel_mir179**	**Up**	**CL12315.Contig11_All**	**miR6300**	**Down**	**CL1431.Contig3_All**
**novel_mir151**	**Down**	**CL11286.Contig28_All**	**novel_mir179**	**Up**	**Unigene4272_All**	**miR6300**	**Down**	**CL656.Contig10_All**
**novel_mir151**	**Down**	**CL6270.Contig16_All**	**novel_mir179**	**Up**	**Unigene25992_All**	**miR6300**	**Down**	**CL6146.Contig3_All**
**novel_mir151**	**Down**	**CL2947.Contig1_All**	**novel_mir179**	**Up**	**CL10467.Contig17_All**	**miR6300**	**Down**	**CL2760.Contig6_All**
**novel_mir151**	**Down**	**CL6270.Contig9_All**	**novel_mir179**	**Up**	**CL2997.Contig5_All**	**miR6300**	**Down**	**CL2063.Contig15_All**
**novel_mir151**	**Down**	**Unigene11539_All**	**novel_mir179**	**Up**	**Unigene11802_All**	**miR6300**	**Down**	**CL2896.Contig5_All**
**novel_mir151**	**Down**	**CL8022.Contig1_All**	**novel_mir179**	**Up**	**CL1277.Contig2_All**	**miR6300**	**Down**	**CL2696.Contig8_All**
**novel_mir151**	**Down**	**CL6270.Contig8_All**	**novel_mir179**	**Up**	**CL12206.Contig27_All**	**miR6300**	**Down**	**CL2896.Contig3_All**
**novel_mir151**	**Down**	**CL6270.Contig3_All**	**novel_mir179**	**Up**	**CL463.Contig6_All**	**miR6300**	**Down**	**CL4050.Contig1_All**

novel_mir151	Down	CL4511.Contig1_All	novel_mir179	Up	CL6757.Contig1_All	miR6300	Down	CL1217.Contig3_All
novel_mir151	Down	CL11930.Contig3_All	novel_mir179	Up	Unigene5058_All	miR6300	Down	CL2696.Contig10_All
novel_mir151	Down	CL12266.Contig2_All	novel_mir179	Up	CL2997.Contig2_All	miR6300	Down	CL6146.Contig5_All
novel_mir151	Down	Unigene2213_All	novel_mir179	Up	CL8668.Contig13_All	miR6300	Down	CL3349.Contig4_All
novel_mir151	Down	CL291.Contig6_All	novel_mir179	Up	CL5614.Contig4_All	miR6300	Down	CL2063.Contig1_All
novel_mir151	Down	Unigene31208_All	novel_mir179	Up	Unigene11357_All	miR6300	Down	CL11946.Contig2_All
novel_mir151	Down	CL5850.Contig4_All	novel_mir179	Up	CL12253.Contig7_All	miR6300	Down	CL2063.Contig2_All
novel_mir151	Down	CL994.Contig5_All	novel_mir179	Up	Unigene8781_All	miR6300	Down	CL4050.Contig4_All
novel_mir151	Down	CL8986.Contig16_All	novel_mir179	Up	CL10467.Contig8_All	miR6300	Down	CL656.Contig52_All
novel_mir151	Down	CL6270.Contig1_All	novel_mir179	Up	CL7229.Contig2_All	miR6300	Down	Unigene14900_All
novel_mir151	Down	CL11286.Contig5_All	novel_mir179	Up	CL1222.Contig2_All	miR6300	Down	CL656.Contig51_All
novel_mir151	Down	CL6270.Contig6_All	novel_mir179	Up	CL10467.Contig6_All	miR6300	Down	CL656.Contig7_All
novel_mir151	Down	CL11286.Contig19_All	novel_mir179	Up	CL7296.Contig4_All	miR6300	Down	CL3368.Contig6_All
novel_mir151	Down	CL11286.Contig38_All	novel_mir179	Up	Unigene7639_All	novel_mir22	Down	Unigene32506_All
novel_mir151	Down	Unigene5040_All	novel_mir179	Up	Unigene9623_All	novel_mir22	Down	CL11936.Contig1_All
novel_mir151	Down	CL163.Contig21_All	novel_mir179	Up	CL1737.Contig7_All	novel_mir22	Down	Unigene9245_All
novel_mir151	Down	CL5804.Contig3_All	novel_mir179	Up	CL1737.Contig3_All	novel_mir22	Down	CL1767.Contig15_All
novel_mir151	Down	Unigene10067_All	novel_mir179	Up	CL9876.Contig16_All	novel_mir22	Down	CL11936.Contig3_All
novel_mir151	Down	Unigene7755_All	novel_mir179	Up	CL9876.Contig18_All	novel_mir22	Down	CL10666.Contig2_All
novel_mir151	Down	Unigene9326_All	novel_mir179	Up	CL7229.Contig3_All	novel_mir22	Down	CL11690.Contig11_All
novel_mir151	Down	CL11286.Contig13_All	novel_mir179	Up	CL1212.Contig4_All	novel_mir22	Down	Unigene12250_All
novel_mir151	Down	Unigene255_All	novel_mir179	Up	Unigene3858_All	novel_mir22	Down	CL11690.Contig6_All
novel_mir151	Down	CL11286.Contig11_All	novel_mir179	Up	CL163.Contig10_All	novel_mir22	Down	CL1767.Contig19_All
novel_mir151	Down	CL994.Contig6_All	novel_mir179	Up	CL4206.Contig6_All	novel_mir22	Down	Unigene23188_All
novel_mir151	Down	CL291.Contig16_All	novel_mir179	Up	CL6753.Contig13_All	novel_mir22	Down	CL11936.Contig6_All
novel_mir151	Down	CL4040.Contig1_All	novel_mir179	Up	CL9876.Contig2_All	novel_mir22	Down	CL11690.Contig3_All
novel_mir151	Down	CL11286.Contig31_All	novel_mir179	Up	CL6668.Contig11_All	novel_mir22	Down	CL11936.Contig2_All
novel_mir151	Down	CL291.Contig18_All	novel_mir179	Up	CL1737.Contig28_All	novel_mir22	Down	CL11690.Contig4_All

novel_mir151	Down	CL222.Contig44_All	novel_mir179	Up	CL3878.Contig13_All	novel_mir22	Down	CL451.Contig1_All
novel_mir151	Down	CL4511.Contig2_All	novel_mir179	Up	CL9876.Contig13_All	novel_mir22	Down	Unigene11422_All
novel_mir151	Down	CL11286.Contig14_All	novel_mir179	Up	Unigene5756_All	novel_mir22	Down	Unigene10264_All
novel_mir151	Down	CL12266.Contig1_All	novel_mir179	Up	CL12323.Contig2_All	miR166u	Down	Unigene23840_All
novel_mir151	Down	CL1290.Contig17_All	novel_mir179	Up	Unigene2668_All	miR166u	Down	CL252.Contig8_All
novel_mir151	Down	CL5937.Contig59_All	novel_mir179	Up	Unigene18222_All	miR166u	Down	CL252.Contig7_All
novel_mir151	Down	CL11286.Contig39_All	novel_mir179	Up	CL8668.Contig16_All	miR166u	Down	CL252.Contig9_All
novel_mir151	Down	CL291.Contig4_All	novel_mir179	Up	Unigene5068_All	miR166u	Down	CL127.Contig8_All
novel_mir151	Down	CL11930.Contig2_All	novel_mir179	Up	Unigene7634_All	miR166u	Down	CL127.Contig9_All
novel_mir151	Down	CL6270.Contig4_All	novel_mir179	Up	CL1222.Contig4_All	miR166u	Down	Unigene19733_All
novel_mir151	Down	CL11286.Contig4_All	novel_mir179	Up	CL1737.Contig14_All	miR166u	Down	Unigene23833_All
novel_mir151	Down	Unigene12079_All	novel_mir179	Up	CL163.Contig4_All	miR166u	Down	Unigene23835_All
novel_mir151	Down	CL222.Contig28_All	novel_mir179	Up	CL12315.Contig6_All	miR166u	Down	CL4375.Contig3_All
novel_mir151	Down	CL6270.Contig13_All	novel_mir179	Up	CL8668.Contig14_All	miR166u	Down	CL127.Contig13_All
novel_mir151	Down	CL1925.Contig2_All	novel_mir179	Up	CL8468.Contig1_All	miR166u	Down	Unigene23839_All
novel_mir151	Down	CL5381.Contig3_All	novel_mir179	Up	Unigene2465_All	miR166u	Down	CL4375.Contig1_All
novel_mir151	Down	CL6270.Contig5_All	novel_mir179	Up	CL11123.Contig1_All	miR166u	Down	CL127.Contig7_All
novel_mir151	Down	CL4170.Contig8_All	novel_mir179	Up	CL9876.Contig3_All	miR166u	Down	Unigene19729_All
novel_mir151	Down	Unigene23096_All	novel_mir179	Up	CL11594.Contig5_All	miR166u	Down	CL127.Contig6_All
novel_mir151	Down	CL291.Contig12_All	novel_mir179	Up	CL9876.Contig8_All	miR166u	Down	Unigene23829_All
novel_mir151	Down	Unigene6866_All	novel_mir179	Up	CL163.Contig20_All	miR166u	Down	CL127.Contig1_All
novel_mir151	Down	Unigene6520_All	novel_mir179	Up	CL6753.Contig2_All	miR166u	Down	CL127.Contig12_All
			novel_mir179	Up	CL1737.Contig32_All	miR166u	Down	CL127.Contig11_All
			novel_mir179	Up	Unigene9756_All	miR166u	Down	CL11080.Contig3_All
			novel_mir179	Up	CL2997.Contig1_All	novel_mir148	Down	Unigene5871_All
			novel_mir179	Up	Unigene6459_All	novel_mir148	Down	Unigene8713_All
			novel_mir179	Up	CL3878.Contig4_All	novel_mir48	Down	CL3535.Contig4_All
			novel_mir179	Up	CL8705.Contig2_All	novel_mir48	Down	CL10714.Contig2_All

			novel_mir179	Up	CL4955.Contig15_All	novel_mir151	Down	Unigene3477_All
			novel_mir179	Up	CL40.Contig26_All	novel_mir151	Down	Unigene12426_All
			novel_mir179	Up	CL12315.Contig1_All	novel_mir151	Down	CL1290.Contig42_All
			novel_mir179	Up	CL7996.Contig2_All	novel_mir151	Down	CL222.Contig47_All
			novel_mir179	Up	CL8468.Contig2_All	novel_mir151	Down	CL1925.Contig3_All
			novel_mir179	Up	CL2520.Contig1_All	novel_mir151	Down	CL11286.Contig9_All
			novel_mir179	Up	Unigene10799_All	novel_mir151	Down	CL11286.Contig28_All
			novel_mir179	Up	CL7996.Contig1_All	novel_mir151	Down	CL6270.Contig16_All
			novel_mir179	Up	Unigene4275_All	novel_mir151	Down	CL2947.Contig1_All
			novel_mir179	Up	CL11824.Contig6_All	novel_mir151	Down	CL6270.Contig9_All
			novel_mir179	Up	CL1222.Contig3_All	novel_mir151	Down	Unigene11539_All
			novel_mir179	Up	CL12116.Contig8_All	novel_mir151	Down	CL8022.Contig1_All
			novel_mir179	Up	CL4206.Contig8_All	novel_mir151	Down	CL6270.Contig8_All
			novel_mir179	Up	CL368.Contig8_All	novel_mir151	Down	CL6270.Contig3_All
			novel_mir179	Up	CL11400.Contig1_All	novel_mir151	Down	CL4511.Contig1_All
			novel_mir179	Up	CL163.Contig1_All	novel_mir151	Down	CL11930.Contig3_All
			novel_mir179	Up	Unigene9281_All	novel_mir151	Down	CL12266.Contig2_All
			novel_mir179	Up	CL11400.Contig7_All	novel_mir151	Down	Unigene2213_All
			miR160a-5p	Down	CL8053.Contig2_All	novel_mir151	Down	CL291.Contig6_All
			miR160a-5p	Down	CL11742.Contig1_All	novel_mir151	Down	Unigene31208_All
			miR160a-5p	Down	CL11742.Contig4_All	novel_mir151	Down	CL5850.Contig4_All
			miR160a-5p	Down	CL11742.Contig3_All	novel_mir151	Down	CL994.Contig5_All
			miR160a-5p	Down	CL8053.Contig3_All	novel_mir151	Down	CL8986.Contig16_All
			miR160a-5p	Down	CL8053.Contig1_All	novel_mir151	Down	CL6270.Contig1_All
			miR160a-5p	Down	CL11742.Contig2_All	novel_mir151	Down	CL11286.Contig5_All
			miR160a-5p	Down	CL7275.Contig1_All	novel_mir151	Down	CL6270.Contig6_All
			miR160a-5p	Down	CL7275.Contig2_All	novel_mir151	Down	CL11286.Contig19_All
			miR166h-3p	Down	Unigene23840_All	novel_mir151	Down	CL11286.Contig38_All

			miR166h-3p	Down	CL5531.Contig2_All	novel_mir151	Down	Unigene5040_All
			miR166h-3p	Down	CL252.Contig8_All	novel_mir151	Down	CL163.Contig21_All
			miR166h-3p	Down	CL252.Contig7_All	novel_mir151	Down	CL5804.Contig3_All
			miR166h-3p	Down	CL5531.Contig5_All	novel_mir151	Down	Unigene10067_All
			miR166h-3p	Down	CL252.Contig9_All	novel_mir151	Down	Unigene7755_All
			miR166h-3p	Down	CL127.Contig8_All	novel_mir151	Down	Unigene9326_All
			miR166h-3p	Down	CL127.Contig9_All	novel_mir151	Down	CL11286.Contig13_All
			miR166h-3p	Down	Unigene19733_All	novel_mir151	Down	Unigene255_All
			miR166h-3p	Down	Unigene23833_All	novel_mir151	Down	CL11286.Contig11_All
			miR166h-3p	Down	Unigene23835_All	novel_mir151	Down	CL994.Contig6_All
			miR166h-3p	Down	CL127.Contig13_All	novel_mir151	Down	CL291.Contig16_All
			miR166h-3p	Down	Unigene23839_All	novel_mir151	Down	CL4040.Contig1_All
			miR166h-3p	Down	CL127.Contig7_All	novel_mir151	Down	CL11286.Contig31_All
			miR166h-3p	Down	Unigene19729_All	novel_mir151	Down	CL291.Contig18_All
			miR166h-3p	Down	CL5531.Contig6_All	novel_mir151	Down	CL222.Contig44_All
			miR166h-3p	Down	CL127.Contig6_All	novel_mir151	Down	CL4511.Contig2_All
			miR166h-3p	Down	Unigene23829_All	novel_mir151	Down	CL11286.Contig14_All
			miR166h-3p	Down	CL127.Contig1_All	novel_mir151	Down	CL12266.Contig1_All
			miR166h-3p	Down	CL127.Contig12_All	novel_mir151	Down	CL1290.Contig17_All
			miR166h-3p	Down	CL127.Contig11_All	novel_mir151	Down	CL5937.Contig59_All
			miR166h-3p	Down	CL11080.Contig3_All	novel_mir151	Down	CL11286.Contig39_All
			novel_mir75	Down	CL11927.Contig2_All	novel_mir151	Down	CL291.Contig4_All
			novel_mir75	Down	CL9741.Contig7_All	novel_mir151	Down	CL11930.Contig2_All
			novel_mir75	Down	CL7875.Contig1_All	novel_mir151	Down	CL6270.Contig4_All
			novel_mir75	Down	CL9741.Contig6_All	novel_mir151	Down	CL11286.Contig4_All
			novel_mir75	Down	CL9741.Contig4_All	novel_mir151	Down	Unigene12079_All
			miR171b-3p_3	Down	CL3157.Contig1_All	novel_mir151	Down	CL222.Contig28_All
			miR171b-3p_3	Down	CL3157.Contig4_All	novel_mir151	Down	CL6270.Contig13_All

			novel_mir160	Down	Unigene7853_All	novel_mir151	Down	CL1925.Contig2_All
			novel_mir160	Down	CL9723.Contig29_All	novel_mir151	Down	CL5381.Contig3_All
			novel_mir160	Down	Unigene7354_All	novel_mir151	Down	CL6270.Contig5_All
			novel_mir160	Down	Unigene10569_All	novel_mir151	Down	CL4170.Contig8_All
			novel_mir160	Down	CL10436.Contig2_All	novel_mir151	Down	Unigene23096_All
			novel_mir160	Down	Unigene35573_All	novel_mir151	Down	CL291.Contig12_All
			novel_mir160	Down	CL9115.Contig1_All	novel_mir151	Down	Unigene6866_All
			novel_mir166	Down	Unigene7727_All	novel_mir151	Down	Unigene6520_All
			novel_mir166	Down	Unigene11364_All	novel_mir172	Down	Unigene9713_All
			novel_mir79	Down	Unigene19726_All	novel_mir172	Down	Unigene8358_All
			novel_mir79	Down	CL1290.Contig17_All	novel_mir172	Down	CL947.Contig1_All
			novel_mir79	Down	Unigene33738_All	novel_mir138	Down	CL1673.Contig9_All
			novel_mir79	Down	Unigene9369_All	novel_mir138	Down	CL1673.Contig5_All
			novel_mir79	Down	CL1682.Contig15_All	novel_mir138	Down	CL1673.Contig7_All
			novel_mir79	Down	CL12266.Contig2_All	novel_mir138	Down	CL1673.Contig4_All
			novel_mir79	Down	Unigene12426_All	novel_mir138	Down	CL1673.Contig15_All
			novel_mir79	Down	CL1290.Contig42_All	novel_mir138	Down	CL1673.Contig16_All
			novel_mir79	Down	Unigene25401_All	novel_mir138	Down	CL1673.Contig8_All
			novel_mir79	Down	Unigene12079_All			
			novel_mir79	Down	Unigene7755_All			
			novel_mir79	Down	Unigene9326_All			
			novel_mir79	Down	CL1925.Contig2_All			
			novel_mir79	Down	CL1748.Contig11_All			
			novel_mir79	Down	Unigene10099_All			
			novel_mir79	Down	CL994.Contig6_All			
			novel_mir79	Down	Unigene3436_All			
			novel_mir79	Down	CL1290.Contig27_All			
			novel_mir79	Down	Unigene6520_All			

			novel_mir79	Down	CL8022.Contig1_All			
			novel_mir79	Down	Unigene35106_All			
			novel_mir161	Down	Unigene5846_All			
			novel_mir161	Down	CL5937.Contig42_All			
			novel_mir161	Down	CL5937.Contig55_All			
			novel_mir161	Down	CL5290.Contig2_All			
			novel_mir161	Down	CL6981.Contig1_All			
			novel_mir161	Down	CL3265.Contig7_All			
			novel_mir161	Down	Unigene13971_All			
			novel_mir161	Down	CL5937.Contig57_All			
			novel_mir161	Down	Unigene5995_All			
			novel_mir161	Down	Unigene9188_All			
			novel_mir161	Down	Unigene9982_All			
			novel_mir161	Down	CL12079.Contig4_All			
			novel_mir161	Down	Unigene11533_All			
			novel_mir161	Down	CL12282.Contig3_All			
			novel_mir161	Down	Unigene5935_All			
			novel_mir161	Down	CL5937.Contig76_All			
			novel_mir161	Down	CL10762.Contig2_All			
			novel_mir161	Down	CL5937.Contig2_All			
			novel_mir47	Down	Unigene11437_All			
			novel_mir47	Down	Unigene2403_All			
			novel_mir47	Down	Unigene4672_All			
			novel_mir47	Down	Unigene9755_All			
			novel_mir47	Down	CL1485.Contig4_All			
			novel_mir47	Down	Unigene7432_All			
			novel_mir47	Down	CL11930.Contig5_All			
			novel_mir47	Down	Unigene11850_All			

			novel_mir47	Down	CL11930.Contig11_All			
			miR167d_1	Down	CL1264.Contig9_All			
			miR167d_1	Down	CL10446.Contig1_All			
			miR167d_1	Down	Unigene9900_All			
			miR167d_1	Down	CL1264.Contig7_All			
			miR167d_1	Down	CL1264.Contig1_All			
			miR167d_1	Down	CL10446.Contig22_All			
			miR167d_1	Down	CL1085.Contig12_All			
			miR167d_1	Down	CL7983.Contig2_All			
			miR167d_1	Down	CL1264.Contig10_All			
			miR167d_1	Down	CL1264.Contig3_All			
			miR167d_1	Down	CL5786.Contig2_All			
			miR167d_1	Down	CL1264.Contig8_All			
			miR167d_1	Down	CL1264.Contig11_All			
			miR167d_1	Down	Unigene16296_All			
			miR167d_1	Down	CL5786.Contig8_All			
			miR167d_1	Down	Unigene2643_All			
			miR167d_1	Down	CL1264.Contig4_All			
			miR167d_1	Down	Unigene8353_All			
			miR167d_1	Down	CL3833.Contig1_All			
			miR167d_1	Down	CL5786.Contig1_All			
			miR167d_1	Down	CL5786.Contig7_All			
			miR167d_1	Down	Unigene12094_All			
			miR167d_1	Down	CL5786.Contig3_All			
			miR167d_1	Down	CL1085.Contig8_All			
			miR167d_1	Down	CL10446.Contig9_All			
			miR167d_1	Down	CL10446.Contig19_All			
			miR167d_1	Down	CL10446.Contig13_All			

			miR167d_1	Down	CL1264.Contig5_All			
			miR167d_1	Down	CL1264.Contig2_All			
			miR167d_1	Down	CL10446.Contig4_All			
			miR167d_1	Down	CL1264.Contig12_All			
			miR167d_1	Down	CL10446.Contig18_All			
			miR167d-5p	Down	CL10446.Contig19_All			
			miR167d-5p	Down	Unigene16296_All			
			miR167d-5p	Down	CL10446.Contig22_All			
			miR167d-5p	Down	CL7620.Contig1_All			
			miR167d-5p	Down	CL7620.Contig2_All			
			miR167d-5p	Down	CL10446.Contig18_All			
			miR396b	Down	CL2423.Contig5_All			
			miR396b	Down	CL623.Contig1_All			
			miR396b	Down	CL2423.Contig3_All			
			miR396b	Down	Unigene13133_All			
			miR396b	Down	CL7762.Contig2_All			
			miR396b	Down	CL82.Contig3_All			
			miR396b	Down	CL3856.Contig7_All			
			miR396b	Down	CL2267.Contig9_All			
			miR396b	Down	CL2087.Contig1_All			
			miR396b	Down	CL3856.Contig8_All			
			miR396b	Down	CL4429.Contig2_All			
			miR396b	Down	CL1599.Contig7_All			
			miR396b	Down	CL7970.Contig2_All			
			miR396b	Down	CL7970.Contig1_All			
			miR396b	Down	Unigene13134_All			
			miR396b	Down	CL623.Contig2_All			
			miR396b	Down	Unigene8745_All			

			miR396b	Down	CL5385.Contig3_All			
			miR396b	Down	Unigene26211_All			
			miR396b	Down	CL623.Contig3_All			
			miR396b	Down	CL5298.Contig2_All			
			miR396b	Down	Unigene15827_All			
			miR396b	Down	CL2423.Contig8_All			
			miR396b	Down	Unigene13456_All			
			miR396b	Down	CL82.Contig2_All			
			miR396b	Down	CL2423.Contig6_All			
			miR396b	Down	CL2423.Contig7_All			
			miR396b	Down	CL2267.Contig1_All			
			miR396b	Down	CL7970.Contig3_All			
			miR396b	Down	CL3856.Contig4_All			
			miR396b	Down	Unigene20205_All			
			miR396b	Down	CL10458.Contig4_All			
			miR396b	Down	CL1599.Contig4_All			
			miR396b	Down	CL2423.Contig4_All			
			miR396b	Down	CL82.Contig1_All			
			novel_mir56	Down	CL5937.Contig55_All			
			novel_mir56	Down	CL12282.Contig3_All			
			novel_mir56	Down	CL5937.Contig76_All			
			novel_mir56	Down	CL7137.Contig6_All			
			novel_mir56	Down	Unigene25894_All			
			novel_mir56	Down	Unigene32827_All			
			novel_mir56	Down	CL2960.Contig3_All			
			novel_mir138	Down	CL1673.Contig9_All			
			novel_mir138	Down	CL1673.Contig5_All			
			novel_mir138	Down	CL1673.Contig7_All			

			novel_mir138	Down	CL1673.Contig4_All			
			novel_mir138	Down	CL1673.Contig15_All			
			novel_mir138	Down	CL1673.Contig16_All			
			novel_mir138	Down	CL1673.Contig8_All			
			novel_mir13	Down	Unigene4506_All			
			novel_mir13	Down	CL11652.Contig31_All			
			novel_mir13	Down	Unigene6986_All			
			novel_mir13	Down	Unigene4130_All			
			novel_mir13	Down	Unigene11664_All			
			novel_mir48	Down	CL3535.Contig4_All			
			novel_mir48	Down	CL10714.Contig2_All			
			miR396h	Down	CL2423.Contig5_All			
			miR396h	Down	CL623.Contig3_All			
			miR396h	Down	CL623.Contig1_All			
			miR396h	Down	CL5884.Contig4_All			
			miR396h	Down	CL5298.Contig2_All			
			miR396h	Down	CL2423.Contig3_All			
			miR396h	Down	CL845.Contig5_All			
			miR396h	Down	CL7762.Contig2_All			
			miR396h	Down	CL82.Contig3_All			
			miR396h	Down	Unigene15827_All			
			miR396h	Down	CL2423.Contig8_All			
			miR396h	Down	CL845.Contig8_All			
			miR396h	Down	Unigene13456_All			
			miR396h	Down	CL2267.Contig9_All			
			miR396h	Down	CL2423.Contig6_All			
			miR396h	Down	Unigene15795_All			
			miR396h	Down	CL2267.Contig1_All			

			miR396h	Down	CL845.Contig6_All			
			miR396h	Down	CL7970.Contig3_All			
			miR396h	Down	Unigene20205_All			
			miR396h	Down	CL10458.Contig4_All			
			miR396h	Down	CL845.Contig2_All			
			miR396h	Down	CL7970.Contig2_All			
			miR396h	Down	CL2423.Contig4_All			
			miR396h	Down	CL7970.Contig1_All			
			miR396h	Down	CL8399.Contig3_All			
			miR396h	Down	CL623.Contig2_All			
			miR396h	Down	Unigene8745_All			
			miR396h	Down	CL845.Contig7_All			
			miR396h	Down	CL82.Contig1_All			
			novel_mir76	Down	CL9741.Contig7_All			
			novel_mir76	Down	CL9741.Contig4_All			
			novel_mir82	Down	CL601.Contig6_All			
			novel_mir82	Down	CL11469.Contig2_All			
			novel_mir82	Down	Unigene5060_All			
			novel_mir82	Down	Unigene14756_All			
			novel_mir82	Down	CL601.Contig4_All			
			novel_mir82	Down	Unigene5920_All			
			novel_mir82	Down	CL9491.Contig1_All			
			novel_mir82	Down	CL9234.Contig4_All			
			novel_mir82	Down	Unigene3546_All			
			novel_mir82	Down	CL7212.Contig3_All			
			novel_mir82	Down	Unigene11428_All			
			novel_mir82	Down	CL11469.Contig1_All			
			novel_mir82	Down	Unigene10145_All			

			novel_mir82	Down	Unigene10278_All			
			novel_mir82	Down	Unigene2414_All			
			novel_mir82	Down	CL9811.Contig2_All			
			novel_mir82	Down	CL11143.Contig7_All			
			novel_mir45	Down	CL3161.Contig4_All			
			novel_mir45	Down	Unigene12033_All			
			novel_mir45	Down	CL5618.Contig2_All			
			novel_mir45	Down	CL5618.Contig1_All			
			novel_mir148	Down	Unigene5871_All			
			novel_mir148	Down	Unigene8713_All			
			miR166h-3p_1	Down	Unigene23840_All			
			miR166h-3p_1	Down	CL252.Contig8_All			
			miR166h-3p_1	Down	CL252.Contig7_All			
			miR166h-3p_1	Down	CL252.Contig9_All			
			miR166h-3p_1	Down	CL127.Contig8_All			
			miR166h-3p_1	Down	CL127.Contig9_All			
			miR166h-3p_1	Down	Unigene19733_All			
			miR166h-3p_1	Down	Unigene23833_All			
			miR166h-3p_1	Down	Unigene23835_All			
			miR166h-3p_1	Down	CL127.Contig13_All			
			miR166h-3p_1	Down	Unigene23839_All			
			miR166h-3p_1	Down	CL127.Contig7_All			
			miR166h-3p_1	Down	Unigene19729_All			
			miR166h-3p_1	Down	CL127.Contig6_All			
			miR166h-3p_1	Down	CL127.Contig1_All			
			miR166h-3p_1	Down	Unigene23829_All			
			miR166h-3p_1	Down	CL127.Contig12_All			
			miR166h-3p_1	Down	CL127.Contig11_All			

			miR166h-3p_1	Down	CL11080.Contig3_All			
			novel_mir119	Down	Unigene3605_All			
			miR171d_1	Down	CL9585.Contig3_All			
			miR171d_1	Down	CL3157.Contig1_All			
			miR171d_1	Down	Unigene28645_All			
			miR171d_1	Down	CL3157.Contig4_All			
			miR171d_1	Down	CL9585.Contig1_All			
			novel_mir42	Down	CL2960.Contig3_All			
			miR398b	Down	CL11427.Contig26_All			
			miR398b	Down	CL11427.Contig12_All			
			miR398b	Down	Unigene14301_All			
			miR398b	Down	CL11427.Contig24_All			
			miR398b	Down	Unigene3694_All			
			miR398b	Down	Unigene18261_All			
			miR398b	Down	Unigene7736_All			
			miR398b	Down	CL4864.Contig1_All			
			miR398b	Down	CL11427.Contig22_All			
			miR398b	Down	Unigene18921_All			
			miR398b	Down	Unigene13750_All			
			miR398b	Down	CL11427.Contig6_All			
			miR398b	Down	CL11427.Contig10_All			
			miR398b	Down	CL11427.Contig11_All			
			miR398b	Down	CL11427.Contig19_All			
			miR398b	Down	Unigene11550_All			
			novel_mir81	Down	CL11351.Contig2_All			
			miR171f_3	Down	CL3157.Contig1_All			
			miR171f_3	Down	CL3157.Contig4_All			
			novel_mir63	Down	Unigene5834_All			

			novel_mir63	Down	Unigene5561_All				
			novel_mir63	Down	Unigene9662_All				
			novel_mir63	Down	Unigene31590_All				
			novel_mir63	Down	CL968.Contig1_All				
			novel_mir63	Down	CL3247.Contig4_All				
			novel_mir63	Down	CL7082.Contig1_All				
			novel_mir63	Down	CL5952.Contig6_All				
			novel_mir63	Down	Unigene27384_All				
			novel_mir63	Down	CL2369.Contig5_All				
			novel_mir63	Down	CL5791.Contig1_All				
			novel_mir63	Down	CL5791.Contig2_All				
			novel_mir63	Down	Unigene749_All				
			novel_mir63	Down	CL7082.Contig3_All				
			novel_mir63	Down	CL6547.Contig5_All				
			novel_mir63	Down	Unigene5714_All				
			novel_mir63	Down	CL9383.Contig2_All				
			novel_mir63	Down	CL9365.Contig2_All				
			novel_mir63	Down	Unigene32862_All				
			novel_mir63	Down	Unigene12036_All				
			novel_mir63	Down	CL10158.Contig1_All				
			novel_mir63	Down	Unigene3127_All				
			novel_mir63	Down	Unigene17114_All				
			novel_mir63	Down	Unigene27383_All				
			novel_mir63	Down	CL5952.Contig5_All				
			novel_mir63	Down	Unigene31338_All				
			novel_mir63	Down	Unigene21086_All				
			novel_mir63	Down	Unigene3301_All				
			novel_mir63	Down	CL5592.Contig4_All				

			novel_mir63	Down	CL5952.Contig2_All			
			novel_mir63	Down	CL3247.Contig2_All			
			novel_mir63	Down	CL12229.Contig2_All			
			novel_mir63	Down	CL5952.Contig14_All			
			novel_mir63	Down	CL12229.Contig1_All			
			novel_mir63	Down	CL12277.Contig5_All			
			novel_mir63	Down	CL5952.Contig7_All			
			novel_mir63	Down	Unigene22412_All			
			novel_mir63	Down	CL2211.Contig3_All			
			novel_mir175	Down	CL10766.Contig1_All			
			novel_mir175	Down	CL7703.Contig2_All			
			novel_mir175	Down	Unigene8883_All			
			novel_mir175	Down	Unigene5705_All			
			novel_mir175	Down	CL10766.Contig2_All			
			novel_mir175	Down	CL3501.Contig3_All			
			novel_mir175	Down	CL10766.Contig4_All			
			novel_mir175	Down	CL10766.Contig3_All			
			novel_mir29	Down	CL12229.Contig3_All			
			novel_mir29	Down	CL11787.Contig1_All			
			novel_mir29	Down	Unigene8173_All			
			novel_mir29	Down	Unigene11403_All			
			novel_mir29	Down	Unigene12089_All			
			novel_mir29	Down	Unigene31338_All			
			novel_mir29	Down	Unigene5795_All			
			novel_mir29	Down	CL968.Contig2_All			
			novel_mir29	Down	Unigene8536_All			
			miR166a-3p	Down	Unigene23840_All			
			miR166a-3p	Down	CL252.Contig8_All			

			miR166a-3p	Down	CL252.Contig7_All			
			miR166a-3p	Down	CL252.Contig9_All			
			miR166a-3p	Down	CL127.Contig8_All			
			miR166a-3p	Down	CL127.Contig9_All			
			miR166a-3p	Down	Unigene19733_All			
			miR166a-3p	Down	Unigene23833_All			
			miR166a-3p	Down	Unigene23835_All			
			miR166a-3p	Down	CL127.Contig13_All			
			miR166a-3p	Down	Unigene23839_All			
			miR166a-3p	Down	CL127.Contig7_All			
			miR166a-3p	Down	Unigene19729_All			
			miR166a-3p	Down	CL127.Contig6_All			
			miR166a-3p	Down	CL127.Contig1_All			
			miR166a-3p	Down	Unigene23829_All			
			miR166a-3p	Down	CL127.Contig12_All			
			miR166a-3p	Down	CL127.Contig11_All			
			miR166a-3p	Down	CL11080.Contig3_All			
			novel_mir4	Down	Unigene2940_All			
			novel_mir4	Down	CL11928.Contig6_All			
			novel_mir4	Down	CL74.Contig15_All			
			novel_mir4	Down	Unigene4884_All			
			novel_mir4	Down	CL5937.Contig44_All			
			novel_mir4	Down	Unigene28660_All			
			novel_mir4	Down	CL11928.Contig1_All			
			novel_mir4	Down	CL581.Contig9_All			
			novel_mir4	Down	Unigene3898_All			
			novel_mir4	Down	Unigene2549_All			
			novel_mir4	Down	CL4833.Contig3_All			

			novel_mir4	Down	CL3783.Contig5_All			
			novel_mir4	Down	Unigene36625_All			
			novel_mir4	Down	CL1489.Contig15_All			
			novel_mir4	Down	CL329.Contig23_All			
			novel_mir4	Down	CL1390.Contig7_All			
			novel_mir4	Down	Unigene8309_All			
			novel_mir4	Down	CL581.Contig2_All			
			novel_mir4	Down	CL7613.Contig9_All			
			novel_mir4	Down	CL11928.Contig4_All			
			novel_mir4	Down	CL9183.Contig4_All			
			novel_mir4	Down	CL11823.Contig1_All			
			novel_mir4	Down	CL7613.Contig2_All			
			novel_mir4	Down	CL6538.Contig1_All			
			novel_mir4	Down	CL12137.Contig8_All			
			novel_mir4	Down	CL12137.Contig9_All			
			novel_mir4	Down	Unigene9264_All			
			novel_mir4	Down	CL1489.Contig18_All			
			novel_mir4	Down	CL74.Contig27_All			
			novel_mir4	Down	CL11928.Contig7_All			
			novel_mir4	Down	CL11928.Contig13_All			
			novel_mir4	Down	CL4833.Contig8_All			
			novel_mir4	Down	CL6294.Contig8_All			
			novel_mir4	Down	CL8870.Contig2_All			
			novel_mir4	Down	Unigene3862_All			
			novel_mir4	Down	CL4833.Contig7_All			
			novel_mir4	Down	CL5100.Contig3_All			
			novel_mir4	Down	CL11928.Contig5_All			
			novel_mir4	Down	Unigene3082_All			

			novel_mir4	Down	CL11928.Contig16_All			
			novel_mir4	Down	CL3778.Contig3_All			
			novel_mir4	Down	CL581.Contig8_All			
			novel_mir4	Down	CL11928.Contig9_All			
			novel_mir4	Down	CL6964.Contig11_All			
			novel_mir4	Down	CL4113.Contig1_All			
			novel_mir4	Down	CL8870.Contig1_All			
			novel_mir4	Down	CL6964.Contig10_All			
			novel_mir4	Down	CL329.Contig22_All			
			novel_mir4	Down	CL3783.Contig4_All			
			novel_mir4	Down	Unigene4724_All			
			novel_mir4	Down	CL11928.Contig8_All			
			novel_mir4	Down	CL1005.Contig4_All			
			novel_mir4	Down	CL1489.Contig12_All			
			novel_mir4	Down	CL9327.Contig1_All			
			novel_mir4	Down	CL4833.Contig9_All			
			novel_mir4	Down	CL11928.Contig11_All			
			novel_mir4	Down	CL74.Contig21_All			
			novel_mir4	Down	Unigene9005_All			
			novel_mir4	Down	Unigene12285_All			
			novel_mir4	Down	CL10598.Contig2_All			
			novel_mir38	Down	CL2104.Contig4_All			
			novel_mir38	Down	CL2104.Contig1_All			
			novel_mir38	Down	CL2104.Contig3_All			
			novel_mir95	Down	Unigene5919_All			
			novel_mir95	Down	Unigene11437_All			
			novel_mir95	Down	Unigene4241_All			

Table S20. Metabolic pathways of differentially expressed miRNAs in the CK vsM10

miRNA id	Target id	log2(M10/CK)	Pathway
miR396a-3p_4	CL5140.Contig4_All	19.118	Spliceosome
miR396a-3p_4	CL2219.Contig4_All	19.118	Plant-pathogen interaction
miR396a-3p_4	Unigene26657_All	19.118	Plant-pathogen interaction
miR396a-3p_4	CL10745.Contig2_All	19.118	Glutathione metabolism
novel_mir97	Unigene11539_All	9.907	Glycerophospholipid metabolism， Ether lipid metabolism， Inositol phosphate metabolism;
novel_mir97	CL11756.Contig1_All	9.907	Purine metabolism
novel_mir97	CL11930.Contig3_All	9.907	Other types of O-glycan biosynthesis， Biosynthesis of secondary metabolites，Glycerophospholipid metabolism，Ether lipid metabolism，Inositol phosphate metabolism
novel_mir106	Unigene5040_All	9.344	pliceosome
novel_mir106	Unigene3477_All	9.344	Spliceosome
novel_mir106	CL12266.Contig2_All	9.344	Spliceosome
novel_mir106	Unigene6451_All	9.344	Porphyrin and chlorophyll metabolism， Biosynthesis of secondary metabolites
novel_mir106	Unigene12079_All	9.344	Terpenoid backbone biosynthesis
novel_mir106	Unigene7755_All	9.344	Terpenoid backbone biosynthesis
novel_mir106	CL994.Contig5_All	9.344	Spliceosome
miR396a-5p	CL2267.Contig1_All	6.417	Homologous recombination

miR396a-5p	CL10458.Contig4_All	6.417	Lysine degradation， Metabolic pathways， Biosynthesis of secondary metabolites
miR396a-5p	CL623.Contig2_All	6.417	Endocytosis
novel_mir120	CL5783.Contig2_All	2.935	mRNA surveillance pathway，RNA transport
novel_mir47	Unigene2403_All	1.972	N-Glycan biosynthesis
novel_mir47	CL11930.Contig5_All	1.972	Fatty acid elongation，Fatty acid metabolism
novel_mir72	CL7956.Contig1_All	1.972	Phenylpropanoid biosynthesis
novel_mir72	CL11367.Contig4_All	1.972	Amino sugar and nucleotide sugar metabolism
novel_mir72	Unigene19898_All	1.972	MAPK signaling pathway – plant，Plant-pathogen interaction
novel_mir72	Unigene6418_All	1.972	Plant hormone signal transduction
novel_mir72	CL4372.Contig2_All	1.972	Starch and sucrose metabolism
novel_mir72	Unigene7472_All	1.972	Plant hormone signal transduction
novel_mir72	CL2494.Contig1_All	1.972	Other glycan degradation
novel_mir72	Unigene3832_All	1.972	Plant hormone signal transduction
novel_mir72	CL2352.Contig11_All	1.972	ABC transporters
novel_mir72	Unigene11537_All	1.972	RNA transport，Ribosome biogenesis in eukaryotes
novel_mir72	CL2144.Contig6_All	1.972	Alanine, aspartate and glutamate metabolism， Valine, leucine and isoleucine degradation， Glycine, serine and threonine metabolism， Cysteine and methionine metabolism
novel_mir72	Unigene10328_All	1.972	Aminoacyl-tRNA biosynthesis
novel_mir72	Unigene4211_All	1.972	Plant hormone signal transduction
novel_mir72	Unigene9743_All	1.972	Plant hormone signal transduction
novel_mir140	Unigene10722_All	1.388	Ribosome biogenesis in eukaryotes
novel_mir90	Unigene11054_All	1.313	Linoleic acid metabolism，Homologous recombination
miR156a-5p	CL11653.Contig7_All	1.199	Purine metabolism，RNA polymerase， Pyrimidine metabolism
miR156a-5p	CL4553.Contig1_All	1.199	Plant hormone signal transduction
miR156a-5p	CL2170.Contig2_All	1.199	Protein processing in endoplasmic reticulum

miR156a-5p	CL9049.Contig3_All	1.199	Plant hormone signal transduction
miR156a-5p	CL2558.Contig12_All	1.199	Purine metabolism，RNA polymerase，Pyrimidine metabolism
miR156a-5p	Unigene185_All	1.199	Purine metabolism，RNA polymerase，Pyrimidine metabolism
miR168	Unigene13184_All	1.187	Protein processing in endoplasmic reticulum，Protein export;
miR156a	CL9049.Contig3_All	1.076	Plant hormone signal transduction
miR156a	CL2170.Contig2_All	1.076	Protein processing in endoplasmic reticulum
miR156a	CL11653.Contig7_All	1.076	Purine metabolism，RNA polymerase，Pyrimidine metabolism;
miR156a	CL12256.Contig7_All	1.076	Brassinosteroid biosynthesis，Biosynthesis of secondary metabolites
miR156a	Unigene7368_All	1.076	Brassinosteroid biosynthesis， Biosynthesis of secondary metabolites
novel_mir36	Unigene5040_All	-10.937	Spliceosome
novel_mir36	CL994.Contig6_All	-10.937	Spliceosome;
novel_mir36	Unigene3477_All	-10.937	Spliceosome
novel_mir36	CL12266.Contig2_All	-10.937	Spliceosome
novel_mir36	Unigene11066_All	-10.937	Homologous recombination
novel_mir161	CL3265.Contig7_All	-9.861	Cysteine and methionine metabolism，Glutathione metabolism
novel_mir161	Unigene9188_All	-9.861	RNA degradation
novel_mir161	CL10762.Contig2_All	-9.861	Purine metabolism，RNA polymerase， Pyrimidine metabolism
novel_mir161	CL5937.Contig2_All	-9.861	Purine metabolism，RNA polymerase， Pyrimidine metabolism，Cutin, suberine and wax biosynthesis
miR167d_1	Unigene9900_All	-6.6.35	Pentose phosphate pathway，Biosynthesis of amino acids，Biosynthesis of secondary metabolites，Carbon fixation in photosynthetic organisms，Carbon metabolism
miR167d_1	CL5786.Contig2_All	-6.6.35	Sphingolipid metabolism
miR167d_1	Unigene2643_All	-6.6.35	Pentose phosphate pathway，Biosynthesis of amino acids，Biosynthesis of secondary metabolites，Carbon fixation in photosynthetic organisms，Carbon metabolism
miR167d_1	Unigene8353_All	-6.6.35	Pentose phosphate pathway，Biosynthesis of amino acids，Biosynthesis

			of secondary metabolites，Carbon fixation in photosynthetic organisms，Carbon metabolism
miR167d_1	Unigene12094_All	-6.6.35	Pentose phosphate pathway，Biosynthesis of amino acids，Biosynthesis of secondary metabolites，Carbon fixation in photosynthetic organisms，Carbon metabolism
miR167d_1	CL1085.Contig8_All	-6.6.35	RNA transport;
miR396a-3p_1	CL5140.Contig4_All	-5.849	Spliceosome
miR396a-3p_1	CL10745.Contig5_All	-5.849	Glutathione metabolism
miR396a-3p_1	CL2219.Contig4_All	-5.849	Plant-pathogen interaction;
miR396a-3p_1	Unigene26657_All	-5.849	Plant-pathogen interaction
novel_mir22	Unigene12250_All	-2.346	Endocytosis，Phagosome
novel_mir22	CL451.Contig1_All	-2.346	Spliceosome
novel_mir22	Unigene11422_All	-2.346	Plant-pathogen interaction，Amino sugar and nucleotide sugar metabolism，Metabolic pathways，Alanine, aspartate and glutamate metabolism，Linoleic acid metabolism
novel_mir22	Unigene10264_All	-2.346	Ribosome biogenesis in eukaryotes，Spliceosome
novel_mir75	CL11927.Contig2_All	-1.599	Basal transcription factors
novel_mir75	CL9741.Contig4_All	-1.599	Ubiquitin mediated proteolysis;
miR397a_3	CL11442.Contig1_All	-1.273	Peroxisome，Citrate cycle (TCA cycle)，Biosynthesis of amino acids，2-Oxocarboxylic acid metabolism，Carbon metabolism，Glutathione metabolism;
novel_mir151	Unigene3477_All	-1.104	Spliceosome
novel_mir151	CL1290.Contig42_All	-1.104	Terpenoid backbone biosynthesis;

novel_mir151	CL11286.Contig9_All	-1.104	Glucosinolate biosynthesis， Cyanoamino acid metabolism， 2-Oxocarboxylic acid metabolism，RNA transport，Plant hormone signal transduction
novel_mir151	CL6270.Contig9_All	-1.104	Plant hormone signal transduction;
novel_mir151	Unigene11539_All	-1.104	Glycerophospholipid metabolism， Ether lipid metabolism， Inositol phosphate metabolism;
novel_mir151	CL8022.Contig1_All	-1.104	Pentose and glucuronate interconversions
novel_mir151	CL11930.Contig3_All	-1.104	Other types of O-glycan biosynthesis，Glycerophospholipid metabolism，Ether lipid metabolism，Inositol phosphate metabolism;
novel_mir151	CL12266.Contig2_All	-1.104	Spliceosome
novel_mir151	Unigene5040_All	-1.104	Spliceosome
novel_mir151	CL5804.Contig3_All	-1.104	Biosynthesis of secondary metabolites ,Steroid biosynthesis;
novel_mir151	Unigene7755_All	-1.104	Terpenoid backbone biosynthesis
novel_mir151	CL994.Contig6_All	-1.104	Spliceosome
novel_mir151	Unigene12079_All	-1.104	Terpenoid backbone biosynthesis

Table S21. Metabolic pathways of differentially expressed miRNAs in the CK vsM50

**miRNA id**	**Target id**	**log2(M50/CK)**	**Pathway**
novel_mir97	Unigene11539_All	9.589	Glycerophospholipid metabolism，Ether lipid metabolism, Inositol phosphate metabolism
novel_mir97	CL11756.Contig1_All	9.589	Purine metabolism
novel_mir97	CL11930.Contig3_All	9.589	Other types of O-glycan biosynthesis，Glycerophospholipid metabolism，Ether lipid metabolism，Inositol phosphate metabolism
miR160	CL11742.Contig5_All	8.759	Plant hormone signal transduction
miR396a-5p	CL623.Contig3_All	8.550	Endocytosis;
miR396a-5p	CL2267.Contig1_All	8.550	Homologous recombination
miR396a-5p	CL10458.Contig4_All	8.550	Lysine degradation

miR171b-3p	CL3157.Contig1_All	4.210	Plant hormone signal transduction
miR171b-3p	CL3157.Contig4_All	4.210	Plant hormone signal transduction
miR171b-3p	CL1203.Contig7_All	4.210	MAPK signaling pathway – plant，Plant hormone signal transduction
miR399b	Unigene8906_All	4.087	Ribosome
miR399b	CL2276.Contig1_All	4.087	Ubiquitin mediated proteolysis
miR399b	CL3187.Contig4_All	4.087	Alanine, aspartate and glutamate metabolism， Butanoate metabolism;
miR399b	CL11662.Contig2_All	4.087	Protein processing in endoplasmic reticulum
miR399a_6	CL2276.Contig2_All	2.709	Ubiquitin mediated proteolysis
miR399a_6	CL11662.Contig2_All	2.709	Protein processing in endoplasmic reticulum
miR168b_1	Unigene35241_All	1.949	Metabolic pathways，Ascorbate and aldarate metabolism;
miR168b_1	Unigene13184_All	1.949	Protein processing in endoplasmic reticulum，Protein export
miR168b_1	Unigene3288_All	1.949	Metabolic pathways，Ascorbate and aldarate metabolism
miR168b_1	Unigene3262_All	1.949	Metabolic pathways，Ascorbate and aldarate metabolism
miR168b_1	CL10842.Contig4_All	1.949	Metabolic pathways，Ascorbate and aldarate metabolism
miR168b_1	Unigene3326_All	1.949	Metabolic pathways，Ascorbate and aldarate metabolism
miR168b_1	Unigene12302_All	1.949	Metabolic pathways，Ascorbate and aldarate metabolism
novel_mir72	CL2494.Contig5_All	1.599	Other glycan degradation
novel_mir72	CL7956.Contig1_All	1.599	Phenylpropanoid biosynthesis
novel_mir72	Unigene19898_All	1.599	MAPK signaling pathway – plant，Plant-pathogen interaction
novel_mir72	Unigene6418_All	1.599	Plant hormone signal transduction
novel_mir72	CL4372.Contig2_All	1.599	Starch and sucrose metabolism
novel_mir72	Unigene7472_All	1.599	Plant hormone signal transduction
novel_mir72	Unigene3832_All	1.599	Plant hormone signal transduction
novel_mir72	CL2352.Contig11_All	1.599	ABC transporters
novel_mir72	Unigene11537_All	1.599	RNA transport，Ribosome biogenesis in eukaryotes

novel_mir72	CL2144.Contig6_All	1.599	Alanine, aspartate and glutamate metabolism，Valine, leucine and isoleucine degradation，Glycine, serine and threonine metabolism，Cysteine and methionine metabolism
novel_mir72	Unigene10328_All	1.599	Aminoacyl-tRNA biosynthesis
novel_mir72	CL11367.Contig6_All	1.599	Amino sugar and nucleotide sugar metabolism
novel_mir72	Unigene4211_All	1.599	Plant hormone signal transduction
novel_mir72	Unigene9743_All	1.599	Plant hormone signal transduction
novel_mir140	Unigene10722_All	1.462	Ribosome biogenesis in eukaryotes
miR396a-3p_5	Unigene15223_All	1.367	RNA transport
miR396a-3p_5	Unigene1142_All	1.367	Base excision repair
miR396a-3p_5	CL3506.Contig5_All	1.367	MAPK signaling pathway – plant，Plant-pathogen interaction
miR396a-3p_5	Unigene26657_All	1.367	Plant-pathogen interaction
miR396a-3p_5	Unigene32495_All	1.367	Base excision repair
miR396a-3p_5	CL9790.Contig1_All	1.367	RNA transport
miR396a-3p_5	CL3025.Contig1_All	1.367	Purine metabolism，RNA polymerase， Pyrimidine metabolism
miR396a-3p_5	CL10745.Contig5_All	1.367	Glutathione metabolism
miR396a-3p_5	CL2219.Contig7_All	1.367	Plant-pathogen interaction
miR396a-	CL5140.Contig7_All	1.367	Spliceosome

3p_5			
novel_mir176	Unigene5058_All	1.277	RNA degradation
novel_mir176	CL5246.Contig1_All	1.277	Basal transcription factors
novel_mir176	CL1212.Contig4_All	1.277	Alanine, aspartate and glutamate metabolism，Arginine biosynthesis，Biosynthesis of amino acids，Carbon fixation in photosynthetic organisms，2-Oxocarboxylic acid metabolism，Glycine, serine and threonine metabolism，Carbon metabolism，Glyoxylate and dicarboxylate metabolism
novel_mir176	Unigene5756_All	1.277	Pentose and glucuronate interconversions;
novel_mir176	Unigene2465_All	1.277	DNA replication
novel_mir176	CL10704.Contig2_All	1.277	Phosphatidylinosito signaling system，Glycerophospholipid metabolism，Glycerolipid metabolism
novel_mir176	Unigene10699_All	1.277	Spliceosome
novel_mir176	Unigene3222_All	1.277	Spliceosome
novel_mir176	Unigene6459_All	1.277	Spliceosome
novel_mir176	CL746.Contig32_All	1.277	Ubiquitin mediated proteolysis
novel_mir176	CL3878.Contig4_All	1.277	Pentose and glucuronate interconversions
novel_mir176	Unigene10704_All	1.277	Anthocyanin biosynthesis Isoflavonoid biosynthesis ，Flavone and flavonol biosynthesis，Arginine and proline metabolism
novel_mir176	Unigene10799_All	1.277	Spliceosome
novel_mir176	CL10443.Contig3_All	1.277	RNA degradation
novel_mir176	Unigene5877_All	1.277	Plant-pathogen interaction
novel_mir176	Unigene4275_All	1.277	Anthocyanin biosynthesis，Isoflavonoid biosynthesis，Flavone and flavonol biosynthesis，Arginine and proline metabolism
novel_mir176	Unigene30367_All	1.277	Plant-pathogen interaction
novel_mir176	CL12106.Contig7_All	1.277	Spliceosome
novel_mir176	CL11400.Contig1_All	1.277	Purine metabolism

novel_mir176	CL463.Contig6_All	1.277	DNA replication
novel_mir176	Unigene9281_All	1.277	Anthocyanin biosynthesis，Isoflavonoid biosynthesis，Flavone and flavonol biosynthesis，Arginine and proline metabolism
novel_mir176	Unigene3776_All	1.277	Plant-pathogen interaction
novel_mir176	CL11400.Contig7_All	1.277	Purine metabolism
novel_mir61	Unigene4832_All	1.207	RNA transport
novel_mir61	CL11652.Contig1_All	1.207	Biosynthesis of secondary metabolites，Phenylpropanoid biosynthesis
novel_mir61	CL6502.Contig1_All	1.207	mRNA surveillance pathway ，RNA transport
miR396a-3p_1	CL10745.Contig5_All	1.131	Glutathione metabolism
miR396a-3p_1	Unigene26657_All	1.131	Plant-pathogen interaction
miR396a-3p_1	CL2219.Contig9_All	1.131	Plant-pathogen interaction
novel_mir73	CL823.Contig4_All	1.130	Biosynthesis of amino acids，Phenylalanine, tyrosine and tryptophan biosynthesis
novel_mir11	CL11597.Contig3_All	1.090	Anthocyanin biosynthesis，Isoflavonoid biosynthesis，Flavone and flavonol biosynthesis，Arginine and proline metabolism
novel_mir125	CL1025.Contig6_All	1.078	Glycosylphosphatidylinositol (GPI)-anchor biosynthesis
novel_mir179	Unigene9281_All	1.047	Anthocyanin biosynthesis，Isoflavonoid biosynthesis，Flavone and flavonol biosynthesis，Arginine and proline metabolism
novel_mir179	Unigene7634_All	1.047	Pantothenate and CoA biosynthesis，Biosynthesis of secondary metabolites
novel_mir179	Unigene6459_All	1.047	Spliceosome
novel_mir179	Unigene5756_All	1.047	Pentose and glucuronate interconversions
novel_mir179	Unigene5058_All	1.047	RNA degradation

novel_mir179	Unigene4275_All	1.047	Anthocyanin biosynthesis，Isoflavonoid biosynthesis，Flavone and flavonol biosynthesis，Arginine and proline metabolism
novel_mir179	Unigene3222_All	1.047	Spliceosome
novel_mir179	Unigene2465_All	1.047	DNA replication
novel_mir179	Unigene12111_All	1.047	Glycolysis / Gluconeogenesis，Amino sugar and nucleotide sugar metabolism， Galactose metabolism，Fructose and mannose metabolism，Starch and sucrose metabolism，Carbon metabolism
novel_mir179	Unigene11802_All	1.047	Pentose and glucuronate interconversions
novel_mir179	Unigene10799_All	1.047	Spliceosome
novel_mir179	Unigene10699_All	1.047	Spliceosome
novel_mir179	CL8668.Contig14_All	1.047	Ribosome biogenesis in eukaryotes，Protein processing in endoplasmic reticulum
novel_mir179	CL463.Contig6_All	1.047	DNA replication
novel_mir179	CL4206.Contig8_All	1.047	Phenylpropanoid biosynthesis
novel_mir179	CL3878.Contig4_All	1.047	Pentose and glucuronate interconversions
novel_mir179	CL368.Contig8_All	1.047	Phagosome，Oxidative phosphorylation
novel_mir179	CL2997.Contig1_All	1.047	Glycolysis / Gluconeogenesis，Amino sugar and nucleotide sugar metabolism， Galactose metabolism，Fructose and mannose metabolism，Starch and sucrose metabol，Carbon metabolism
novel_mir179	CL12315.Contig15_All	1.047	Glycolysis / Gluconeogenesis
novel_mir179	CL1222.Contig4_All	1.047	Pantothenate and CoA biosynthesis
novel_mir179	CL11400.Contig1_All	1.047	Purine metabolism
novel_mir179	CL10467.Contig17_All	1.047	Amino sugar and nucleotide sugar metabolism，Metabolic pathways
miR160a-5p	CL8053.Contig1_All	-15.583	Plant hormone signal transduction
miR160a-5p	CL7275.Contig1_All	-15.583	Plant hormone signal transduction

novel_mir75	CL11927.Contig2_All	-12.060	Basal transcription factor
novel_mir75	CL9741.Contig4_All	-12.060	Ubiquitin mediated proteolysis
miR171b-3p-3	CL3157.Contig1_All	-10.891	Plant hormone signal transduction
miR171b-3p_3	CL3157.Contig4_All	-10.891	Plant hormone signal transduction
novel_mir160	Unigene7853_All	-10.689	Spliceosome
novel_mir79	Unigene19726_All	-10.264	Starch and sucrose metabolism，Cyanoamino acid metabolism，Phenylpropanoid biosynthesis
novel_mir79	CL1682.Contig15_All	-10.264	Starch and sucrose metabolism，Cyanoamino acid metabolism，Phenylpropanoid biosynthesis
novel_mir79	CL12266.Contig2_All	-10.264	Spliceosome
novel_mir79	CL1290.Contig42_All	-10.264	Terpenoid backbone biosynthesis
novel_mir79	Unigene12079_All	-10.264	Terpenoid backbone biosynthesis
novel_mir79	Unigene7755_All	-10.264	Terpenoid backbone biosynthesis
novel_mir79	CL994.Contig6_All	-10.264	Spliceosome
novel_mir79	CL8022.Contig1_All	-10.264	Pentose and glucuronate interconversions
novel_mir161	CL3265.Contig7_All	-9.861	Cysteine and methionine metabolism，Glutathione metabolism
novel_mir161	Unigene9188_All	-9.861	RNA degradation
novel_mir161	CL10762.Contig2_All	-9.861	Purine metabolism，RNA polymerase，Pyrimidine metabolism
novel_mir161	CL5937.Contig2_All	-9.861	Purine metabolism，RNA polymerase， Pyrimidine metabolism
novel_mir47	Unigene2403_All	-9.589	N-Glycan biosynthesis Metabolic pathways
novel_mir47	CL11930.Contig5_All	-9.589	Metabolic pathways，Fatty acid elongation，Fatty acid metabolism
miR167d_1	Unigene9900_All	-7.379	Pentose phosphate pathway，Biosynthesis of amino acids，Carbon fixation in photosynthetic organisms，Carbon metabolism，Phenylpropanoid biosynthesis

miR167d_1	Unigene8353_All	-7.379	Pentose phosphate pathway，Biosynthesis of amino acids，Carbon fixation in photosynthetic organisms ，Carbon metabolism
miR167d_1	Unigene2643_All	-7.379	Pentose phosphate pathway，Biosynthesis of amino acids，Carbon fixation in photosynthetic organisms，Carbon metabolism
miR167d_1	Unigene12094_All	-7.379	Pentose phosphate pathway，Biosynthesis of amino acids，Carbon fixation in photosynthetic organisms，Carbon metabolism
miR167d_1	CL5786.Contig3_All	-7.379	Sphingolipid metabolism
miR167d_1	CL1085.Contig8_All	-7.379	RNA transport
miR396b	CL623.Contig3_All	-4.162	Endocytosis
miR396b	CL5298.Contig2_All	-4.162	Other types of O-glycan biosynthesis
miR396b	CL3856.Contig4_All	-4.162	AGE-RAGE signaling pathway in diabetic complications
miR396b	CL2267.Contig1_All	-4.162	Homologous recombination
miR396b	CL1599.Contig7_All	-4.162	RNA transport
miR396b	CL10458.Contig4_All	-4.162	Lysine degradation
novel_mir56	CL5937.Contig55_All	-3.174	Cutin, suberine and wax biosynthesis
novel_mir56	CL2960.Contig3_All	-3.174	Circadian rhythm - plant
novel_mir138	CL1673.Contig4_All	-3.081	Linoleic acid metabolism，alpha-Linolenic acid metabolism
novel_mir13	Unigene4506_All	-2.987	Plant-pathogen interaction，Amino sugar and nucleotide sugar metabolism
novel_mir13	CL11652.Contig31_All	-2.987	Sesquiterpenoid and triterpenoid biosynthesis，Plant-pathogen interaction
novel_mir13	Unigene6986_All	-2.987	Sesquiterpenoid and triterpenoid biosynthesis，Plant-pathogen interaction，Amino sugar and nucleotide sugar metabolism
novel_mir13	Unigene4130_All	-2.987	Plant-pathogen interaction，Amino sugar and nucleotide sugar metabolism，Alanine, aspartate and glutamate metabolism，MAPK signaling pathway - plant

novel_mir13	Unigene11664_All	-2.987	Sesquiterpenoid and triterpenoid biosynthesis，Plant-pathogen interaction ，Amino sugar and nucleotide sugar metabolism
miR396h	CL623.Contig3_All	-2.198	Endocytosis
miR396h	CL5884.Contig4_All	-2.198	RNA transport
miR396h	CL5298.Contig2_All	-2.198	Other types of O-glycan biosynthesis
miR396h	CL2267.Contig1_All	-2.198	Homologous recombination
miR396h	CL10458.Contig4_All	-2.198	Lysine degradation，Lysine biosynthesis，Biosynthesis of amino acids，
novel_mir76	CL9741.Contig4_All	-2.129	Ubiquitin mediated proteolysis
novel_mir82	Unigene3546_All	-2.122	Cysteine and methionine metabolism，Diterpenoid biosynthesis
novel_mir45	Unigene12033_All	-1.657	RNA polymerase，Pyrimidine metabolism，Pentose and glucuronate interconversions
novel_mir148	Unigene5871_All	-1.555	Ribosome
novel_mir148	Unigene8713_All	-1.555	Ribosome
miR171d_1	CL9585.Contig3_All	-1.451	Circadian rhythm – plant，Ubiquitin mediated proteolysis
miR171d_1	CL3157.Contig1_All	-1.451	Plant hormone signal transduction
miR171d_1	CL3157.Contig4_All	-1.451	Plant hormone signal transduction
novel_mir42	CL2960.Contig3_All	-1.324	Circadian rhythm - plant
miR398b	CL4864.Contig1_All	-1.304	Purine metabolism，RNA polymerase， Pyrimidine metabolism
miR171f_3	CL3157.Contig1_All	-1.266	Plant hormone signal transduction
miR171f_3	CL3157.Contig4_All	-1.266	Plant hormone signal transduction
novel_mir63	Unigene9662_All	-1.178	Porphyrin and chlorophyll metabolism，Aminoacyl-tRNA biosynthesis，
novel_mir63	Unigene27384_All	-1.178	Ribosome
novel_mir63	Unigene27383_All	-1.178	Ribosome
novel_mir63	CL9365.Contig2_All	-1.178	Glycolysis / Gluconeogenesis，Pentose phosphate pathway，Biosynthesis of amino acids，Fructose and mannose

			metabolism，Carbon fixation in photosynthetic organisms，Carbon metabolism
novel_mir63	CL2369.Contig5_All	-1.178	Spliceosome
novel_mir63	CL2211.Contig3_All	-1.178	Endocytosis，Glycerophospholipid metabolism，Ether lipid metabolism
novel_mir175	CL3501.Contig3_All	-1.161	Spliceosome
novel_mir29	CL12229.Contig3_All	-1.150	Ribosome
novel_mir29	Unigene8173_All	-1.150	Ribosome
novel_mir29	CL968.Contig2_All	-1.150	Ribosome
novel_mir29	Unigene8536_All	-1.150	Ribosome
novel_mir4	CL74.Contig15_All	-1.088	Plant hormone signal transduction
novel_mir4	Unigene3862_All	-1.088	mRNA surveillance pathway
novel_mir4	Unigene36625_All	-1.088	mRNA surveillance pathway
novel_mir4	CL9183.Contig4_All	-1.088	Circadian rhythm - plant
novel_mir4	CL8870.Contig1_All	-1.088	Oxidative phosphorylation
novel_mir4	CL74.Contig27_All	-1.088	Plant hormone signal transduction
novel_mir4	CL5100.Contig3_All	-1.088	mRNA surveillance pathway
novel_mir4	CL4833.Contig8_All	-1.088	mRNA surveillance pathway
novel_mir4	CL329.Contig22_All	-1.088	Phagosome，Oxidative phosphorylation;
novel_mir4	CL12137.Contig9_All	-1.088	Ribosome

Table S22. Metabolic pathways of differentially expressed miRNAs in the CK vsM100

miRNA id	Target id	log2(M100/CK)	Pathway
miR396a-3p_4	CL5140.Contig8_All	10.884	Spliceosome
miR396a-3p_4	CL2219.Contig7_All	10.884	Plant-pathogen interaction
miR396a-	Unigene26657_All	10.884	Plant-pathogen interaction

3p_4			
miR396a-3p_4	CL10745.Contig7_All	10.884	Glutathione metabolism
novel_mir97	Unigene11539_All	10.644	Glycerophospholipid metabolism， Ether lipid metabolism，Inositol phosphate metabolism
novel_mir97	CL11756.Contig1_All	10.644	Purine metabolism
novel_mir97	CL11930.Contig3_All	10.644	Other types of O-glycan biosynthesis，Glycerophospholipid metabolism，Ether lipid metabolism，Inositol phosphate metabolism
miR160	CL11742.Contig5_All	7.629	Plant hormone signal transduction
miR171b-3p	CL1203.Contig7_All	3.766	MAPK signaling pathway – plant，Plant hormone signal transduction
miR171b-3p	CL3157.Contig1_All	3.766	Plant hormone signal transduction
miR171b-3p	CL3157.Contig4_All	3.766	Plant hormone signal transduction
miR399b	CL2276.Contig2_All	3.597	Ubiquitin mediated proteolysis
miR399b	Unigene8906_All	3.597	Ribosome
miR399b	CL11662.Contig2_All	3.597	Protein processing in endoplasmic reticulum
miR399b	CL3187.Contig8_All	3.597	Alanine, aspartate and glutamate metabolism，Butanoate metabolism
miR399a_6	CL2276.Contig2_All	2.479	Ubiquitin mediated proteolysis
miR399a_6	CL11662.Contig2_All	2.479	Protein processing in endoplasmic reticulum
novel_mir47	Unigene2403_All	2.295	N-Glycan biosynthesis，Metabolic pathways
novel_mir47	CL11930.Contig5_All	2.295	Metabolic pathways，Fatty acid elongation，Fatty acid metabolism
novel_mir72	Unigene9743_All	1.999	Plant hormone signal transduction
novel_mir72	Unigene7472_All	1.999	Plant hormone signal transduction
novel_mir72	Unigene6418_All	1.999	Plant hormone signal transduction
novel_mir72	Unigene4211_All	1.999	Plant hormone signal transduction

novel_mir72	Unigene3832_All	1.999	Plant hormone signal transduction
novel_mir72	Unigene19898_All	1.999	MAPK signaling pathway - plant，Plant-pathogen interaction
novel_mir72	Unigene11537_All	1.999	RNA transport，Ribosome biogenesis in eukaryotes
novel_mir72	Unigene10328_All	1.999	Aminoacyl-tRNA biosynthesis
novel_mir72	CL7956.Contig1_All	1.999	Phenylpropanoid biosynthesis
novel_mir72	CL4372.Contig2_All	1.999	Starch and sucrose metabolism
novel_mir72	CL2494.Contig1_All	1.999	Other glycan degradation
novel_mir72	CL2352.Contig11_All	1.999	ABC transporters
novel_mir72	CL2144.Contig5_All	1.999	Alanine, aspartate and glutamate metabolism，Valine, leucine and isoleucine degradation，Glycine, serine and threonine metabolism，Cysteine and methionine metabolism;
novel_mir72	CL11367.Contig4_All	1.999	Amino sugar and nucleotide sugar metabolism
miR171 f_3	CL3157.Contig1_All	1.368	Plant hormone signal transduction
miR171f_3	CL3157.Contig4_All	1.368	Plant hormone signal transduction
novel_mir11	CL11597.Contig3_All	1.334	Anthocyanin biosynthesis，Isoflavonoid biosynthesis，Flavone and flavonol biosynthesis，Arginine and proline metabolism
novel_mir62	Unigene8140_All	1.248	Aminoacyl-tRNA biosynthesis
novel_mir62	CL11634.Contig12_All	1.248	Alanine, aspartate and glutamate metabolism，Arginine biosynthesis，Biosynthesis of amino acids
novel_mir62	CL11827.Contig3_All	1.248	Alanine, aspartate and glutamate metabolism，Pyrimidine metabolism
novel_mir62	Unigene26594_All	1.248	beta-Alanine metabolism，Alanine, aspartate and glutamate metabolism，Taurine and hypotaurine metabolism，Butanoate metabolism
novel_mir62	CL4469.Contig2_All	1.248	Porphyrin and chlorophyll metabolism Biosynthesis of secondary metabolites
novel_mir62	CL651.Contig3_All	1.248	Vancomycin resistance，Metabolic pathways

novel_mir62	Unigene7005_All	1.248	Aminoacyl-tRNA biosynthesis
miR396a-3p_5	Unigene32495_All	1.106	Base excision repair
miR396a-3p_5	Unigene26657_All	1.106	Plant-pathogen interaction
miR396a-3p_5	Unigene15223_All	1.106	RNA transport
miR396a-3p_5	Unigene1142_All	1.106	Base excision repair
miR396a-3p_5	CL9790.Contig2_All	1.106	RNA transport
miR396a-3p_5	CL5140.Contig7_All	1.106	Spliceosome
miR396a-3p_5	CL3506.Contig5_All	1.106	MAPK signaling pathway - plant，Plant-pathogen interaction;
miR396a-3p_5	CL3025.Contig13_All	1.106	Purine metabolism，RNA polymerase，Pyrimidine metabolism
miR396a-3p_5	CL2219.Contig7_All	1.106	Plant-pathogen interaction
miR396a-3p_5	CL10745.Contig2_All	1.106	Glutathione metabolism
novel_mir101	Unigene7152_All	1.071	Pentose and glucuronate interconversions，Fructose and mannose metabolism
novel_mir101	CL11827.Contig8_All	1.071	Alanine, aspartate and glutamate metabolism，Pyrimidine metabolism
novel_mir101	CL8130.Contig17_All	1.071	Lysine degradation
novel_mir101	Unigene10661_All	1.071	Pentose and glucuronate interconversions，Fructose and mannose

			metabolism
miR160a-5p	CL8053.Contig1_All	-15.583	Plant hormone signal transduction
miR160a-5p	CL11742.Contig2_All	-15.583	Plant hormone signal transduction
miR160a-5p	CL7275.Contig2_All	-15.583	Plant hormone signal transduction
miR167d_1	Unigene9900_All	-12.086	Pentose phosphate pathway，Biosynthesis of amino acids，Carbon fixation in photosynthetic organisms，Carbon metabolism
miR167d_1	Unigene8353_All	-12.086	Pentose phosphate pathway，Biosynthesis of amino acids，Carbon fixation in photosynthetic organisms，Carbon metabolism
miR167d_1	Unigene2643_All	-12.086	Pentose phosphate pathway，Biosynthesis of amino acids，Carbon fixation in photosynthetic organisms，Carbon metabolism
miR167d_1	Unigene12094_All	-12.086	Pentose phosphate pathway，Biosynthesis of amino acids，Carbon fixation in photosynthetic organisms，Carbon metabolism
miR167d_1	CL5786.Contig2_All	-12.086	Sphingolipid metabolism，Metabolic pathway
miR167d_1	CL1085.Contig8_All	-12.086	RNA transport;
miR171 b-3p_3	CL3157.Contig1_All	-5.248	Plant hormone signal transduction
miR171b-3p_3	CL3157.Contig4_All	-5.248	Plant hormone signal transduction
miR6300	Unigene36691_All	-2.857	Homologous recombination
miR6300	Unigene14900_All	-2.857	Phenylpropanoid biosynthesis
miR6300	CL6146.Contig4_All	-2.857	Spliceosome
miR6300	CL3661.Contig14_All	-2.857	Ubiquitin mediated proteolysis
miR6300	CL3368.Contig6_All	-2.857	Fatty acid biosynthesis，Fatty acid metabolism，Biosynthesis of unsaturated fatty acids;
miR6300	CL2760.Contig1_All	-2.857	Homologous recombination
miR6300	CL2696.Contig10_All	-2.857	Alanine, aspartate and glutamate metabolism，Nitrogen metabolism，Arginine biosynthesis

miR6300	CL11946.Contig2_All	-2.857	Endocytosis
miR6300	CL11863.Contig1_All	-2.857	Carbon fixation in photosynthetic organisms，Pyruvate metabolism，Carbon metabolism
novel_mir22	Unigene12250_All	-2.492	Endocytosis，Phagosome
novel_mir22	CL451.Contig1_All	-2.492	Spliceosome
novel_mir22	Unigene11422_All	-2.492	Plant-pathogen interaction，Alanine, aspartate and glutamate metabolism，Amino sugar and nucleotide sugar metabolism，Linoleic acid metabolism
novel_mir22	Unigene10264_All	-2.492	Ribosome biogenesis in eukaryotes，Spliceosome
novel_mir148	Unigene5871_All	-1.549	Ribosome
novel_mir148	Unigene8713_All	-1.549	Ribosome
novel_mir151	Unigene3477_All	-1.363	Spliceosome
novel_mir151	CL1290.Contig42_All	-1.363	Terpenoid backbone biosynthesis
novel_mir151	Unigene7755_All	-1.363	Terpenoid backbone biosynthesis
novel_mir151	Unigene5040_All	-1.363	Spliceosome
novel_mir151	Unigene12079_All	-1.363	Terpenoid backbone biosynthesis
novel_mir151	Unigene11539_All	-1.363	Glycerophospholipid metabolism，Ether lipid metabolism，Inositol phosphate metabolism
novel_mir151	CL994.Contig5_All	-1.363	Spliceosome
novel_mir151	CL8022.Contig1_All	-1.363	Pentose and glucuronate interconversions，Metabolic pathways
novel_mir151	CL6270.Contig9_All	-1.363	Plant hormone signal transduction
novel_mir151	CL5804.Contig3_All	-1.363	Steroid biosynthesis;
novel_mir151	CL2947.Contig1_All	-1.363	Spliceosome
novel_mir151	CL12266.Contig2_All	-1.363	Spliceosome
novel_mir151	CL11930.Contig3_All	-1.363	Other types of O-glycan biosynthesis，Glycerophospholipid metabolism，Ether lipid metabolism，Inositol phosphate metabolism

novel_mir151	CL11286.Contig5_All	-1.363	Glucosinolate biosynthesis，Cyanoamino acid metabolism，2-Oxocarboxylic acid metabolism，Plant hormone signal transduction
novel_mir172	Unigene9713_All	-1.312	Oxidative phosphorylation，Photosynthesis;
novel_mir172	CL947.Contig1_All	-1.312	Oxidative phosphorylation，Photosynthesis;
novel_mir138	CL1673.Contig5_All	-1.187	Linoleic acid metabolism，alpha-Linolenic acid metabolism
